# Triarylamminium Radical Cation Facilitates the Deprotection of *tert*-Butyl Groups in Esters, Ethers, Carbonates, and Carbamates

**DOI:** 10.1021/acs.joc.3c00238

**Published:** 2023-05-01

**Authors:** Denisa Hidasová, Tomáš Slanina

**Affiliations:** Institute of Organic Chemistry and Biochemistry of the Czech Academy of Sciences, Flemingovo nám. 2, 166 10 Prague, Czech Republic

## Abstract

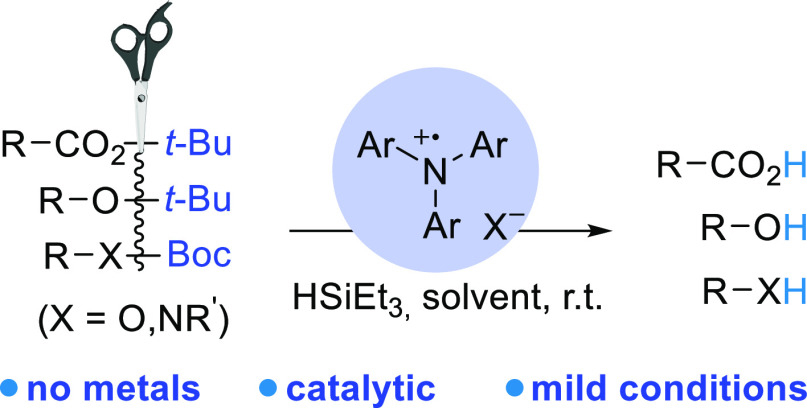

We report a catalytic protocol for mild O*t*Bu deprotection using two commercial reagents: the tris-4-bromophenylamminium radical cation, commonly known as magic blue (MB^•+^), and triethylsilane. Magic blue catalytically facilitates the cleavage of the C–O bond in *tert-*butyl carbamates, carbonates, esters, and ethers in a high isolated yield of up to 95%, and sacrificial triethylsilane accelerates the reaction. Without requiring high temperatures, transition metals, or strong acidic or basic catalysts, this method is suitable for structurally diverse compounds, including aliphatic, aromatic, and heterocyclic substrates.

## Introduction

Protecting groups are crucial tools for the synthesis of highly chemo- and regioselective complex organic molecules and biopolymers. Suitable protecting groups must be selected for each synthesis because they influence the length and efficiency of a multistep synthetic procedure. In other words, the protection and deprotection steps must be selective and high yielding.^1^

A wide range of protecting groups are currently available for different functional groups.^1,2^ However, molecules with sensitive functional groups or a unique framework most often cannot be masked by protecting groups that require harsh deprotection conditions (e.g., high temperatures). Therefore, the deprotection of such “problematic” functional groups requires alternative, mild, and reliable methods.

Among protecting groups, *tert*-butyl (*t*Bu) stands out as one of the most powerful for masking carboxylic acids and alcohols^2^ and is accordingly widely used in organic synthesis. However, deprotecting the *tert*-butyl moiety usually requires harsh conditions, such as strong Brønsted- or Lewis acids,^2,3^ acidic fluorinated alcohols as a solvent at 100 °C,^4,5^ stoichiometric amounts of CeCl_3_ and NaI in CH_3_CN at 40–70 °C,^6,7^ and a reagent-free continuous plug flow reactor at 120–240 °C.^8^ These harsh conditions limit our ability to use *t*Bu to mask sensitive synthetic intermediates.

Notwithstanding the above, two methods have been reported in the literature for the catalytic cleavage of the C–O bond of *Ot*Bu groups in carbamates, carbonates, esters, and ethers. One method uses a transition metal complex (μ^3^,η^2^,η^3^,η^5^-acenaphthylene)Ru_3_(CO)_7_ with the stoichiometric silane derivative PhMe_2_SiH under mild and neutral conditions.^9^ The other method combines PdCl_2_ with activated carbon and 1,1,3,3-tetramethyldisiloxane.^10^ Yet, removing metallic trace impurities from products remains a complex and tedious process. Moreover, the low accessibility of transition metal catalysts prevents the use of such methods in common laboratory practice.

Herein, we report a transition-metal-free, gentle, and easy method for C–O bond deprotection in O*t*Bu groups in carbamates, carbonates, esters, and ethers using catalytic amounts of the tris-4-bromophenylamminium cation radical, also known as magic blue (MB^•+^), and hydrosilane. This method is also compatible with compounds containing other ester groups and olefinic moieties, which are not reduced during the reaction. Since the experimental procedure is simple and purification is easy, our catalytic protocol may be applied to a wide range of organic synthesis reactions.

## Results and Discussion

### Magic Blue Chemistry

The tris-4-bromophenylamminium cation radical (MB^•+^) is a commercially available reagent commonly used as a single-electron oxidant and/or an acid generator^11^ in various synthetic transformations, including protecting group removal (*para*-methoxybenzyl ether,^12^ tetrahydropyranyl (THP) ether,^13^ dithioketal,^14^ and silyl ethers^13^), glycosylations,^15^ and radical rearrangements,^16^ as well as in a high number of radical cation-mediated [4 + 2],^17^ [2 + 2],^18^ and [3 + 2]^19^ cycloaddition reactions.^11^ In addition, MB^•+^ has been shown to oxidize various tertiary amines,^20,21^ electron-rich aromatics,^22−25^ and enolates^26^ and to mediate Markovnikov hydration of (*E*)-aryl enynes to the corresponding enones.^27^

### Discovery and Optimization of Reaction Conditions

While attempting hydrosilylation of olefins, we serendipitously observed that MB^•+^ and triethylsilane acted as de-*tert*-butylation reagents for *tert*-butyl acrylates in dichloromethane (DCM) without polymerization or reduction of the double bond (Table 1). MB^•+^ was essential to the reaction (Table 1, entry 1), but 10 mol % resulted in a low yield of the desired acrylic acid (Table 1, entry 2). Nevertheless, increasing the amount of MB^•+^ increased the yields of carboxylic acid when performing the reaction in dichloromethane (Table 1, entries 3 and 4). Using a stoichiometric amount of MB^•+^ resulted in quantitative conversions of *tert*-butyl acrylate (Table 1, entry 5), but 50 and 30 mol % MB^•+^ with 2 equiv of triethysilane in acetonitrile sufficed to achieve a quantitative yield of deprotection after only 1 h (Table 1, entries 6 and 7). Further decreasing the catalytic loading of MB^•+^ to 10 mol % led to incomplete conversion (Table 1, entry 8). The relatively high catalytic loading of MB^•+^ needed for quantitative de-*tert*-butylation may be explained by its slow thermal reduction in acetonitrile or DCM. This thermal reduction was confirmed by gradual decoloration of the reaction mixture (Figures S1 and S2). After 8 h of stirring in MeCN, MB^•+^ is completely reduced to tris(4-bromophenyl)amine, while quantitative reduction in DCM takes ∼6 days. In addition, we found that **1a** reacts only with MB^•+^ to slowly yield **2a** (Table S1). Increasing the reaction rate required using silane as a sacrificial reagent. Dimethylphenylsilane was also effective as a reagent for this reaction (Table S1).

**Table 1:**
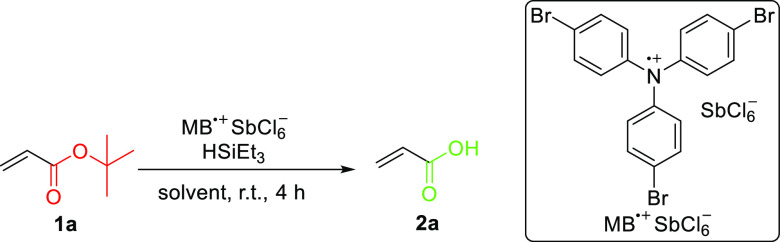
Optimization of Catalytic De-*tert*-butylation^a^

entry	MB^•+^ (mol %)	HSiEt_3_ (equiv)	solvent	yield of **2a** (%)^b^
1	0	2	CH_2_Cl_2_	0
2	10	2	CH_2_Cl_2_	28
3	30	2	CH_2_Cl_2_	63
4	50	2	CH_2_Cl_2_	85
5	100	2	CH_2_Cl_2_	99
6	50	2	CH_3_CN	99
**7**	**30**	**2**	**CH**_**3**_**CN**	**99**^c^
8	10	2	CH_3_CN	56^d^

a Unless otherwise specified, the reactions were performed using **1a** (0.068 mmol), **MB**^**•+**^, **HSiEt**_**3**_**,** and the solvent (0.5 mL) at room temperature.

b The yield of **2a** was determined by the ^1^H NMR of the crude mixture.

c Reaction yield after 1 h.

d Reaction yield after 4 h; after 1 h, 50% of **2a** is formed.

### Reaction Scope: Different Functional Groups

We further tested the deprotection of other *tert*-butyl esters and *tert*-butyl ethers, *O*-Boc, and *N*-Boc derivatives (Table 2). The *tert*-butyl esters were quantitatively converted into the corresponding carboxylic acids (Table 2, entries 1–5). A detailed kinetic study showed that quantitative deprotection is achieved within 40 min (Figure S3).

**Table 2:**
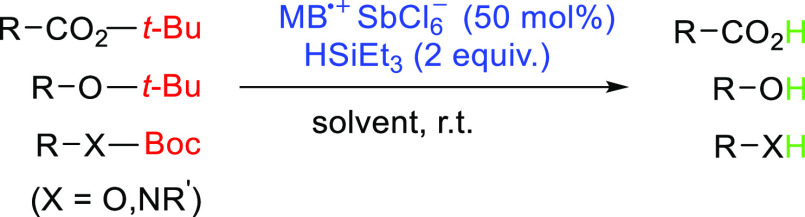

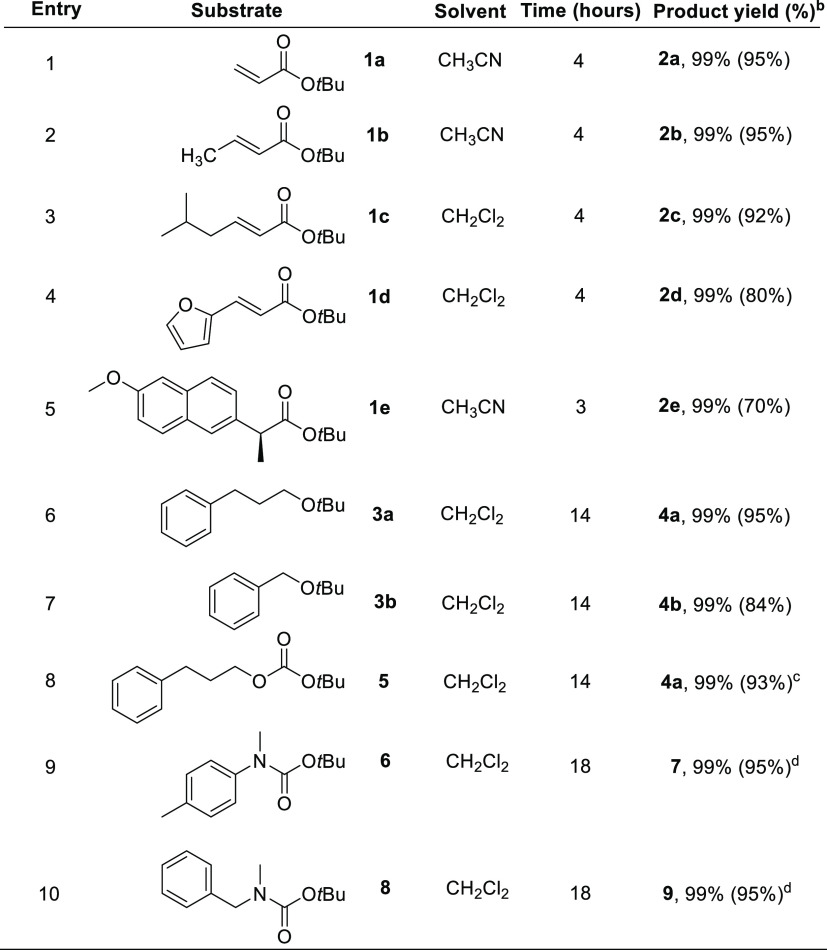
Deprotection of *tert*-Butyl Esters, *tert*-Butyl Ethers, *O*-Boc, and *N*-Boc Derivatives^a^

a Unless otherwise specified, the reactions were performed using **1a–e**, **3a**,**b**, **5**, **6**, **8** (0.34 mmol), **MB**^**•+**^ (0.17 mmol), **HSiEt**_**3**_ (0.68 mmol), and the **solvent** (2.5 mL) at room temperature.

b Reaction yield based on ^1^H NMR, and isolated yield in brackets.

c Reaction performed on a 1 mmol scale, according to the general procedure, yield: 93%, 0.93 mmol, 127 mg.

d 4 equiv of HSiEt_3_ was needed for quantitative conversion.

The method was very mild; even the α,β-unsaturated acids were not reduced, and the ester with the α-methyl group did not racemize upon deprotection (Table 2, entry 5). The *tert*-butyl ethers were de-*tert*-butylated to the corresponding alcohols in almost quantitative yields under the same reaction conditions (Table 2, entries 6,7). Deprotection of the *O*Boc derivative also proceeded smoothly within 14 h (Table 2, entry 8). In general, the deprotection rate decreased in the following order: *tert*-butyl esters > *tert*-butyl carbonates > *tert*-butyl ethers (Figure S4). The *N*-methyl-*N*-Boc derivatives were even less reactive than the *tert*-butyl ethers, requiring 4 equiv of triethylsilane to achieve high yields of the desired product (Table 2, entries 9 and 10).

### Chemoselectivity

**Scheme 1:**
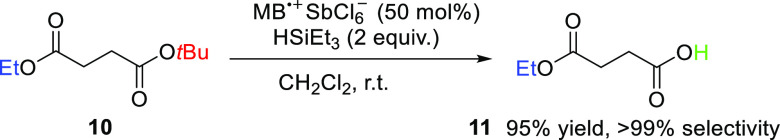
Selective Deprotection of *tert*-Butyl Ester in *tert*-Butyl Ethyl Succinate

We demonstrated the chemoselectivity of this method by selectively deprotecting *tert*-butyl ester in *tert*-butyl ethyl succinate **10**, which was converted into mono ethyl succinate **11** in 95% isolated yield as a single product (Scheme 1).

**Scheme 2:**
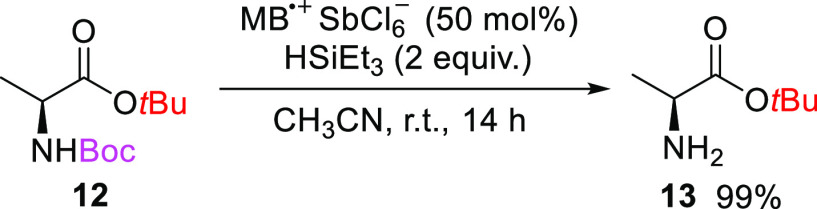
Deprotection of the *tert*-Butoxycarbonyl Group in *N*-[(1,1-Dimethylethoxy)carbonyl]-l-alanine 1,1-Dimethylethyl Ester

In doubly *tert*-butyl-protected *N-*Boc-l-alanine *tert*-butyl ester **12**, the *N*-Boc group was deprotected (Scheme 2) faster than the *t*Bu ester, most likely due to its activation by the α-carboxylic group.

**Scheme 3:**
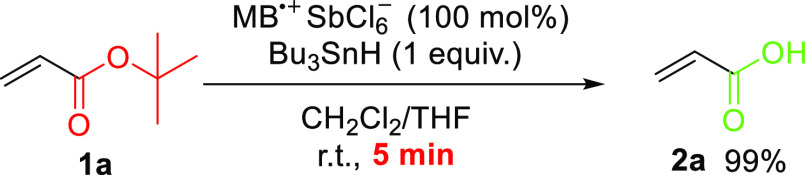
MB^•+^ and Tributyltin Hydride Mediate the Rapid Cleavage of the C–O Bond in O*t*Bu Groups in *tert*-Butyl Acrylate

When we replaced hydrosilane by tributyltin hydride, the cleavage of the C–O bond in *tert*-butyl esters proceeded within 5 min (Scheme 3). De-*tert*-butylation did not work without MB^•+^ or tributyltin hydride, as both components were needed for de-*tert-*butylation to occur (Table S2). The combination of MB^•+^ with tributyltin hydride is known to promote hole transfer hydrogenation of electron-rich alkenes (e.g., 1,1-diphenylethylene), including single-electron oxidation of the double bond.^28^ In our method, the electron-deficient acrylate double bond remains intact.

**Scheme 4:**
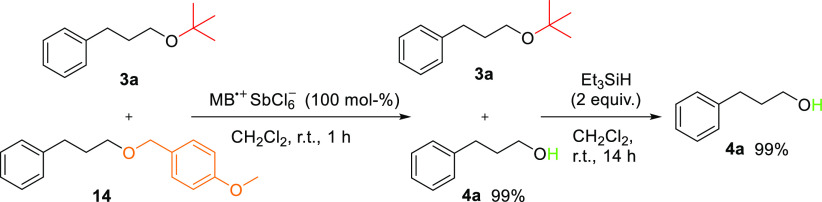
Chemoselective Deprotection of *tert*-Butyl and *p*-Methoxybenzyl Protecting Groups

As mentioned above, MB^•+^ has been previously reported as a deprotection agent of the *p*-methoxybenzyl ether-protecting group.^12^ In this process, MB^•+^ serves as an electron-transfer reagent (mediator), and ether cleavage occurs in moist acetonitrile. In our chemoselectivity screening, only 50 mol % of MB^•+^ was needed to deprotect the *p*-methoxybenzyl ether protective group. Therefore, we performed chemoselective deprotections of the *p*-methoxy benzyl group and the *tert*-butyl protecting group as a competition experiment in the same reaction vessel (Scheme 4). With 1 equiv of MB^•+^, the *p*-methoxybenzyl ether was deprotected first (consuming 50 mol % for deprotection), and *tert*-butyl ether was then deprotected after adding 2 equiv of triethylsilane to the reaction mixture.

**Scheme 5:**
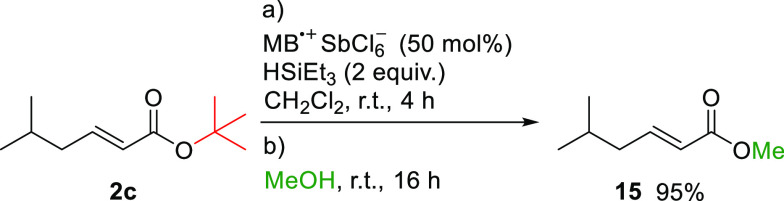
One-Pot *trans*-Esterification Mediated by MB^•+^/Triethylsilane

MB^•+^ can also mediate de-*tert*-butylation and subsequently activate the carboxylic acid formed *in situ* to facilitate esterification by adding an alcohol to the reaction mixture (Scheme 5).

**Scheme 6:**
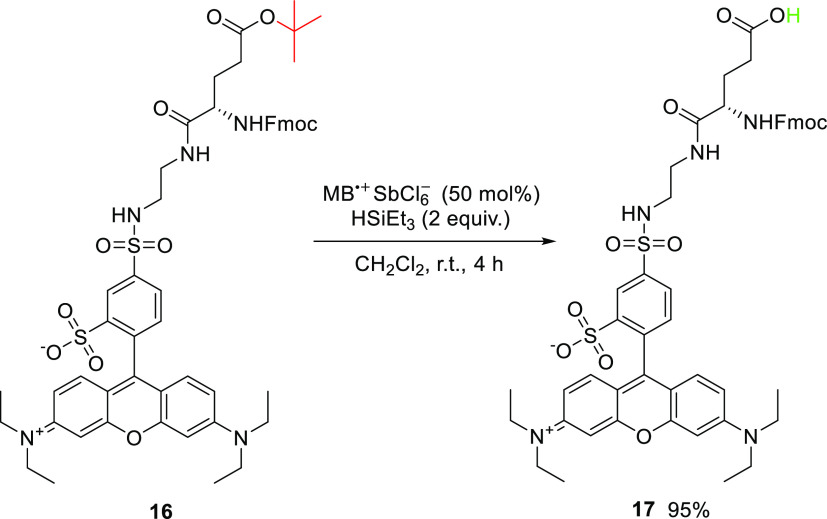
MB^•+^-Mediated De-*tert*-butylation of a Complex Substrate: Sulforhodamine Dye **16**

**Scheme 7:**
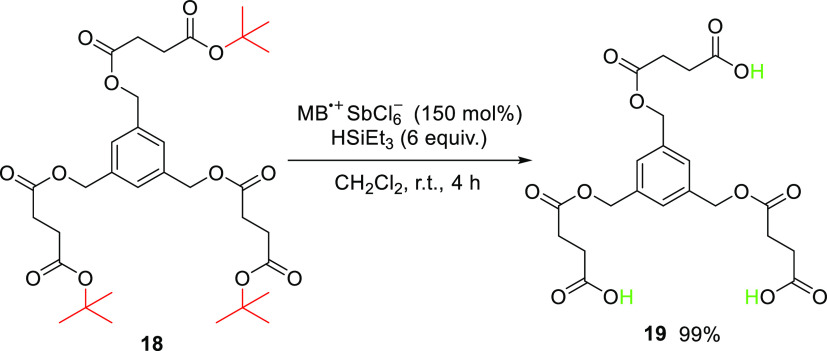
MB^•+^-Mediated De-*tert*-butylation of Substrate **18**

To demonstrate the universality and high functional-group tolerance of the deprotection strategy, we applied our de-*tert*-butylation method to a complex sulforhodamine B derivative **16** (Scheme 6) and to a polyfunctional substrate **18** (Scheme 7). The results showed a clean transformation of **16** to **17** and multiple de-*tert*-butylations of triester **18**.

Our deprotection method not only tolerated substrates containing keto, nitro, nitrile, and amide functional groups (Table S3) but also mediated the deprotection of prenyl esters and other acid-labile groups such as MOM, THP, and trityl-protected alcohols at room temperature, in addition to the *tert*-butyl group (Table S4). For this reason, we compared our method with the stoichiometric addition of a strong Brønsted acid (trifluoroacetic acid) to assess whether MB^•+^ and silane acted solely as acid generators in our system (Table S5). With 2 equiv of trifluoroacetic acid, de-*tert* butylation did not occur. By contrast, the desired product was quantitatively formed in the control reaction with MB^•+^ and triethylsilane.

### Mechanistic Insights

MB^•+^ is a commercial reagent known for its dual reactivity as a single-electron oxidant^11,29^ and as an acid generator.^11,13,14^ Both modes of action may help to explain its reactivity. However, neither of them is likely to occur, as discussed below.

In our mechanistic studies, we focused on a model reaction, de-*tert-*butylation of *tert*-butyl acrylate. The redox potential of MB^•+^ (*E* = 0.70 V vs Fc^0/+^ in dichloromethane solution)^29^ is too low for substrate or silane oxidation (*E*(**1a**) > 2.2 V vs Fc^0/+^;^30^*E*(HSiEt_3_) = ∼1.4 V vs 0.1 M Ag/AgNO_3_,^31^ which corresponds to ∼1.36 V vs Fc^0/+^).^32^ So, while electron transfer between triarylamminium radical cations and substrates with slightly more positive redox potentials (by 0.2–0.5 V) has been previously observed,^33,34^ this process is irrelevant in our system with a potential difference of >0.7 V. Unlike in the reaction with *p*-methoxybenzyl-protected substrates (Scheme 4), where MB^•+^ is quantitatively reduced by single-electron oxidation of the electron rich aryl moiety, MB^•+^ is not reduced in de-*tert* butylation because the reaction mixture remains blue throughout the reaction, thus ruling out the role of MB^•+^ as an oxidant in this process.

Alternatively, MB^•+^ may generate acid either by counterion decomposition^11^ or by hydrogen atom abstraction from a donor.^35^ Replacing MB^•+^ SbCl_6_^-^ by the analogous salt with hexafluorophosphate as a counterion did not influence the reaction rate. Conversely, the reaction with tetrabutylammonium hexafluorophosphate did not yield any product (Scheme S1). The counterion clearly does not play a role in the deprotection reaction.

In various solvents with hydrogen atoms available for abstraction, namely THF, isopropanol, dimethylformamide, and acetonitrile, MB^•+^ solutions gradually lost their color, subsequently forming the reduced triaryl amine. Discoloration, enhanced by light, adversely affected the deprotection yield, which is the most likely reason for the relatively high catalytic loading of MB^•+^ in our method.

We further tested the reaction mixture for changes in pH. The aqueous wash of the reaction mixture at full conversion became slightly acidic (pH ∼6). Isobutene, formed by deprotonation of the *tert*-butyl group, was the main by-product of de-*tert*-butylation. To examine the role of the Brønsted acid, we performed a control reaction with 2 equiv of trifluoroacetic acid (Table S5). This strong acid should provide a much higher concentration of protons than that released in the reaction with 50 mol% of MB^•+^. However, no deprotection of *tert*-butyl groups was observed under these conditions. Consequently, even though some acid is formed in the reaction mixture, its amount is nowhere near the concentration needed for an efficient, acid-mediated de-*tert*-butylation reaction.

**Figure 1:**
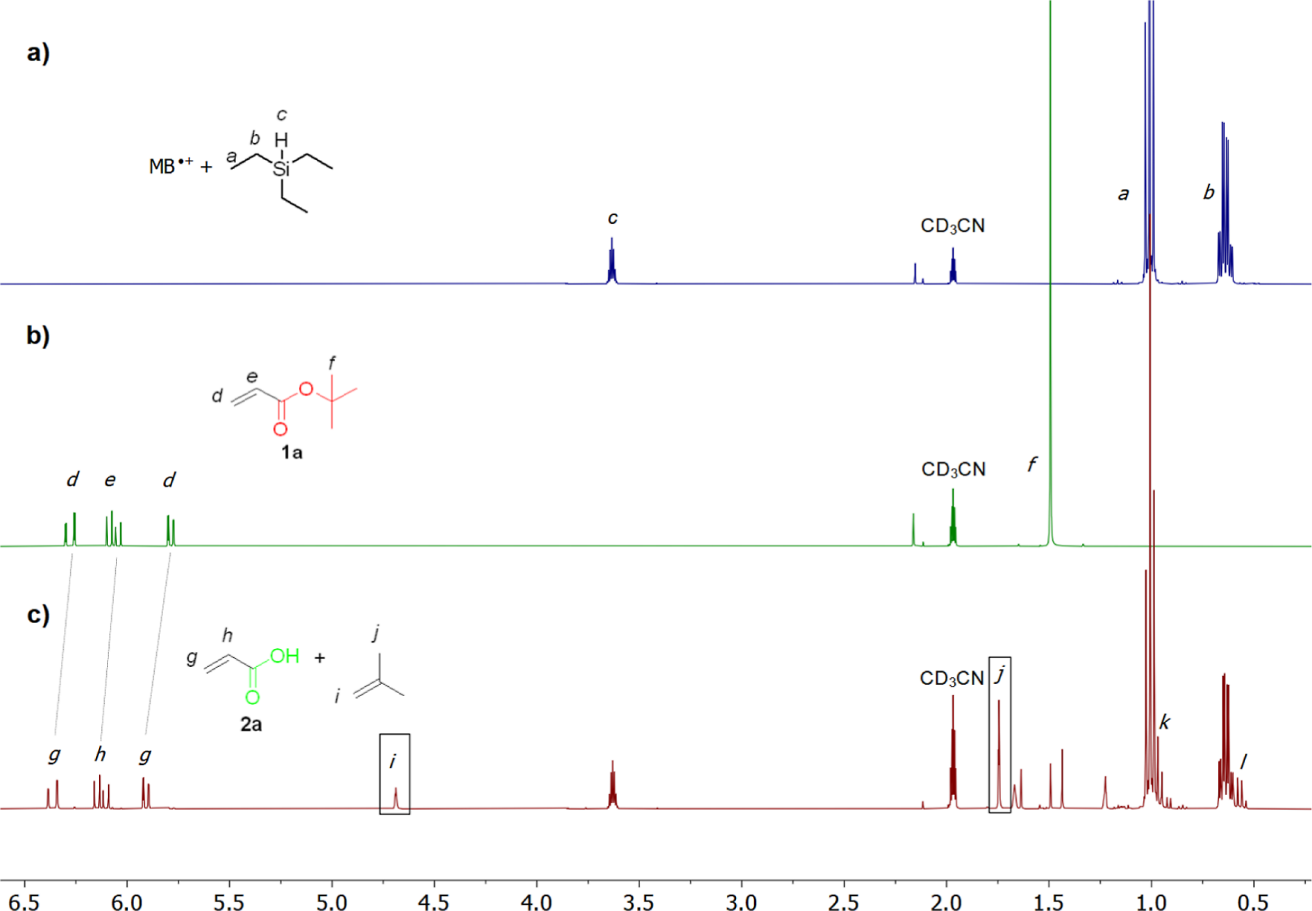
^1^H NMR in CD_3_CN of a mixture of (a) MB^•+^ (*c* = 0.05 mM) and HSiEt_3_ (*c* = 0.2 mM), (b) **1a** (*c* = 0.1 mM), (c) a reaction mixture of MB^•+^, HSiEt_3_, and **1a** after 1 h of reacting. Isobutene signals *i* and *j* are highlighted by rectangles.

The main argument supporting the role of MB^•+^ as a catalyst and not as an oxidant/acid generator is the sub-stoichiometric loading of MB^•+^ (30 mol %) sufficient for the full conversion. Reduced MB^•+^ cannot be regenerated under our conditions and, thus, must remain in its parent oxidized form throughout the catalytic cycle. Our NMR studies (Figures 1, S5–S6) showed that no complex is formed between the silane and MB^•+^ and that the reaction occurs only after adding the substrate (Figure 1a, no transfer of magnetization was observed between paramagnetic MB^•+^ and the silane signals). No signals of the reduced tris(4-bromophenyl)amine were observed throughout the reaction. The *tert-*butyl group was released as gaseous isobutene, as detected in the sealed NMR cuvette (Figure 1c, signals *i* and *j*). The de-*tert*-butylation product, which was formed as a silyl ester (Figure 1c, signals *k* and *l*), hydrolytically converted into the final product upon aqueous workup of the reaction mixture.

The formation of silyl ester from a hydrosilane is formally accompanied by the loss of a hydride, which reacts with protons to form dihydrogen. To demonstrate the formation of H_2_ in the reaction mixture, we ran a control experiment where hydrosilane was completely consumed to form a silyl ester. When adding palladium on charcoal, the acrylate double bond was immediately reduced (Figure S7). In the absence of palladium, the olefins were not reduced, thereby showing that olefins are not hydrogenated in our method. As such, our method is more advantageous than the de-*tert*-butylation previously described by Motoyama et al.^10^

**Scheme 8:**
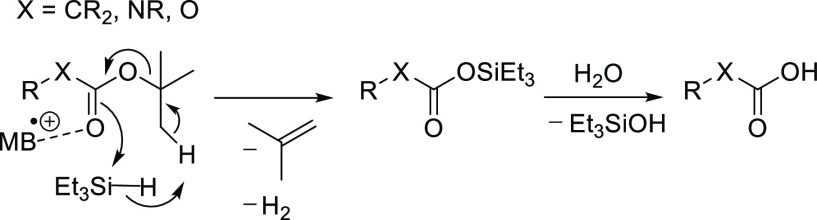
Tentative Mechanism of MB^•+^-Mediated De-*tert*-butylation

In summary, MB^•+^ did not act via any commonly known mechanism of oxidation or acid generation. Some Brønsted acid was formed, but nowhere near the amount needed for acid-mediated de-*tert*-butylation. De-*tert*-butylation yielded isobutene , and hydrosilane formed a hydrolytically labile silyl ester. Dihydrogen was indirectly detected in the reaction mixture using palladium as a catalyst for olefin hydrogenation. Based on these findings, we suggest the mechanism shown in Scheme 8, which is similar to the mechanism of ruthenium-catalyzed de-*tert*-butylation described by Nagashima et al.^9^ In this mechanism, MB^•+^ acts as a Lewis acid and forms a non-covalent complex with the substrate (Figure S8), whereas trialkyl hydrosilane assists the departure of isobutene during the formation of silyl ester and dihydrogen.

## Conclusions

In combination with triethylsilane, the inexpensive, commercially available, and easily handled reagent tris-4-bromophenylamminium cation radical (magic blue, MB^•+^) mediates the catalytic de-*tert*-butylation of *tert*-butyl esters, *tert*-butyl ethers, *O*-Boc, and *N*-Boc derivatives. In this mild deprotection method, MB^•+^ catalyzes the activation of Si–H bonds, leading to the deprotection of O*t*Bu groups. Because these reagents are easily accessible and the side-products are not toxic, this simple *tert*-butyl deprotection method may find applications in common laboratory procedures.

## Data Availability

The data underlying this study are available in the published article and its Supporting Information.

## Supporting Information Available

Experimental synthetic procedures, mechanistic control experiments, and ^1^H and ^13^C NMR spectra (PDF)
