# Supplementary material for: Triarylamminium Radical Cation Facilitates the Deprotection of tert-Butyl Groups in Esters, Ethers, Carbonates, and Carbamates
Source: J Org Chem. 2023 May 1;88(11):6932–8. doi: 10.1021/acs.joc.3c00238 (PMC10242758; doi:10.1021/acs.joc.3c00238)
Supplement: Supplementary file 1 — jo3c00238_si_001.pdf [file jo3c00238_si_001.pdf]

SUPPORTING INFORMATION

Triarylamminium Radical Cation Facilitates the Deprotection of *tert*-Butyl Groups in Esters, Ethers, Carbonates and Carbamates

Denisa Hidasová and Tomáš Slanina*

Institute of Organic Chemistry and Biochemistry of the Czech Academy of Sciences, Flemingovo nám. 2, 166 10 Prague, Czech Republic

email: tomas.slanina@uochb.cas.cz

Table of Contents

1. General experimental information S3
2. Experimental synthetic procedures S3
   2.1. Starting materials S3
   2.2. General procedures S9
   2.3. Products S10
3. Control and mechanistic experiments S16
4. ^{1}H and ^{13}C NMR spectra S26
5. References S49

1. General experimental information

All reactions were performed under an argon atmosphere. Solvents and additives were dried prior to use according to standard procedures. TLC plates Silicagel 60 F254 (Merck KGaA) were used for monitoring reactions. Flash column chromatographic separations were performed on silica gel 60 (Fluka, 230-400 mesh). IR spectra were measured with a Bruker ALPHA FT-IR spectrometer as neat samples using an ATR device. ^{1}H and ^{13}C NMR spectra were recorded at room temperature on Bruker Avance 400 spectrometers equipped with a liquid nitrogen-cooled cryoprobe at 400.1 MHz for ^{1}H NMR and 100.6 MHz for ^{13}C NMR, respectively. The NMR shifts (δ) are shown in ppm. Connectivity was determined by ^{1}H-^{1}H COSY and HMBC experiments. ^{13}C NMR assignments were obtained from APT and HSQC experiments. ESI Mass spectra were obtained on a Thermo Fisher Scientific LCQ Fleet spectrometer, sample concentration approx. 1 µg/mL, spray voltage pos. mode: 3.3 kV. HRMS spectra were recorded on a Waters Q-Tof microspectrometer, resolution: 100000. Optical rotations were measured in the Microanalytical Laboratories of IOCB Prague. Melting points are uncorrected. Reaction yields were determined either as isolated yields or by the use of ^{1}H NMR spectroscopy (16 scans, relaxation time 1 s, acquisition time 5 s, repetition time 6 s). Internal standard (diphenylmethane) and authentic samples of products were used to determine the response factor of the NMR measurements. The molar amount of the starting material and products were calculated by the integral ratio of the characteristic signals of the respective compound and internal standard multiplied by their response factors.

2. Experimental synthetic procedures

2.1. Starting materials

(2*E*)-*tert*-butyl 3-(furan-2-yl)acrylate (**1d**),[1] 1-(3-phenylpropyl)-1-(1,1-dimethylethyl) ether (**3a**),[2] *tert*-butoxymethylbenzene (**3b**),[3] *tert*-butyl 3-phenylpropylcarbonate (**5**),[3] *tert*-butyl *N*-*p*-tolyl-*N*-methylcarbamate (**6**),[3] *tert*-butyl benzyl(methyl)carbamate (**8**),[3] succinic acid (ethyl)(*tert*-butyl)diester (**10**),[3] *p*-methoxybenzyl 3-phenylpropyl ether (**14**),[4] (3-

(methoxymethoxy)propyl)benzene,[5] were prepared according to literature procedures. Their spectral and physical data matched those reported.

Triethylsilane, tris-4-bromophenylamminium hexachloroantimonate (TBPA$^{\bullet+}$ $SbCl_6^-$, MB$^{\bullet+}$, Magic blue) and other reagents were purchased and used after the analytical control of their purity.

(*E*)-*tert*-Butyl 5-methylhex-2-enoate (**1c**):

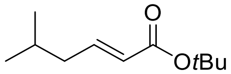

tert-Butyl (*E*)-5-methylhex-2-enoate was prepared from (*tert*-butoxycarbonylmethylene) triphenylphosphorane and 3-methylbutanal in analogy to a literature procedure.[1] Its analytical data matched those reported.[6] ^{1}H NMR (400 MHz, $CDCl_3$) δ 6.89-6.77 (m, 1H), 5.72 (dt, *J* = 15.5, 1.5 Hz, 1H), 2.05 (ddd, *J* = 7.5, 6.8, 1.5 Hz, 2H), 1.74 (dt, *J* = 13.3, 6.7 Hz, 1H), 1.47 (s, 9H), 0.91 (d, *J* = 6.7 Hz, 6H); ^{13}C{^{1}H} NMR (100 MHz, $CDCl_3$) δ 166.3, 147.1, 124.1, 80.1, 41.5, 28.3, 27.9, 22.5.

***tert*-Butyl (*S*)-2-(6-methoxynaphthalen-2-yl)propanoate** (**1e**):

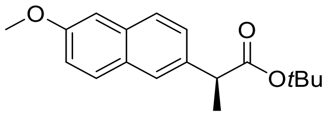

To a stirred solution of (2*S*)-2-(6-methoxy(2-naphthyl))propanoic acid (150 mg, 0.651 mmol) and *tert*-butanol (0.2 mL, 1.95 mmol) in 2 mL of dichloromethane, (4-dimethylamino)pyridine (6.5 mg, 0.0651 mmol) and subsequently *N*,*N′*-dicyclohexylcarbodiimide (162 mg, 0.781 mmol) were added at room temperature under an argon atmosphere. After 18 hours of stirring at room temperature, ethyl acetate was added and the organic phase was washed with brine, 1M HCl, 5% $NaHCO_3$ solution and brine, dried over $MgSO_4$ and evaporated *in vacuo*. The crude product was purified by column chromatography (hexane/EtOAc 50:1 gradient to 10:1) to afford 122 mg (66%) of colorless solid. Its analytical data matched those reported.[7] M.p. 80-81 °C; R_f = 0.5 (hexane/EtOAc = 10:1); $[\alpha]_D^{20}$ +10.1 (c 2.3, $CHCl_3$), Ref. $[\alpha]_D^{20}$ +21.3 (c 2.3, $CHCl_3$)[7]; ^{1}H NMR

(400 MHz, $CDCl_3$) δ 4.14 (q, *J* = 7.1 Hz, 2H), 2.60-2.49 (m, 4H), 1.44 (s, 9H), 1.26 (t, *J* = 7.1 Hz, 3H); $^{13}C\{^1H\}$ NMR (100 MHz, $CDCl_3$) δ 172.5, 171.5, 80.7, 60.6, 30.4, 29.4, 28.1, 14.2.

***N*-[(1,1-Dimethylethoxy)carbonyl]-L-alanine 1,1-dimethylethyl ester** (**12**):

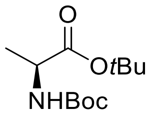

To a stirred solution of *N*-(*tert*-butoxycarbonyl)-L-alanine (190 mg, 1 mmol) and *tert*-butanol (0.3 mL, 3 mmol) in 2 mL of dichloromethane, (4-dimethylamino)pyridine (10 mg, 0.1 mmol) and subsequently *N,N′*-dicyclohexylcarbodiimide (250 mg, 1.2 mmol) were added at room temperature under an argon atmosphere. After 15 hours of stirring at room temperature, ethyl acetate was added and the organic phase was washed with brine, 1M HCl, 5% $NaHCO_3$ solution and brine, dried over $MgSO_4$ and evaporated *in vacuo*. The crude product was purified by column chromatography (hexane/EtOAc 50:1 gradient to 10:1) to afford 195 mg (80%) of colorless oil. Its analytical data matched those reported.[8] R_f = 0.4 (hexane/EtOAc = 10:1); $[\alpha]_D^{20}$ –8.6 (c 0.6, CH_2Cl_2), Ref. $[\alpha]_D^{26}$ –2.98 (c 0.6, CH_2Cl_2)[8]; ^1H NMR (400 MHz, $CDCl_3$) δ 5.05 (br s, 1H), 4.23-4.11 (m, 1H), 1.43 (s, 9H), 1.45 (s, 9H), 1.33 (d, *J* = 7.2 Hz, 3H); $^{13}C\{^1H\}$ NMR (100 MHz, $CDCl_3$) δ 172.7, 155.2, 81.8, 79.7, 49.9, 28.5, 28.1, 19.0.

3-Methylbut-2-en-1-yl (*S*)-2-(6-methoxynaphthalen-2-yl)propanoate

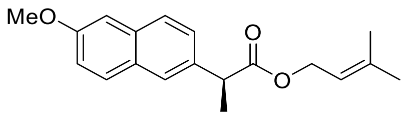

To a stirred solution of (2*S*)-2-(6-methoxy(2-naphthyl))propanoic acid (150 mg, 0.651 mmol) and 3-methylbut-2-en-1-ol (0.2 mL, 1.95 mmol) in 2 mL of dichloromethane (4-dimethylamino)pyridine (6.5 mg, 0.0651 mmol) and subsequently *N,N′*-dicyclohexylcarbodiimide (162 mg, 0.781 mmol) were added at room temperature under an argon atmosphere. After 18 hours of stirring at room temperature, ethyl acetate was added and the organic phase was washed with brine, 1M HCl, 5% $NaHCO_3$ solution and brine, dried over $MgSO_4$ and evaporated *in vacuo*. The crude product was purified by column chromatography (hexane/EtOAc 50:1 gradient to 10:1) to afford 158 mg (81%) of pale-yellow solid. M.p. 46-47 °C; R_f = 0.5 (hexane/EtOAc = 10:1); Ref.

$[\alpha]_D^{20}$ +23.2 (c 2.3, $CHCl_3$); ^{1}H NMR (400 MHz, $CDCl_3$) δ 7.73-7.63 (m, 3H), 7.41 (dd, *J* = 8.4, 1.9 Hz, 1H), 7.17-7.09 (m, 2H), 5.29 (tdt, *J* = 7.1, 2.8, 1.4 Hz, 1H), 4.66-4.57 (m, 1H), 4.56-4.48 (m, 1H), 3.91 (s, 3H), 3.85 (q, *J* = 7.2 Hz, 1H), 1.72 (s, 3H), 1.64 (s, 3H), 1.57 (d, *J* = 7.1 Hz, 3H); ^{13}C{^{1}H} NMR (100 MHz, $CDCl_3$) δ 174.9, 157.7, 139.1, 136.0, 133.8, 129.4, 129.1, 127.2, 126.4, 126.1, 119.1, 118.7, 105.7, 61.9, 55.5, 45.6, 25.9, 18.9, 18.2; HRMS (ESI+): m/z calcd for $C_{19}H_{23}O_3$ 299.1642 $[M+H]^+$, found 299.1641.

((3-Phenylpropoxy)methanetriyl)tribenzene:

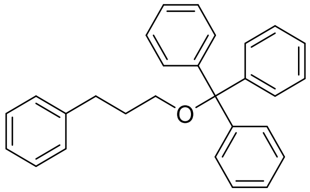

Trityl chloride (369 mg, 1.32 mmol) was added into solution of 3-phenylpropane-1-ol (100 mg, 0.734 mmol) in 1.5 mL of pyridine. After stirring for 24 hours the reaction mixture was diluted with dichloromethane and extracted with water. The organic layer was dried over $MgSO_4$ and evaporated *in vacuo* to afford 277 mg (99%) of colorless solid. Its analytical data matched those reported.[9] M.p. 82 °C; R_f = 0.8 (hexane/EtOAc = 10:1); ^{1}H NMR (400 MHz, $CDCl_3$) δ 7.50-7.41 (m, 6H), 7.34-7.19 (m, 11H), 7.19-7.10 (m, 3H), 3.12 (t, *J* = 6.2 Hz, 2H), 2.78-2.68 (m, 2H), 1.99-1.88 (m, 2H); ^{13}C{^{1}H} NMR (100 MHz, $CDCl_3$) δ 144.6, 142.3, 128.9, 128.6, 128.4, 127.9, 127.0, 125.8, 86.5, 62.9, 32.7, 31.9; HRMS (EI+): m/z calcd for $C_{28}H_{26}O$ 378.1978 $[M+H]^+$, found 378.1980.

2-(3-Phenylpropoxy)tetrahydro-2*H*-pyran:

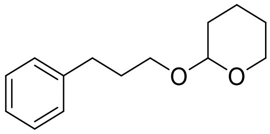

A solution of 3-phenylpropane-1-ol (200 mg, 1.47 mmol) and dihydropyrane (141 µL, 1.54 mmol) in the presence of *p*-toluenesulfonic acid (14 mg, 0.0735 mmol) was stirred for 24 hours. Then the

solution was poured on saturated aqueous $NaHCO_3$ sol. and extracted with Et_2O. The combined organic layers were dried over $MgSO_4$, filtered and concentrated *in vacuo*. The crude product was purified by column chromatography (hexane/EtOAc 10:1) to afford 260 mg (80%) of colorless oil. Its analytical data matched those reported.[10] R_f = 0.5 (hexane/EtOAc = 10:1); ^{1}H NMR (400 MHz, $CDCl_3$) δ 7.31-7.26 (m, 2H), 7.23-7.14 (m, 3H), 4.58 (dd, *J* = 4.5, 2.8 Hz, 1H), 3.94-3.83 (m, 1H), 3.78 (dt, *J* = 9.7, 6.5 Hz, 1H), 3.55-3.46 (m, 1H), 3.42 (dt, *J* = 9.7, 6.5 Hz, 1H), 2.80-2.63 (m, 2H), 1.93 (tt, *J* = 7.8, 6.5 Hz, 2H), 1.88-1.79 (m, 1H), 1.79-1.67 (m, 1H), 1.66-1.46 (m, 4H); ^{13}C{^{1}H} NMR (100 MHz, $CDCl_3$) δ 142.2, 128.6, 128.5, 125.9, 99.1, 67.0, 62.5, 32.6, 31.5, 30.9, 25.7, 19.8; HRMS (EI+): m/z calcd for $C_{14}H_{20}O_2$ 220.1458 $[M+H]^+$, found 220.1455.

***O,O'*-((5-(((3-(*tert*-butoxy)-3-oxopropanoyl)oxy)methyl)-1,3-phenylene)bis(methylene)) di-*tert*-butyl disuccinate (18)**:

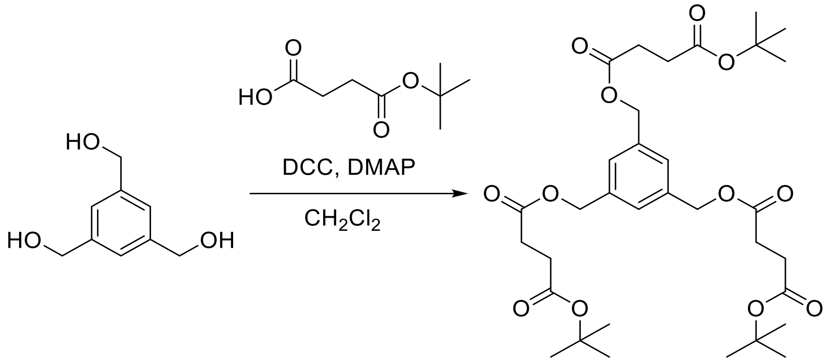

To a stirred solution of 4-(*tert*-butoxy)-4-oxobutanoic acid (311 mg, 1.78 mmol) and benzene-1,3,5-triyltrimethanol (100 mg, 0.594 mmol) in 4 mL of dichloromethane, (4-dimethylamino)pyridine (24.2 mg, 0.178 mmol) and subsequently *N*,*N'*-dicyclohexylcarbodiimide (368 mg, 1.78 mmol) were added at room temperature under an argon atmosphere. After 18 hours of stirring at room temperature ethyl acetate was added and the organic phase was washed with brine, 1M HCl, 5% $NaHCO_3$ solution and brine, dried over $MgSO_4$ and evaporated *in vacuo*. The crude product was purified by column chromatography (hexane/EtOAc 10:1) to afford 277 mg (72%) of colorless oil. R_f = 0.5 (hexane/EtOAc = 10:1); IR (neat) ν 2978, 2932, 1726, 1488, 1452, 1411, 1390, 1366, 1318, 1249, 1140, 1027, 996, 965, 891, 847, 759 cm^{-1}; ^{1}H NMR (400 MHz, $CDCl_3$) δ 7.29 (s, 3H), 5.13 (s, 6H), 2.64 (ddd, *J* = 7.1, 6.0, 1.5 Hz, 6H), 2.56 (ddd, *J* = 7.8, 6.0,

1.4 Hz, 6H), 1.43 (s, 27H); $^{13}C\{^1H\}$ NMR (100 MHz, $CDCl_3$) δ 172.4, 171.5, 136.9, 127.8, 80.9, 66.0, 30.4, 29.5, 28.2; HRMS (ESI+): m/z calcd for $C_{33}H_{48}O_{12}Na$ 659.3038 $[M+Na]^+$, found 659.3037.

(*S*)-5-(*N*-(2-(2-((((9*H*-fluoren-9-yl)methoxy)carbonyl)amino)-5-(*tert*-butoxy)-5-oxopentan amido)ethyl)sulfamoyl)-2-(6-(diethylamino)-3-(diethyliminio)-3*H*-xanthen-9-yl)benzenesulfonate (**16**):

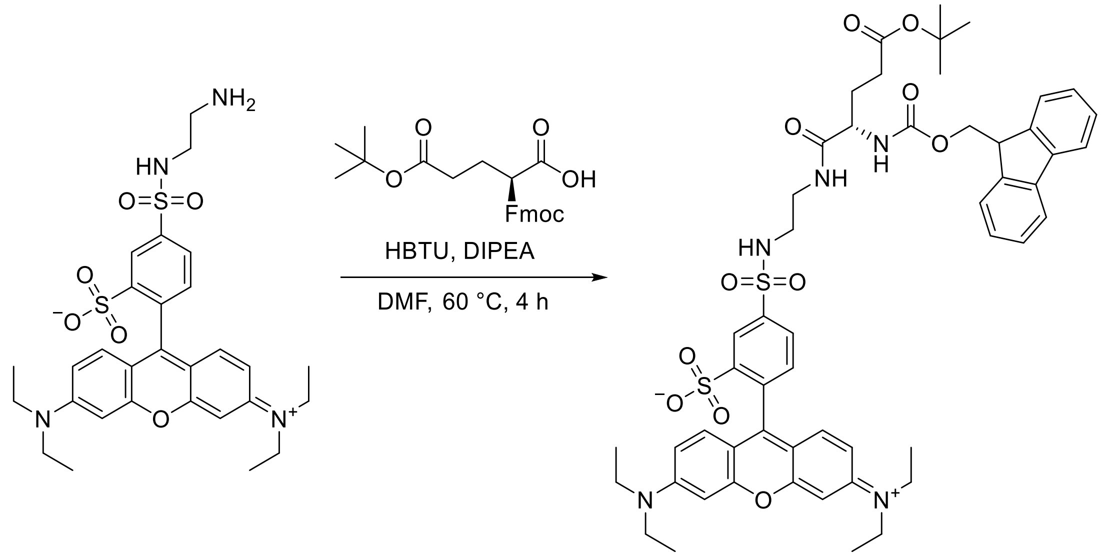

(*S*)-2-(((9*H*-fluoren-9-yl)methoxy)carbonyl)-5-(*tert*-butoxy)-5-oxopentanoic acid (160 mg, 0.375 mmol), HBTU (142 mg, 0.375 mmol) and DIPEA (65 µL, 0.375 mmol) were dissolved in 2.5 mL DMF and solution of 5-(*N*-(2-aminoethyl)sulfamoyl)-2-(6-(diethylamino)-3-(diethyliminio)-3*H*-xanthen-9-yl)benzenesulfonate (150 mg, 0.250 mmol) in 1 mL DMF was added. The reaction mixture was heated for 4 hours at 60 °C under nitrogen atmosphere. Then the reaction mixture was quenched with H_2O and extracted with EtOAc. The combined organic layers were washed with sat. $NaHCO_3$ sol., H_2O, brine, dried over $MgSO_4$ and evaporated *in vacuo*. The crude product was purified by column chromatography (CH_2Cl_2/MeOH 50:1 gradient to 10:1) to afford 145 mg (53%) of red solid. M.p. 170 °C; R_f = 0.4 (CH_2Cl_2/MeOH = 10:1); ^1H NMR (400 MHz, MeOD) δ 8.68 (d, *J* = 1.9 Hz, 1H), 8.10 (dd, *J* = 8.0, 2.0 Hz, 1H), 7.82 (d, *J* = 7.5 Hz, 1H), 7.79-7.61 (m, 5H), 7.45-7.24 (m, 7H), 7.08 (dd, *J* = 9.4, 2.2 Hz, 2H), 6.93 (ddd, *J* = 20.3, 8.9, 2.4 Hz, 4H), 4.36 (dd, *J* = 10.5, 7.0 Hz, 3H), 4.28-4.14 (m, 2H), 4.14-4.06 (m, 1H), 3.69-3.63 (m, 8H), 3.45-3.28 (m, 6H), 3.16 (t, *J* = 6.3 Hz, 1H), 2.36 (t, *J* = 7.8 Hz, 1H), 2.15-2.06 (m, 1H), 1.96-1.85 (m, 1H), 1.47

(s, 9H), 1.31 (t, *J* = 7.1 Hz, 12H); $^{13}C\{^{1}H\}$ NMR (100 MHz, MeOD) δ 174.7, 173.9, 159.3, 159.2, 158.4, 157.6, 157.1, 157.0, 147.2, 145.3, 145.1, 143.6, 142.5, 135.4, 133.6, 132.6, 129.3, 128.8, 128.2, 127.7, 126.3, 120.9, 115.19, 115.17, 115.0, 97.0, 81.8, 68.1, 55.8, 46.8, 43.8, 32.7, 28.4, 18.7, 17.3, 13.1, 12.9; HRMS (ESI+): m/z calcd for $C_{53}H_{61}N_5O_{11}S_2Na$ 1030.3701 $[M+Na]^+$, found 1030.3700.

2.2. General procedures

General procedure for deprotection of *tert*-butyl groups in esters, ethers, carbonates, and carbamates:

To a solution of *tert*-butylated compound (0.34 mmol, 1.0 equiv) in dry dichloromethane (3 mL), triethylsilane (0.68 mmol, 2.0 equiv) was added, followed by tris-4-bromophenylamminium cation radical (0.17 mmol, 0.5 equiv). The resulting mixture was stirred at room temperature until the starting material was consumed as indicated by TLC. The mixture was evaporated, and the residue was purified by column chromatography, which gave the desired product.

General procedure for deprotection of *tert*-butyl groups in *tert*-butyl-acrylate by tributyltin hydride:

To a solution of *tert*-butyl ester (0.34 mmol, 1.0 equiv) in the mixture of dry dichloromethane and dry tetrahydrofurane (6 mL, ratio of the mixture 1:1, deoxygenated solvents), tris-4-bromophenylamminium cation radical (0.17 mmol, 0.5 equiv) was added, followed by tributyltin hydride (0.34 mmol, 1.0 equiv). The resulting mixture was stirred at room temperature for 5 minutes. The mixture was evaporated, and the residue was purified by column chromatography, which gave the desired carboxylic acid.

General procedure for deprotection of *p*-methoxybenzyl ethers:

To a solution of *p*-methoxybenzyl ether (0.34 mmol, 1.0 equiv) in dry dichloromethane (3 mL), tris-4-bromophenylamminium cation radical (0.17 mmol, 0.5 equiv) was added. The resulting mixture was stirred at room temperature for 15 minutes. The mixture was evaporated, and the residue was purified by column chromatography, which gave the desired alcohol.

General procedure for *trans*-esterification:

To a solution of *tert*-butylated compound (0.34 mmol, 1 equiv) in dry dichloromethane (3 mL), triethylsilane (0.68 mmol, 2 equiv) was added, followed by tris-4-bromophenylamminium cation radical (0.17 mmol, 0.5 equiv). The resulting mixture was stirred at room temperature until the starting material was consumed as indicated by TLC and dry methanol (3 mL) was added. The mixture was stirred for 16 hours, evaporated, and the residue was purified by column chromatography, which gave the desired product.

2.3. Products

Acrylic acid (2a):

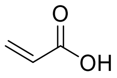

Yield: 23.3 mg (95%). The compound was identified by spectral comparison with the sample purchased from a commercial source.[11] Colorless solid. R_f = 0.2 (hexane/AcOEt = 1:1, silica gel plate); ^{1}H NMR (400 MHz, $CDCl_3$) δ 6.53 (dd, *J* = 17.2, 1.3 Hz, 1H), 6.15 (dd, *J* = 17.3, 10.4 Hz, 1H), 5.98 (dd, *J* = 10.5, 1.4 Hz, 2H); $^{13}C\{^1H\}$ NMR (100 MHz, $CDCl_3$) δ 171.3, 146.2, 128.0.

(*E*)-but-2-enoic acid (2b):

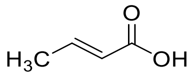

Yield: 29.3 mg (95%). The compound was identified by spectral comparison with the sample purchased from a commercial source.[12] Colorless solid. M.p. 71 °C; R_f = 0.3 (hexane/AcOEt = 1:1, silica gel plate); ^{1}H NMR (400 MHz, $CDCl_3$) δ 7.09 (dq, *J* = 15.5, 6.9 Hz, 1H), 5.86 (dq, *J* =

15.5, 1.7 Hz, 1H), 1.92 (dd, *J* = 6.9, 1.7 Hz, 3H); $^{13}C\{^{1}H\}$ NMR (100 MHz, $CDCl_3$) δ 172.0, 147.6, 122.3, 18.3.

(*E*)-5-methylhex-2-enoic acid (2c):

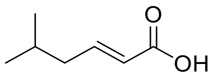

Yield: 40 mg (92%). Their spectral and physical data matched those reported.[13] Colorless liquid. R_f = 0.6 (hexane/AcOEt = 1:1, silica gel plate); ^{1}H NMR (400 MHz, $CDCl_3$) δ 7.07 (dt, *J* = 15.3, 7.5 Hz, 1H), 5.82 (dt, *J* = 15.6, 1.4 Hz, 1H), 2.13 (ddd, *J* = 7.5, 6.7, 1.5 Hz, 2H), 0.94 (d, *J* = 6.7 Hz, 6H); $^{13}C\{^{1}H\}$ NMR (100 MHz, $CDCl_3$) δ 171.9, 151.5, 121.7, 41.7, 27.4, 22.5.

3-(fur-2-yl)crotonic acid (2d):

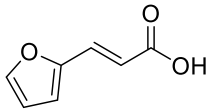

Yield: 37.6 mg (80%). The compound was identified by spectral comparison with the sample purchased from a commercial source.[14] Colorless solid. M.p. 139-140 °C; R_f = 0.2 (hexane/AcOEt = 1:1, silica gel plate); ^{1}H NMR (400 MHz, $CDCl_3$) δ 7.56-7.48 (m, 2H), 6.67 (dt, *J* = 3.4, 0.6 Hz, 1H), 6.49 (dd, *J* = 3.4, 1.8 Hz, 1H), 6.32 (dt, *J* = 15.7, 0.5 Hz, 1H); $^{13}C\{^{1}H\}$ NMR (100 MHz, $CDCl_3$) δ 171.9, 150.8, 145.4, 133.2, 115.9, 114.9, 112.6.

(2*S*)-2-(6-methoxy(2-naphthyl))propanoic acid (2e):

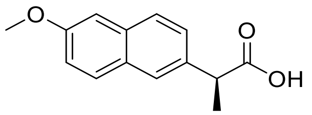

Yield: 54.8 mg (70%). The compound was identified by spectral comparison with the sample purchased from a commercial source.[15] Colorless solid. M.p. 152 °C; R_f = 0.2 (hexane/AcOEt = 1:1, silica gel plate); $[\alpha]_D^{20}$ +35.1 (c 0.3, $CHCl_3$), Ref. $[\alpha]_D^{20}$ +65.7 (c 0.3, $CHCl_3$); ^{1}H NMR (400 MHz, $CDCl_3$) δ 7.73-7.66 (m, 3H), 7.41 (dd, *J* = 8.5, 1.9 Hz, 1H), 7.17-7.07 (m, 2H), 3.91 (s, 3H), 3.88 (q, *J* = 7.1 Hz, 1H), 1.59 (d, *J* = 7.1, 3H); $^{13}C\{^{1}H\}$ NMR (100 MHz, $CDCl_3$) δ 180.0, 157.9, 135.0, 134.0, 129.5, 129.1, 127.4, 126.33, 126.29, 119.20, 105.8, 55.5, 45.3, 18.3.

3-Phenyl-1-propanol (4a):

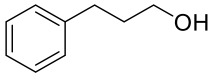

Yield: 44 mg (95%). The compound was identified by spectral comparison with the sample purchased from a commercial source.[3] Colorless liquid. R_f = 0.5 (hexane/AcOEt = 1:1, silica gel plate); ^{1}H NMR (400 MHz, $CDCl_3$) δ 7.35-7.14 (m, 2H), 3.68 (t, J = 6.4 Hz, 2H), 2.79-2.65 (m, 2H), 1.99-1.82 (m, 2H), 1.46 (s, 1H); ^{13}C{^{1}H} NMR (100 MHz, $CDCl_3$) δ 142.0, 128.6, 128.5, 126.0, 62.4, 34.4, 32.2.

Benzyl alcohol (4b):

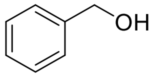

Yield: 31 mg (84%). The compound was identified by spectral comparison with the sample purchased from a commercial source.[16] Colorless liquid. R_f = 0.6 (hexane/AcOEt = 1:1, silica gel plate); ^{1}H NMR (400 MHz, $CDCl_3$) δ 7.39-7.27 (m, 5H), 4.70 (s, 2H); ^{13}C{^{1}H} NMR (100 MHz, $CDCl_3$) δ 141.0, 128.7, 127.8, 127.1, 65.6.

***N*-methyl-*p*-toluidine (7):**

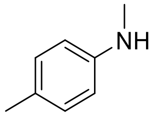

Yield: 39.1 mg (95%). Its spectral and physical data matched those reported.[3] Light-yellow liquid; R_f = 0.36 (hexane/AcOEt = 6:1, silica gel plate); ^{1}H NMR (400 MHz, $CDCl_3$) δ 7.02 (d, J = 8.2 Hz, 2H), 6.57 (d, J = 8.2 Hz, 2H), 3.55 (bs, 1H), 2.83 (s 3H), 2.26 (s, 3H); ^{13}C{^{1}H} NMR (100 MHz, $CDCl_3$) δ 147.2, 129.7, 126.4, 112.6, 31.1, 20.4.

N-Methyl-1-phenylmethanamine (9):

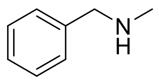

Yield: 39.1 mg (95%). Its spectral and physical data matched those reported.[3] Light-yellow oil. R_f = 0.7 (hexane/EtOAc = 1:1, silica gel plate); ^{1}H NMR (400 MHz, $CDCl_3$) δ 7.57-7.48 (m, 2H), 7.43-7.32 (m, 3H), 4.05 (s, 2H), 2.52 (s, 3H); ^{13}C{^{1}H} NMR (100 MHz, $CDCl_3$) δ 130.3, 129.7, 129.4, 52.5, 29.9.

Butanedioic acid, monoethyl ester (11):

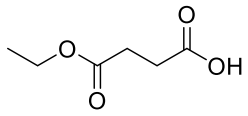

Yield: 47.2 mg (95%). Its spectral and physical data matched those reported.[3] Colorless liquid. R_f = 0.2 (hexane/AcOEt = 1:1, silica gel plate); ^{1}H NMR (400 MHz, $CDCl_3$) δ 4.16 (q, J = 7.1 Hz, 2H), 2.73-2.65 (m, 1H), 2.65-2.57 (m, 1H), 1.26 (t, J = 7.1 Hz, 3H); ^{13}C{^{1}H} NMR (100 MHz, $CDCl_3$) δ 178.1, 172.3, 61.0, 29.1, 14.3.

***tert*-butyl L-alaninate (13):**

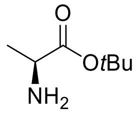

Yield: 63.7 mg (99%). The compound was identified by spectral comparison with the sample purchased from a commercial source.[17] Colorless solid. M.p. 148 °C; R_f = 0.2 (hexane/AcOEt = 1:1, silica gel plate); ^{1}H NMR (400 MHz, CD_3CN) δ 8.22 (br s, 2H), 3.94 (q, J = 7.2 Hz, 1H), 1.55 (d, J = 7.2 Hz, 3H), 1.48 (s, 9H); ^{13}C{^{1}H} NMR (100 MHz, CD_3CN) δ 169.8, 84.5, 50.5, 28.0, 16.3.

Methyl (*E*)-5-methylhex-2-enoate (15):

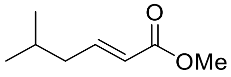

Yield: 45.9 mg (95%). Its spectral and physical data matched those reported.[18] Colorless liquid; R_f = 0.5 (hexane/EtOAc = 10:1, silica gel plate); ^{1}H NMR (400 MHz, $CDCl_3$) δ 6.95 (dt, *J* = 15.5, 7.5 Hz, 1H), 5.81 (dt, *J* = 15.6, 1.5 Hz, 1H), 3.72 (s, 3H), 2.09 (ddd, *J* = 7.5, 6.8, 1.5 Hz, 2H), 1.76 (dp, *J* = 13.4, 6.7 Hz, 1H), 0.92 (d, *J* = 6.7 Hz, 6H); ^{13}C{^{1}H} NMR (100 MHz, $CDCl_3$) δ 167.2, 148.7, 122.0, 51.5, 41.6, 27.9, 22.5.

(*S*)-5-(*N*-(2-(2-((((9*H*-fluoren-9-yl)methoxy)carbonyl)amino)-4-carboxybutanamido)ethyl)-sulfamoyl)-2-(6-(diethylamino)-3-(diethyliminio)-3*H*-xanthen-9-yl)benzenesulfonate (17):

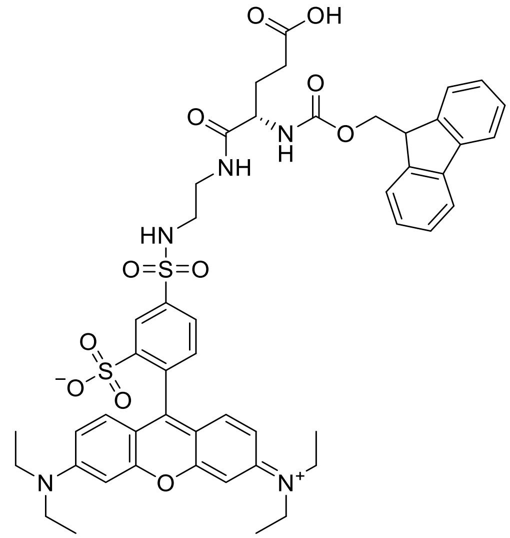

Yield: 61.7 mg (95%).[a] Red solid; M.p. >300 °C; R_f = 0.4 (CH_2Cl_2/MeOH = 10:1, silica gel plate); ^{1}H NMR (400 MHz, MeOD) δ 8.66 (d, *J* = 1.9 Hz, 1H), 8.10 (d, *J* = 7.9 Hz, 1H), 7.79-7.69 (m, 2H), 7.64 (t, *J* = 6.5 Hz, 2H), 7.40-7.31 (m, 3H), 7.31-7.22 (m, 2H), 7.07 (dd, *J* = 9.5, 3.1 Hz, 2H), 7.00-6.86 (m, 4H), 4.32 (t, *J* = 6.6 Hz, 2H), 4.23-4.14 (m, 1H), 4.14-4.05 (m, 1H), 3.78-3.54 (m, 8H), 3.45-3.28 (m, 6H), 3.16 (t, *J* = 6.3 Hz, 2H), 2.36 (t, *J* = 7.3 Hz, 2H), 2.15-2.04 (m, 1H), 2.00-1.86 (m, 1H), 1.31 (t, *J* = 7.0 Hz, 12H); ^{13}C{^{1}H} NMR (125.7 MHz, MeOD) δ 184.8, 173.7, 157.92, 157.90, 157.2, 156.2, 155.73, 155.70, 145.7, 143.9, 143.7, 142.3, 141.1, 134.1, 132.24, 132.21, 131.3, 128.0, 127.4, 126.8, 126.3, 124.92, 124.88, 119.5, 113.82, 113.80, 113.6, 95.6, 66.8, 55.0,

46.9, 45.4, 41.9, 39.0, 11.5; HRMS (ESI+): m/z calcd for $C_{49}H_{54}O_{11}N_5S_2$ 952.3256 $[M+H]^+$, found 952.3254.

[a] Done on 0.06826 mmol scale.

4,4',4''-((benzene-1,3,5-triyltris(methylene))tris(oxy))tris(4-oxobutanoic acid) (**19**):

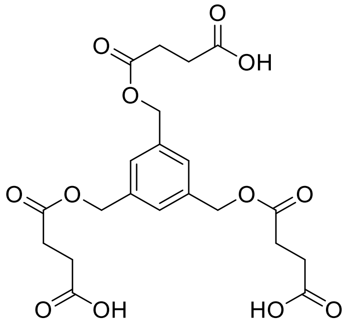

Yield: 157.7 mg (99%). Colorless solid; M.p. 131-132 °C; R_f = 0 (EtOAc, silica gel plate); IR (neat) ν 3700-3000 (br), 2930, 1731, 1712, 1409, 1387, 1352, 1208, 1161, 997, 959, 865 cm^{-1}; ^1H NMR (400 MHz, CD_3CN) δ 7.30 (s, 3H), 5.11 (s, 6H), 2.65-2.56 (m, 12H); $^{13}C\{^1H\}$ NMR (100 MHz, CD_3CN) δ 174.3, 173.1, 138.2, 127.8, 66.4, 29.7, 29.2; HRMS (ESI–): m/z calcd for $C_{21}H_{23}O_{12}$ 467.1190 $[M–H]^+$, found 467.1195.

3. Control and mechanistic experiments

Scheme S1. Control experiment: a) de-*tert*-butylation with tris-4-bromophenylamminium hexafluoroantimonate and b) de-*tert*-butylation with tetrabutylammonium hexafluorophosphate

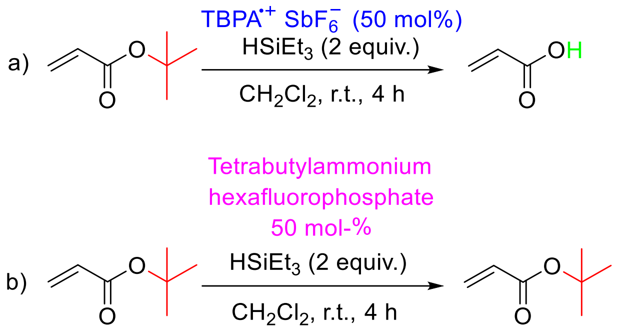

Figure S1: UV-VIS absorption spectra demonstrating a gradual reduction of Magic blue in the presence of Et_3SiH in CH_2Cl_2 (c = 1×10^{-4} M, CH_2Cl_2, in dark) accompanied with decoloration (decrease of absorbance >400 nm).

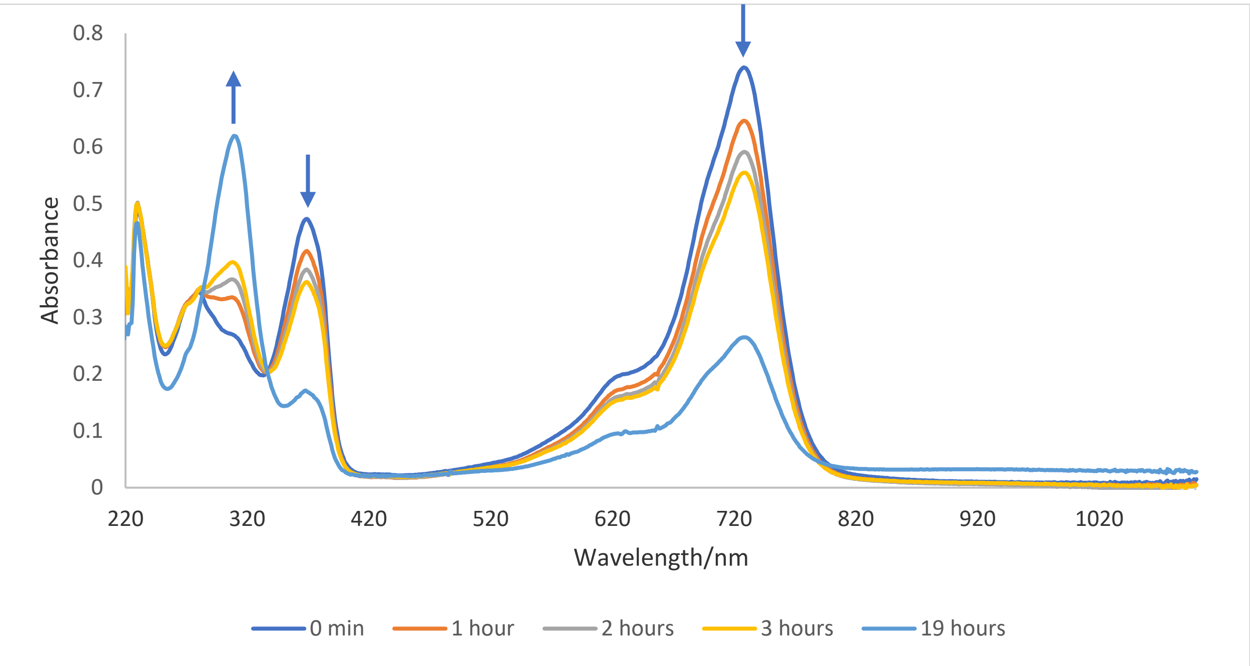

Figure S2: Graph of reduction of Magic blue in CH_2Cl_2 (c = 1×10^{-4} M, room temperature, dark) following the normalized absorbance at 720 nm.

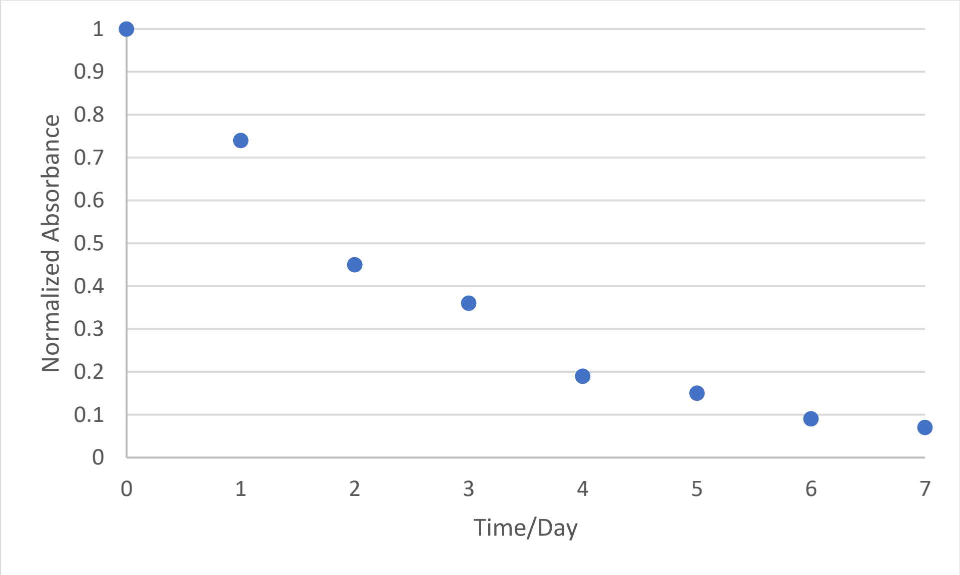

Figure S3: Kinetics of de-*tert*-butylation of *tert*-butyl acrylate**.** The conversion of **1a** and the yield of the product **2a** (blue line) were determined by ^{1}H NMR specroscopy of the crude materials.

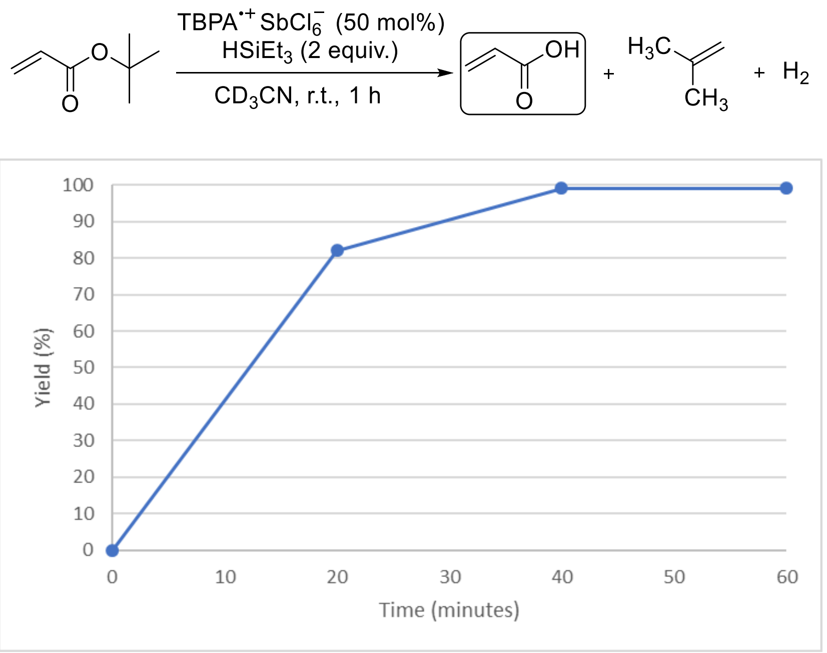

Figure S4: Comparison of the kinetics of ester (de-*tert*-butylation of *tert*-butyl acrylate **1a**, grey line), carbonate (*tert*-butyl (3-phenylpropyl) carbonate **5**, orange line), and ether ((3-(*tert*-butoxy)propyl)benzene **3a**, blue line) as determined by ^{1}H NMR spectra of the crude materials.

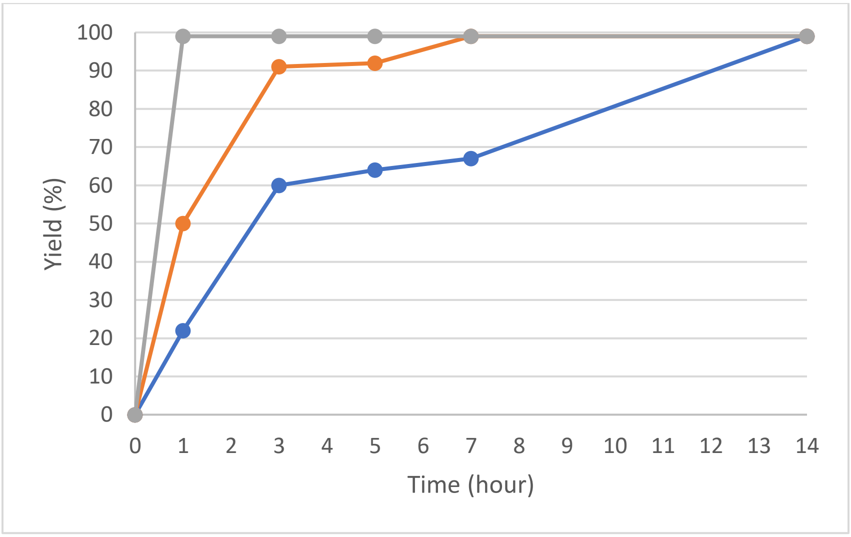

Figure S5: ^{1}H-NMR spectrum of the crude reaction mixture of *tert*-butyl acrylate **1a** with tris-4-bromophenylamminium cation radical/Et_3SiH in CD_3CN (*c* = 0.1 mM) after 1 h at room temperature.

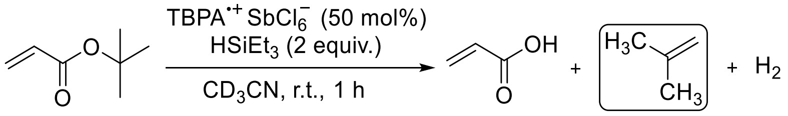

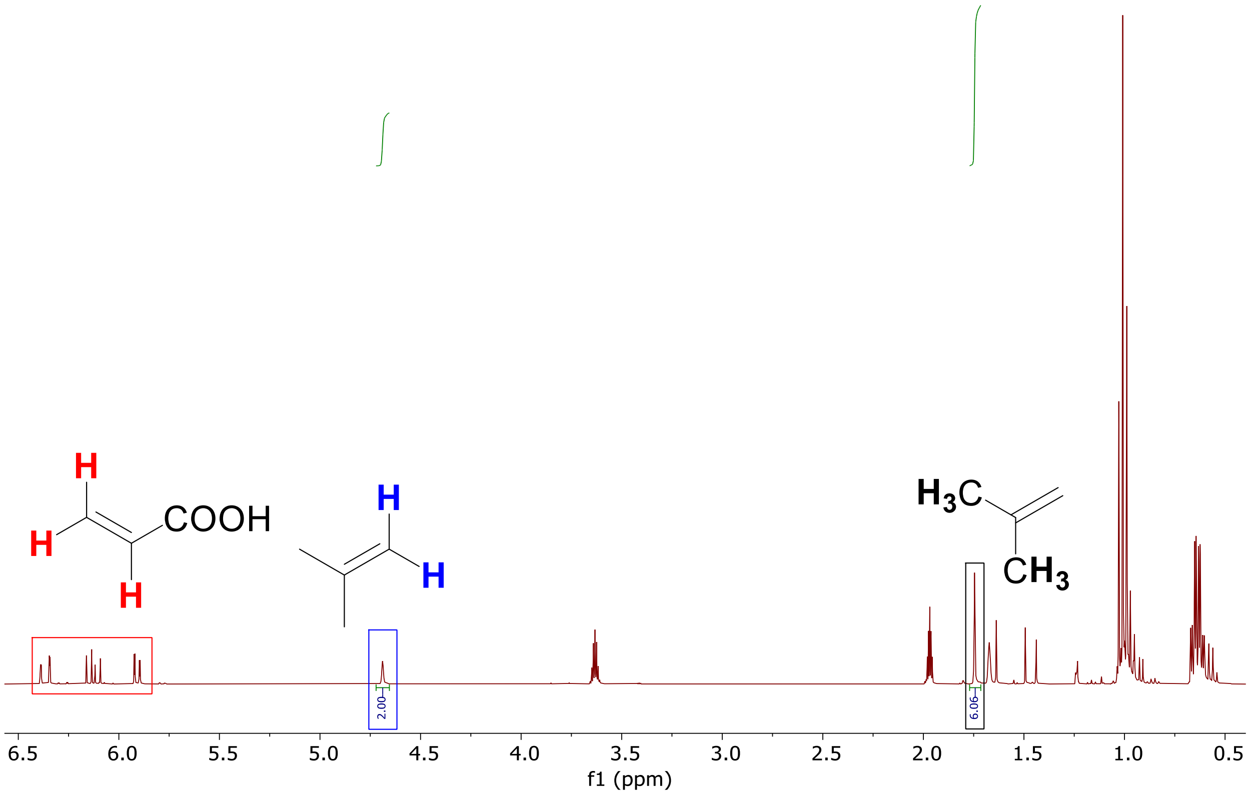

Figure S6: ^{1}H-NMR spectrum of the crude reaction mixture of *tert*-butyl acrylate **1a** with tris-4-bromophenylamminium cation radical (50 mol%)/Et$_3$SiH (2 equiv.) with internal standard diphenylmethane (1 equiv.) for better quantification. Tris-4-bromophenylamminium cation radical was reduced by long standing in the solvent (4 h).

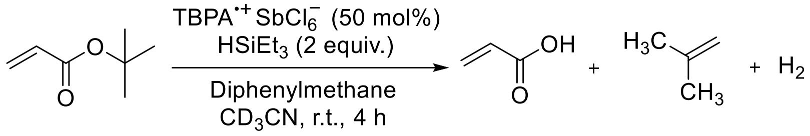

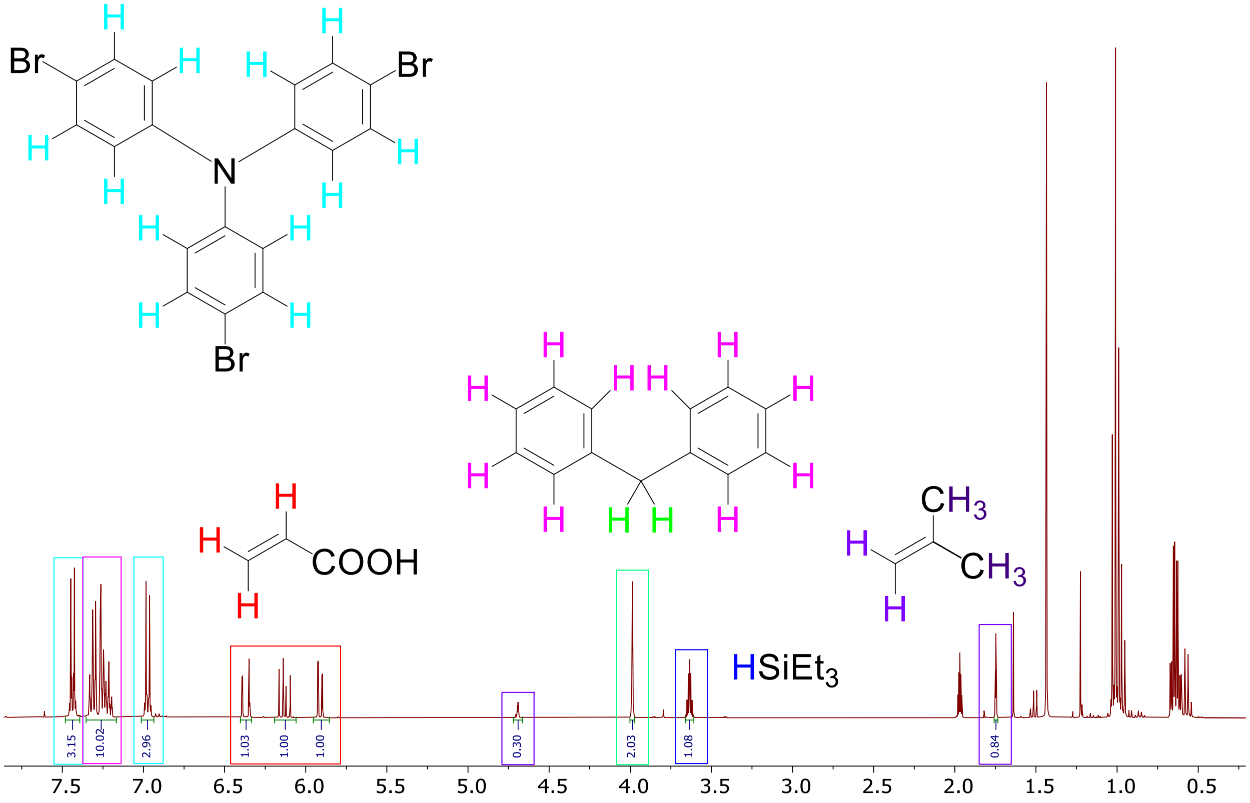

Figure S7: ^{1}H-NMR spectrum of the crude reaction mixture of *tert*-butyl acrylate **1a** with tris-4-bromophenylamminium cation radical (50 mol%)/Et_3SiH (50 mol%): after $HSiEt_3$ was consumed (no signal at 3.63 ppm), Pd(C) was added which partially converted acrylic acid (red signals) to propionic acid (signals in green). Tris-4-bromophenylamminium cation radical was reduced by long standing in the solvent.

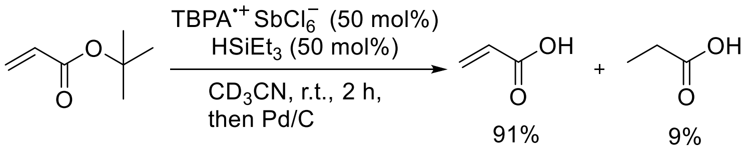

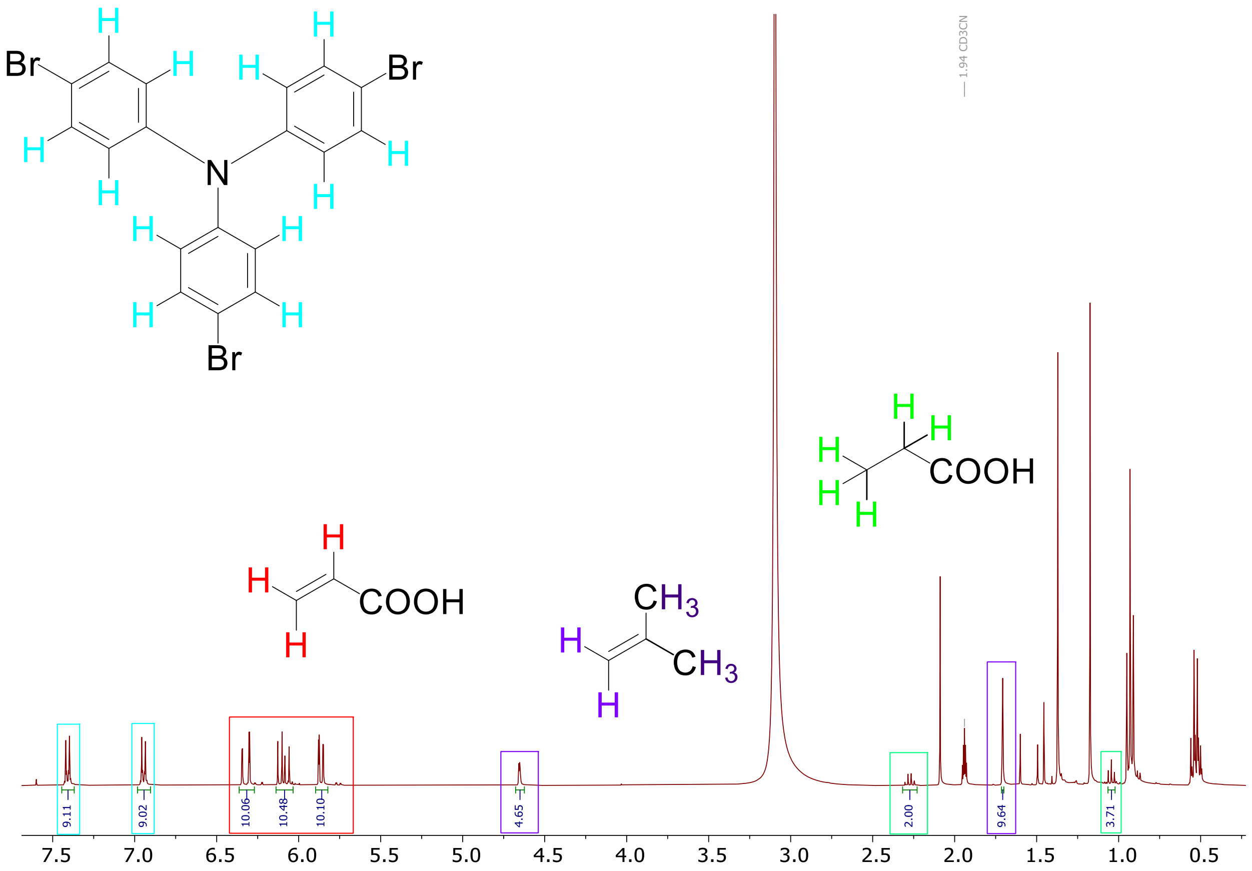

Figure S8: Superimposed spectra of *tert*-butyl acrylate **1a** (bottom) and its equimolar mixture with tris-4-bromophenylamminium cation radical $MB^{•+}$ in CD_3CN (top, c = 0.1 mM)), and acrylic acid **2a** (middle), zoomed regions of double bond protons (δ = 5-7.5 ppm) and solvent residual peak (δ = 2.0 ppm). The intensity of individual spectra is normalized to the solvent residual peak. The missing intensity of acrylate double bond signals corresponds to the formation of a complex of **1a** and **2a** with paramagnetic $MB^{•+}$.

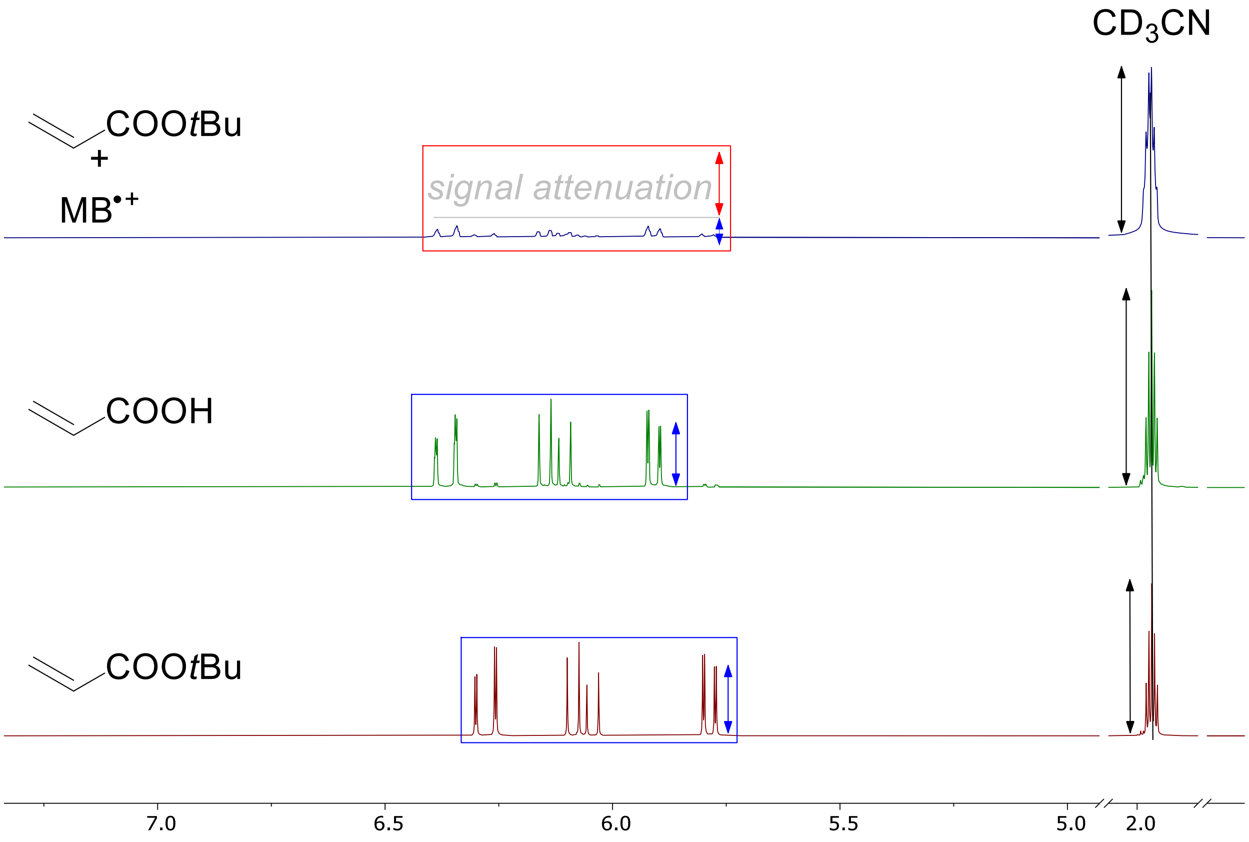

Table S1: Control experiments for catalytic de-*tert*-butylation. The reactions were performed using **1a** (0.06826 mmol), $MB^{\bullet+}$, $HSiEt_3$ and the solvent (0.5 mL) at room temperature.

Entry	**$MB^{\bullet+}$** (mol-%)	$HSiEt_3$ (equiv.)	Solvent	Yield of acid (%)[a]
1	50	0	CH_2Cl_2	0
2	50	0	CH_3CN	44
3	50	2[b]	CH_3CN	99

[a] Determined by ^{1}H NMR analysis of the crude mixture.
[b] Dimethylphenylsilane used instead of $HSiEt_3$

Table S2: Optimization of de-*tert* butylation of **1a** mediated by Magic blue and tributyltin hydride.

Entry	**MB** (mol-%)	Bu_3SnH (equiv.)	Solvent	Yield of acid (%)[a]
1	0	1	CH_2Cl_2	0
2	50	1	CH_2Cl_2	32
3	50	1	CH_3CN/THF	67
4	50	1	CH_2Cl_2/THF	74
5	100	1	CH_2Cl_2/THF	99

[a] Determined by ^{1}H NMR analysis after 5 minutes.

Table S3: Magic blue-catalyzed reaction of various compounds. The reaction was carried out using the substrate (0.06826 mmol), Magic blue (0.03413 mmol, 50 mol-%), Et_3SiH (0.1365 mmol, 2 equiv.) in CH_2Cl_2 (0.5 mL) at room temperature for 4 h.

Entry	Substrate	Yield of recovered starting material[a]
1	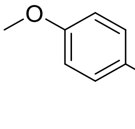	trace (decomposed)
2	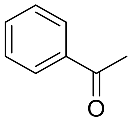	100%
3	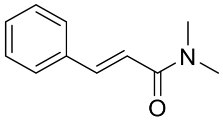	100%
4	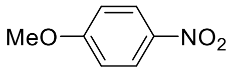	100%
5	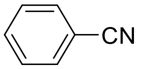	100%

[a] Determined by ^{1}H NMR analysis.

Table S4. Magic blue-catalyzed reaction of various compounds. The reaction was carried out using a substrate (0.06826 mmol), Magic blue (0.03413 mmol, 50 mol-%), Et_3SiH (0.1365 mmol, 2 equiv.) in CH_2Cl_2 (0.5 mL) at room temperature for 4 h.

Entry	Substrate	Product	Yield [a]
1	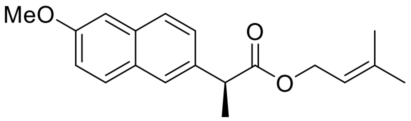	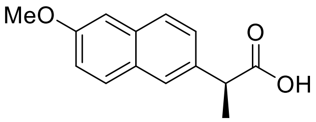	76%
2	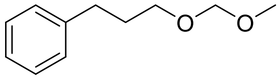	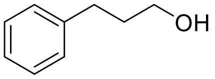	99%
3	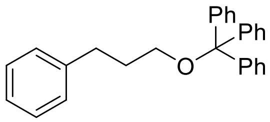	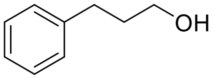	99%
4	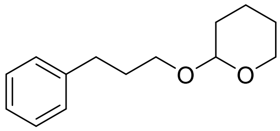	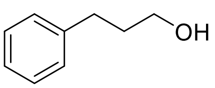	99%

[a] Isolated yield.

Table S5. Comparison of Magic blue/Et_3SiH and TFA methods for de-*tert* butylation of *tert*-butyl ester **1a**, *tert*-butyl ether **3a**, and *O*-Boc derivative **5**.

Entry	Substrate	Time	Magic blue/Et_3SiH [a]	TFA (2 equiv) [a]
1	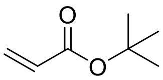	1 h	99%	8%
2	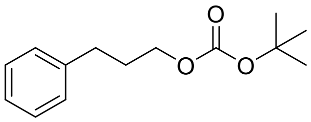	7 h	99%	0%
3	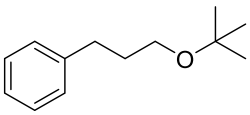	14 h	99%	0%

[a] Isolated yield.

4. ^{1}H and ^{13}C NMR spectra

Figure S9: Comparison of ^{1}H-NMR spectra of *tert*-butylated rhodamine derivative **16** and its de-*tert*-butylated analogue **17**.

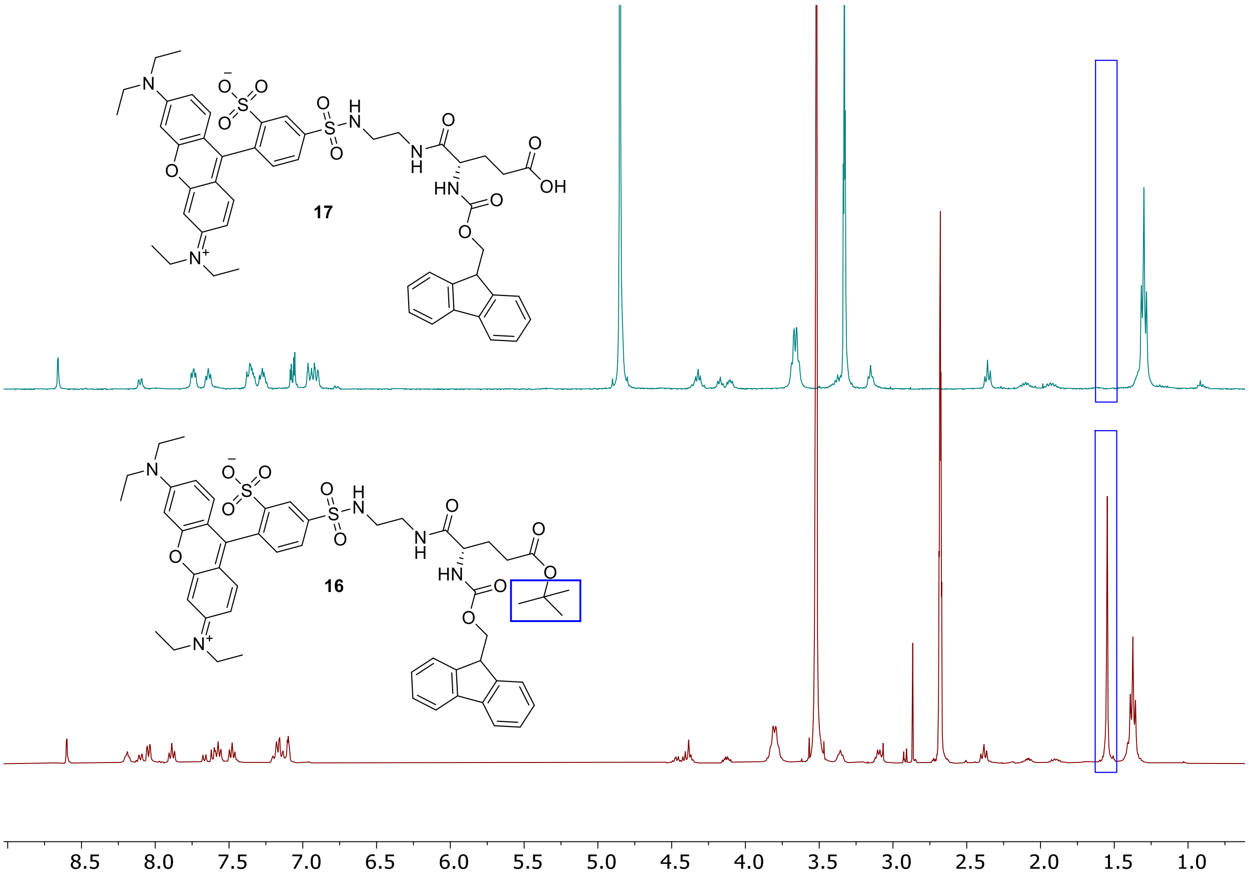

Figure S10: ^{1}H (400 MHz) and ^{13}C{^{1}H} (100 MHz) NMR spectra of (*E*)-*tert*-butyl 5-methyl-hex-2-enoate (**1c**) in $CDCl_3$.

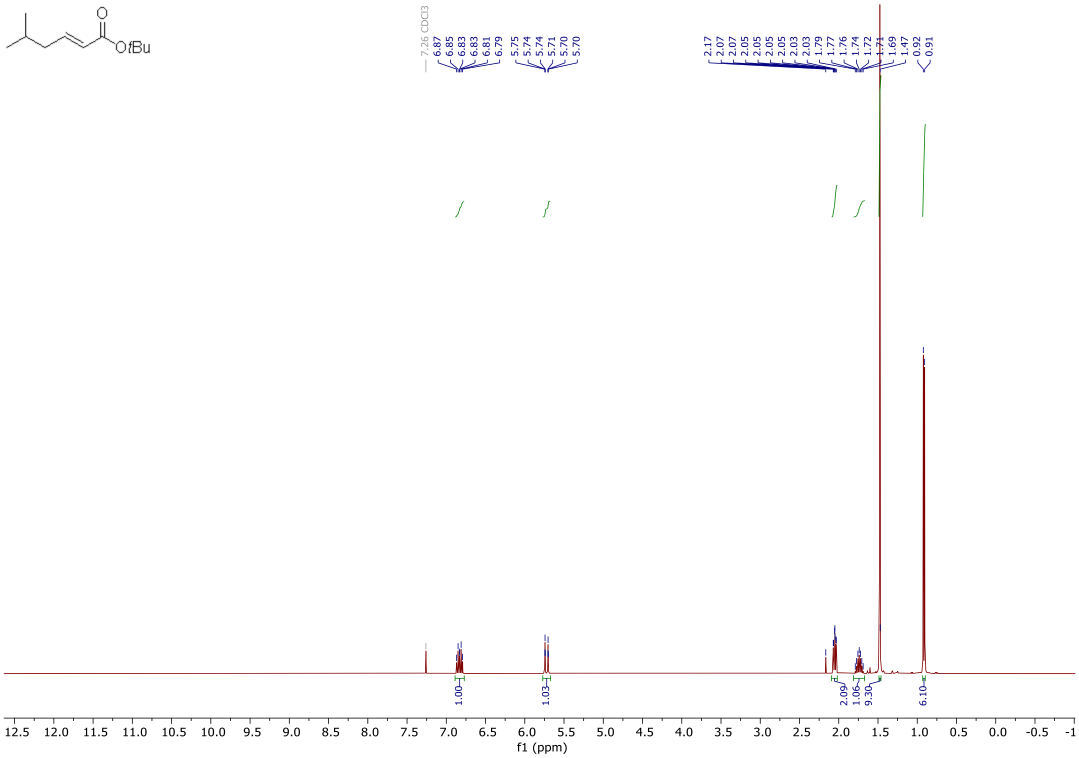

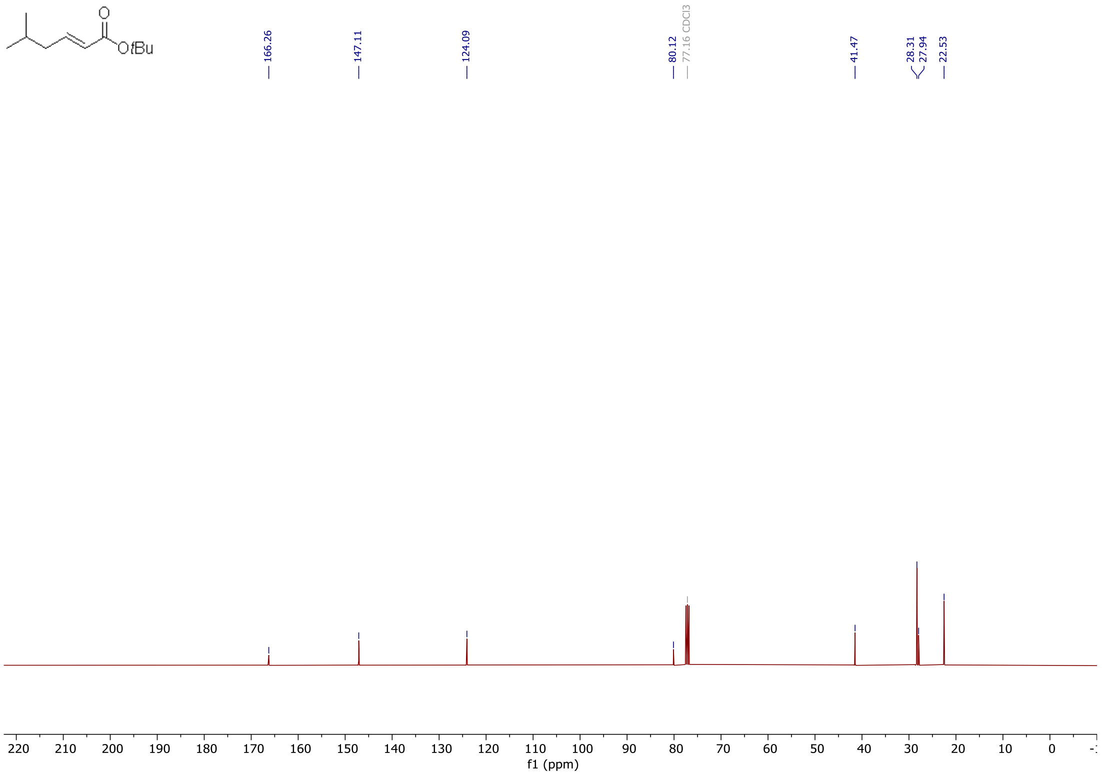

Figure S11: ^{1}H (400 MHz) and ^{13}C{^{1}H} (100 MHz) NMR spectra of *tert*-butyl (*S*)-2-(6-methoxynaphthalen-2-yl)propanoate (**1e**) in $CDCl_3$.

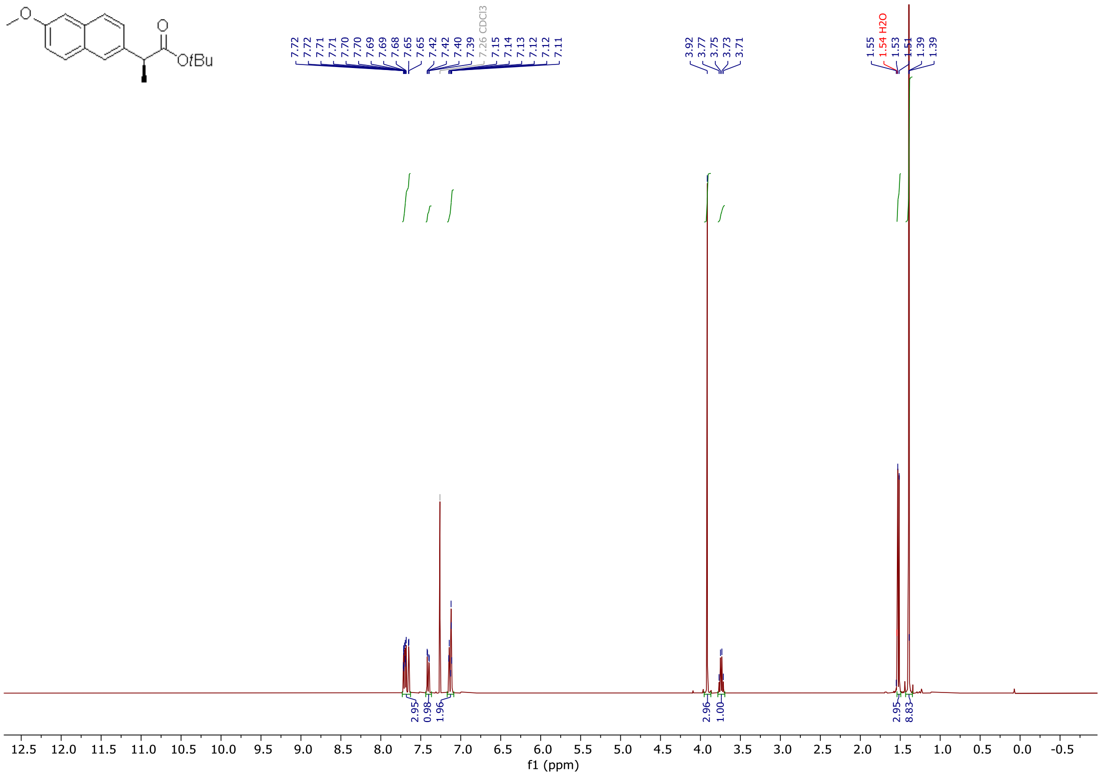

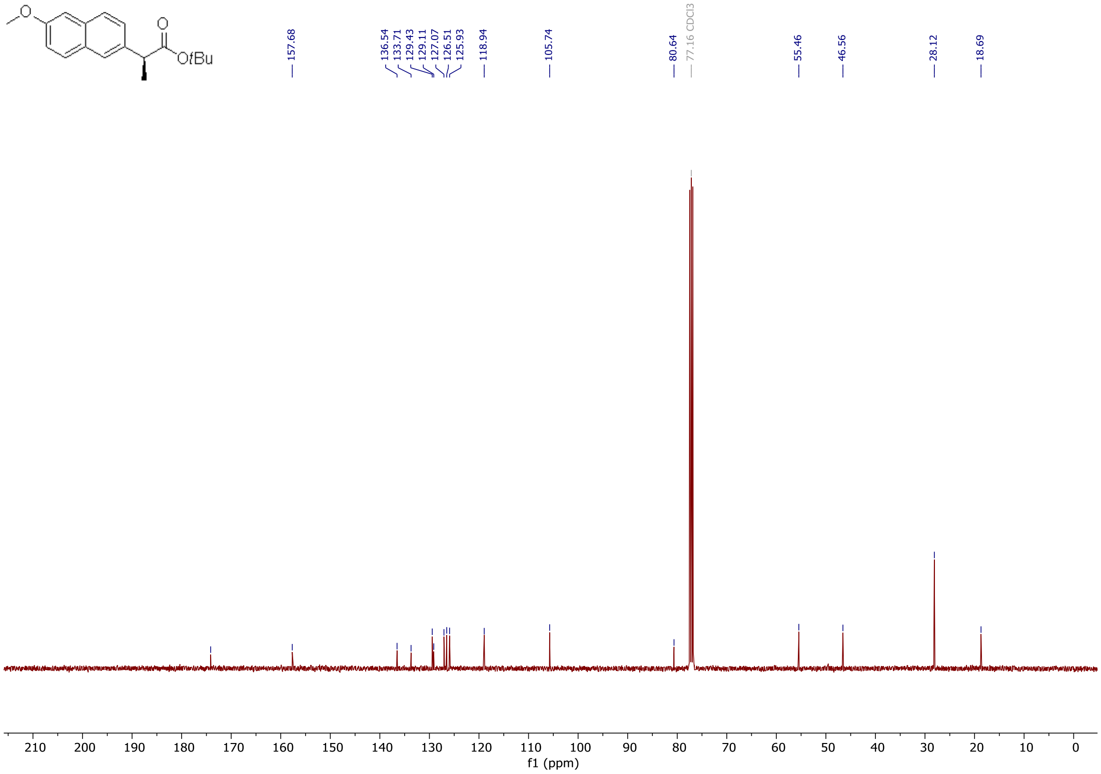

Figure S12: ^{1}H (400 MHz) and $^{13}C\{^{1}H\}$ (100 MHz) NMR spectra of *N*-[(1,1-dimethylethoxy)carbonyl]-L-alanine 1,1-dimethylethyl ester (**13**) in $CDCl_3$.

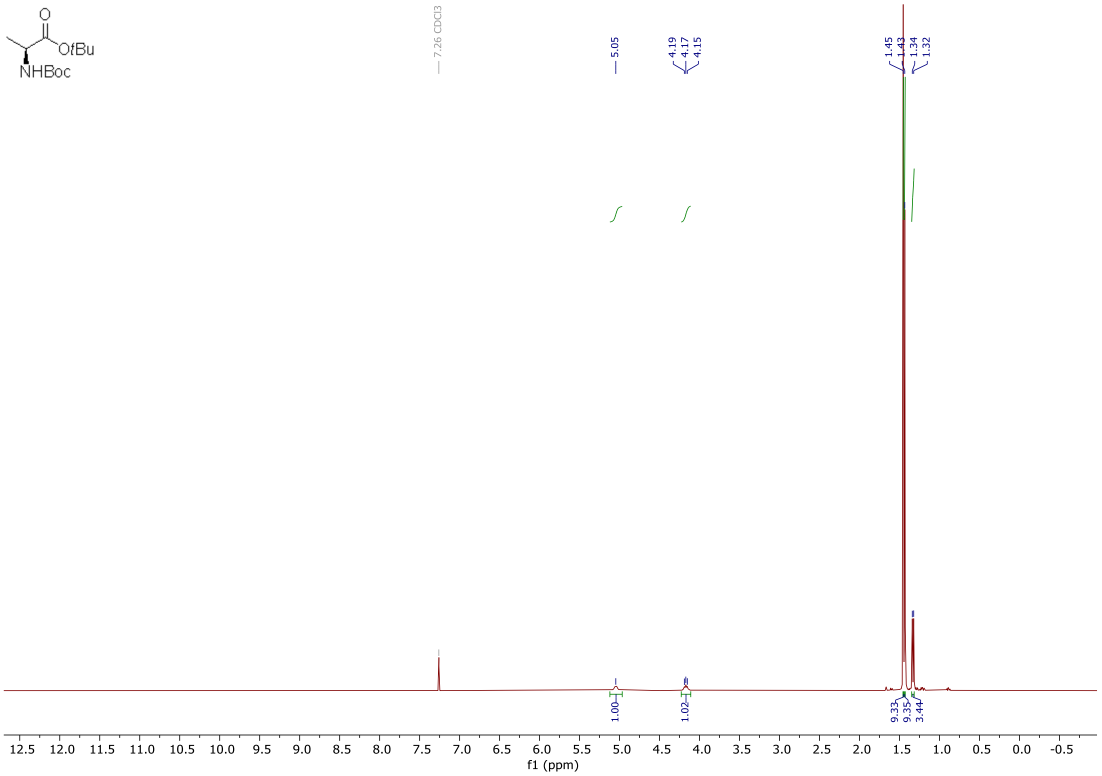

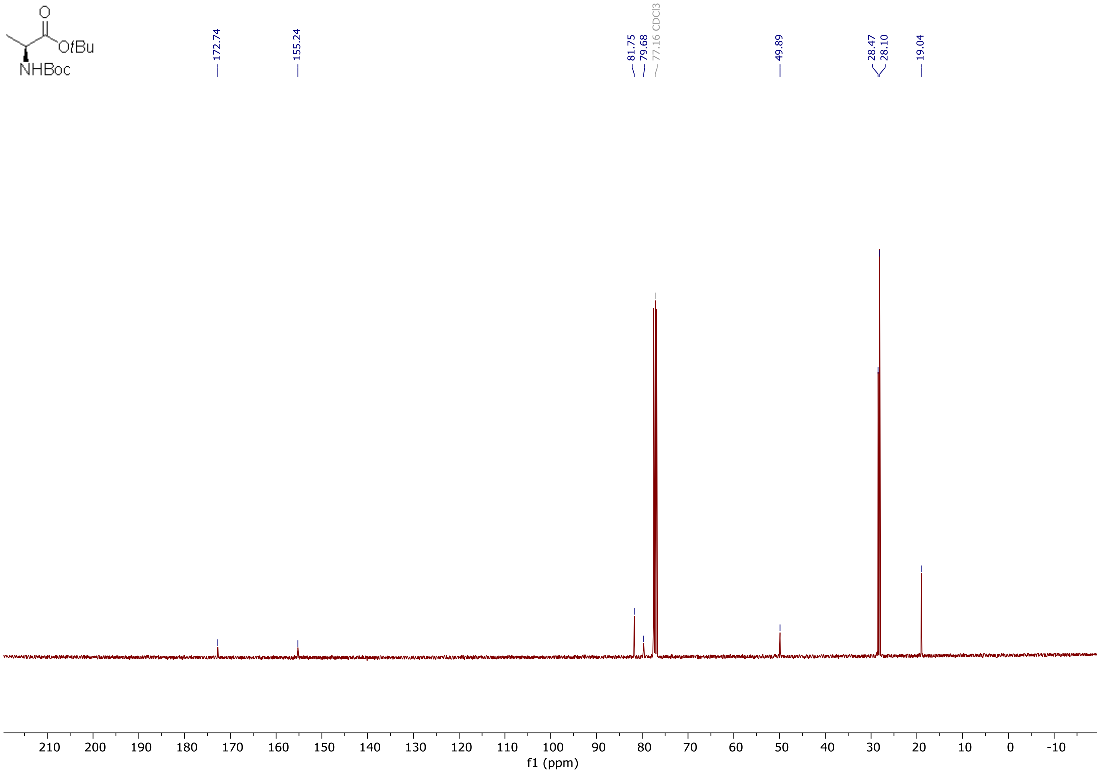

Figure S13: ^{1}H (400 MHz) and ^{13}C{^{1}H} (100 MHz) NMR spectra of 3-methylbut-2-en-1-yl (*S*)-2-(6-methoxynaphthalen-2-yl)propanoate in $CDCl_3$.

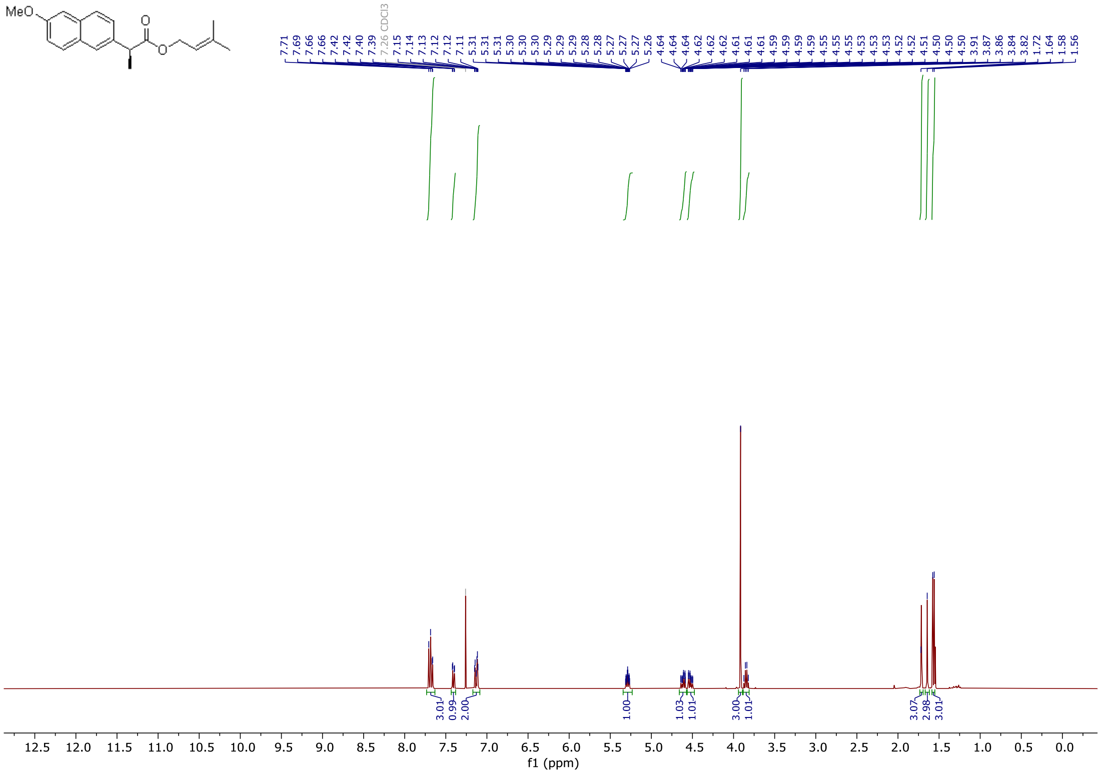

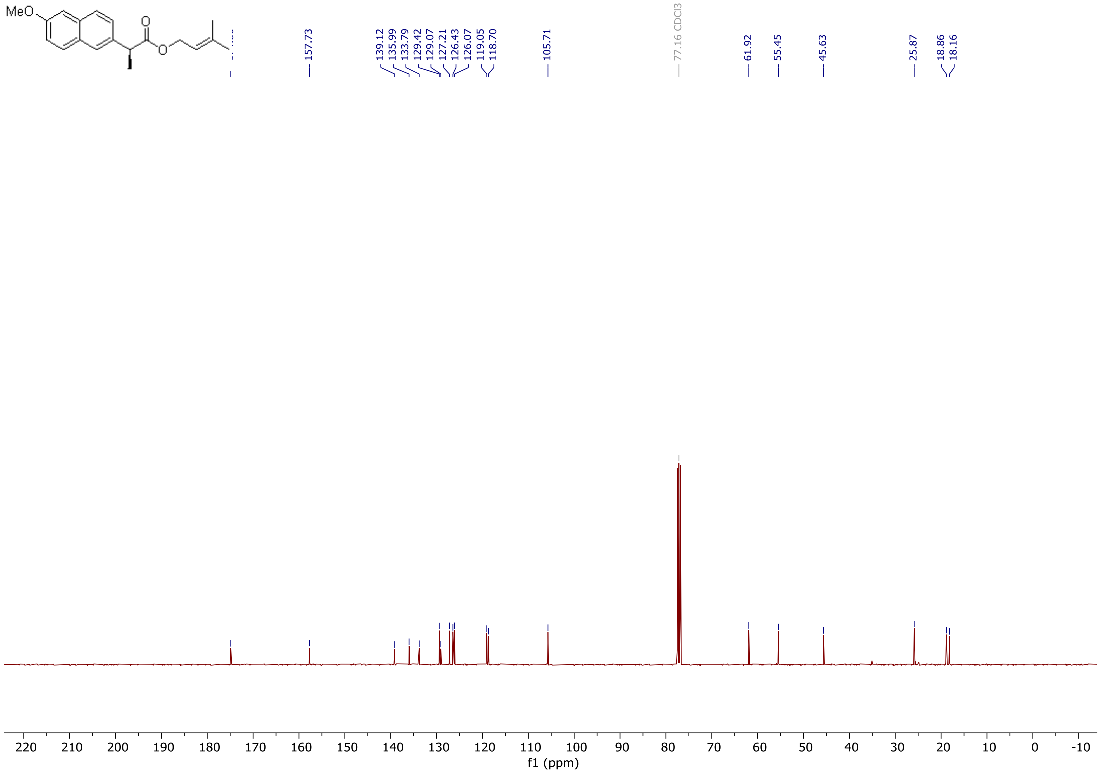

Figure S14: ^{1}H (400 MHz) and $^{13}C\{^{1}H\}$ (100 MHz) NMR spectra of ((3-phenylpropoxy)methanetriyl)tribenzene in $CDCl_3$.

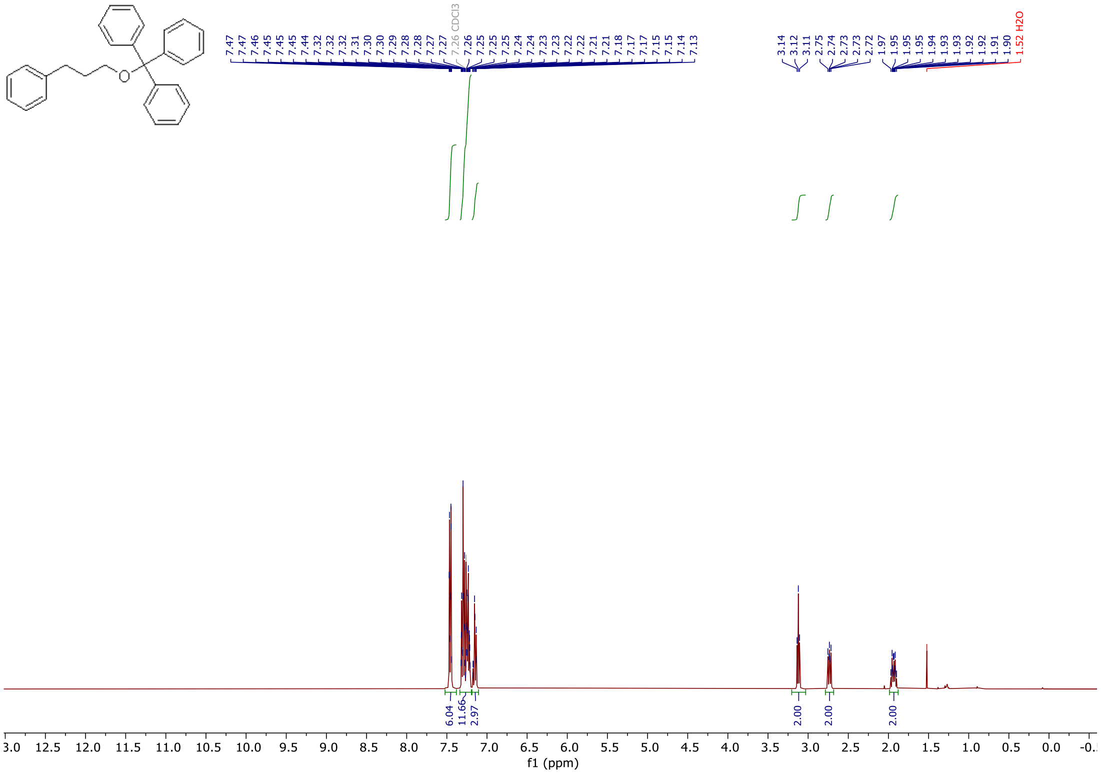

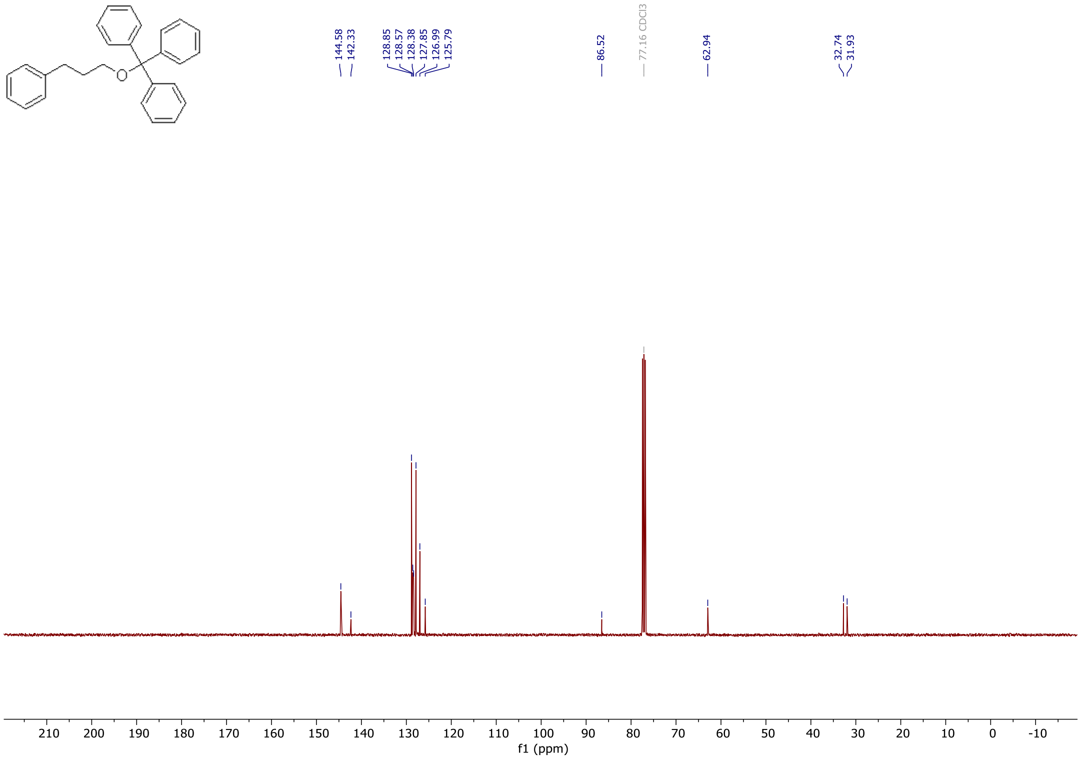

Figure S15: ^{1}H (400 MHz) and $^{13}C\{^{1}H\}$ (100 MHz) NMR spectra of 2-(3-Phenylpropoxy)tetrahydro-2*H*-pyran in $CDCl_3$.

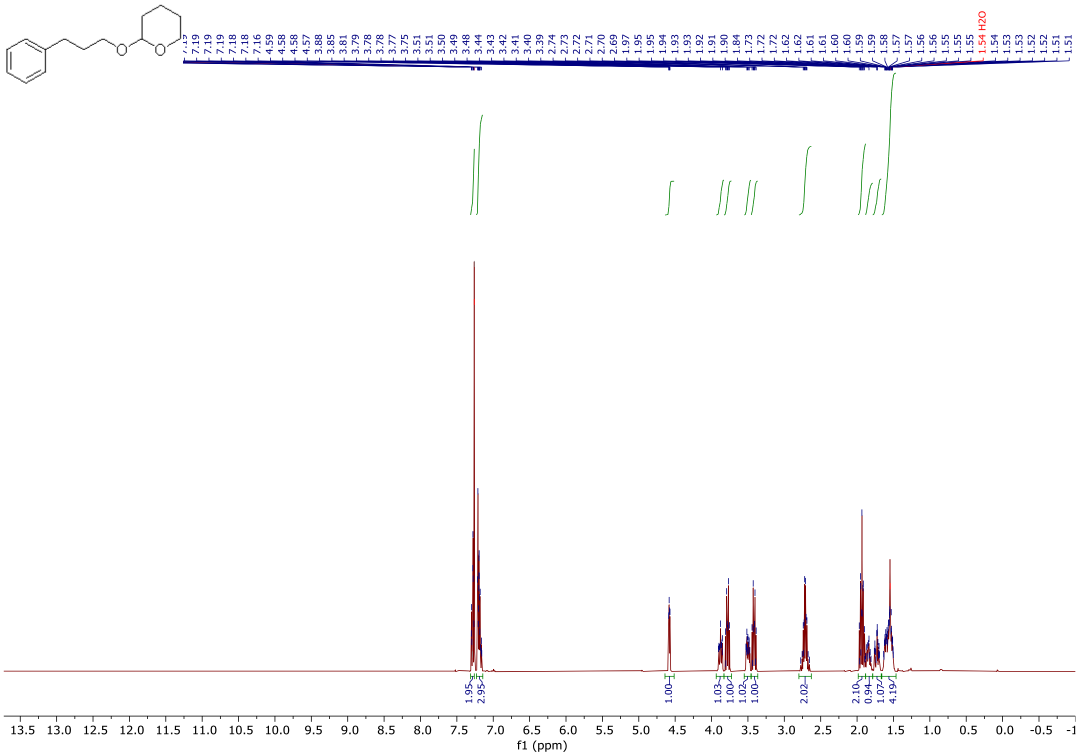

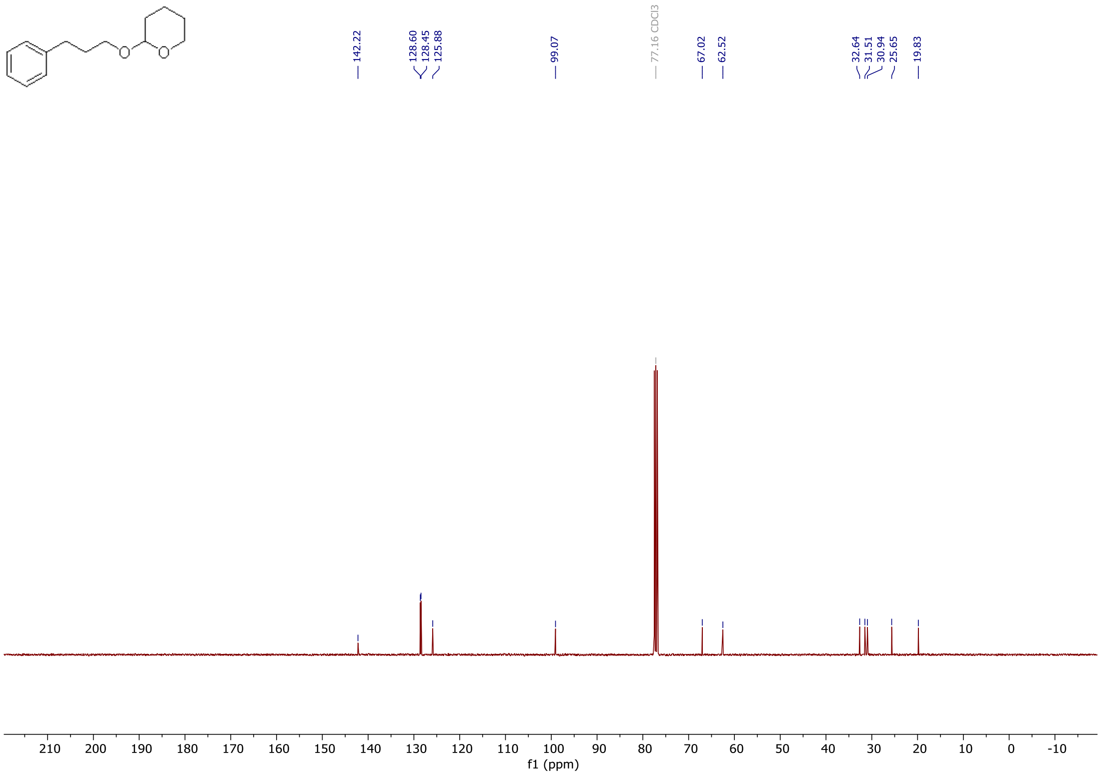

Figure S16: ^{1}H (400 MHz) and ^{13}C{^{1}H} (100 MHz) NMR spectra of (*S*)-5-(*N*-(2-(2-((((9*H*-fluoren-9-yl)methoxy)carbonyl)amino)-5-(*tert*-butoxy)-5-oxopentanamido)ethyl)sulfamoyl)-2-(6-(diethylamino)-3-(diethyl iminio)-3*H*-xanthen-9-yl)benzene sulfonate (**16**) in CD_3OD.

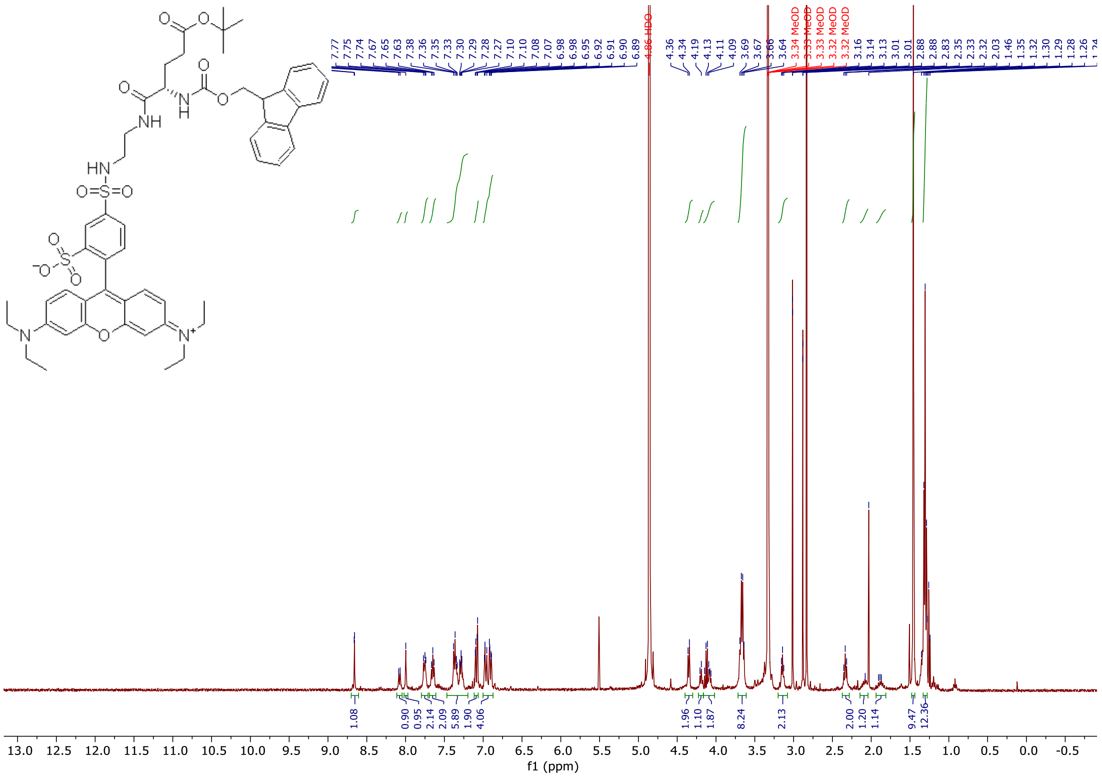

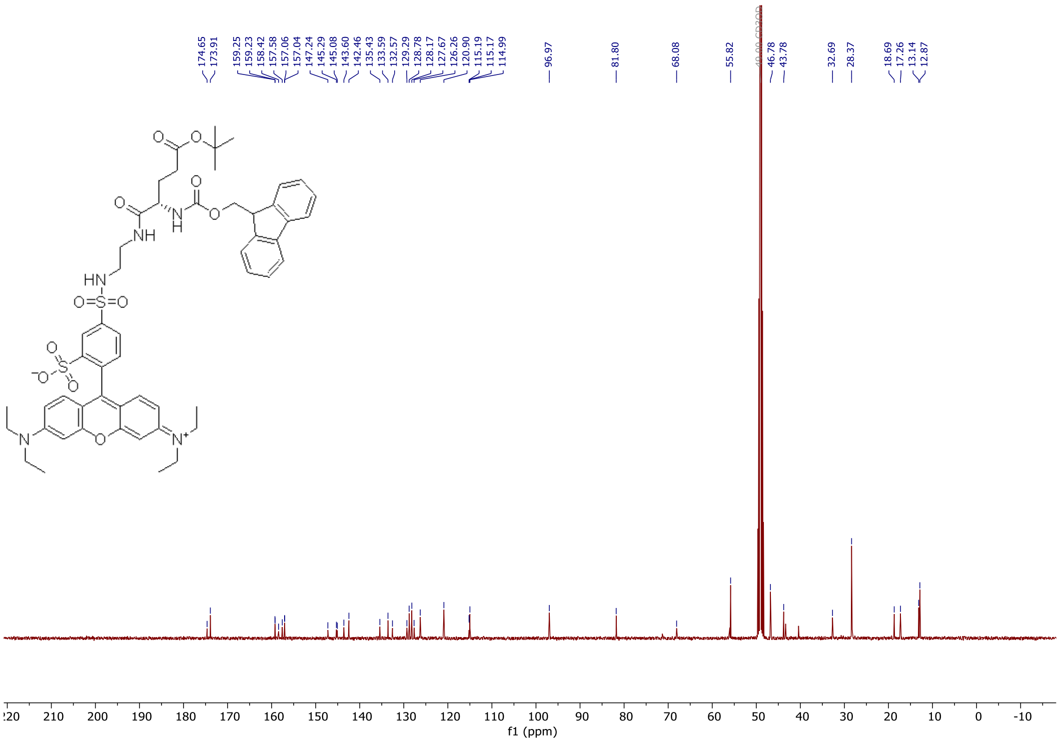

Figure S17: ^{1}H (400 MHz) and ^{13}C{^{1}H} (100 MHz) NMR spectra of *O*,*O'*-((5-(((3-(*tert*-butoxy)-3-oxopropanoyl)oxy)methyl) -1,3-phenylene)bis(methylene)) di-*tert*-butyl disuccinate (**18**) in $CDCl_3$.

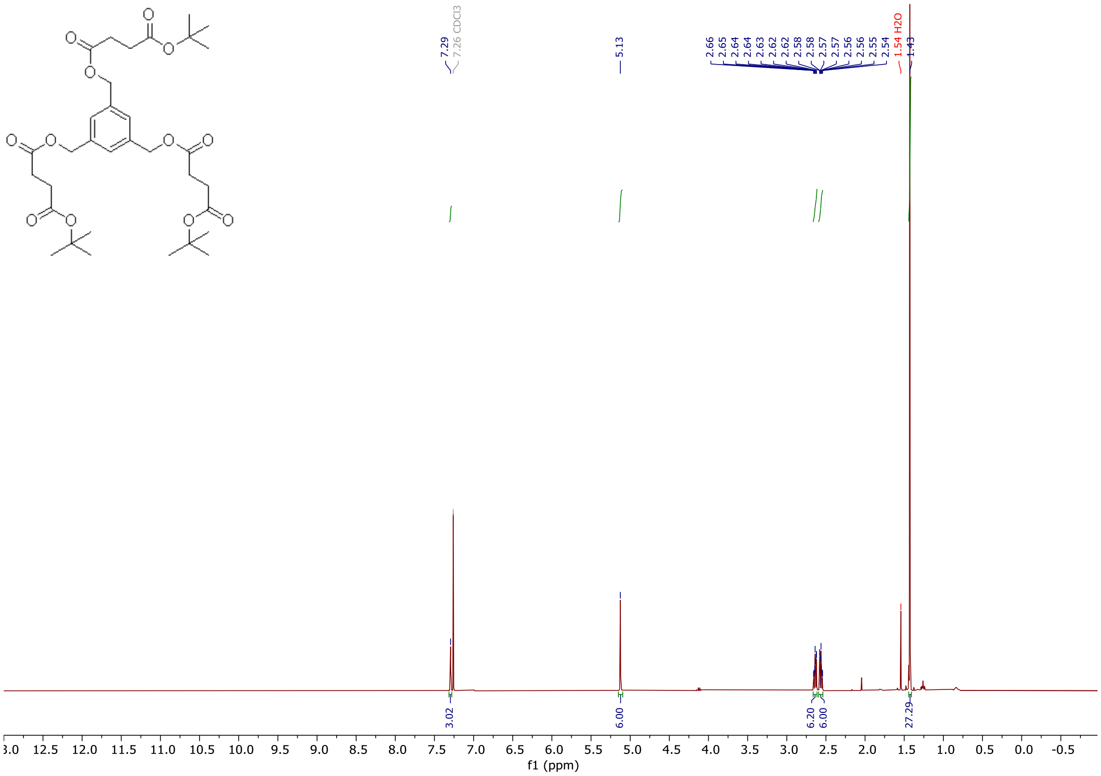

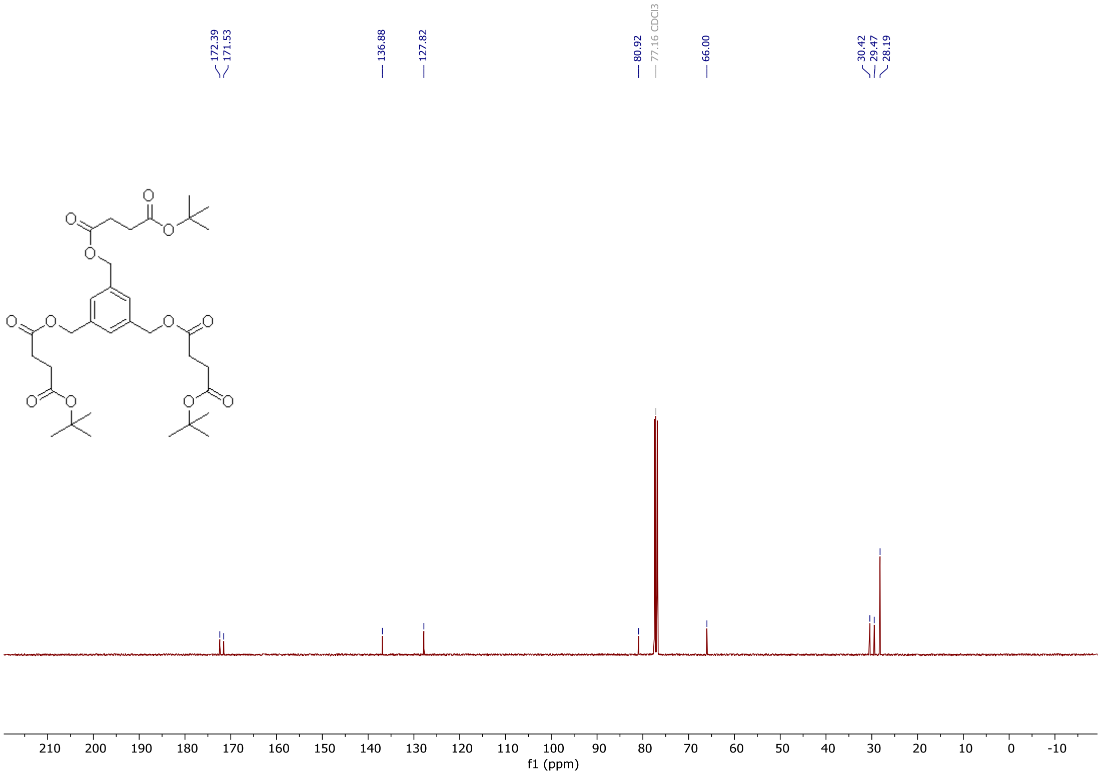

Figure S18: ^{1}H (400 MHz) and ^{13}C{^{1}H} (100 MHz) NMR spectra of acrylic acid (**2a**) in $CDCl_3$.

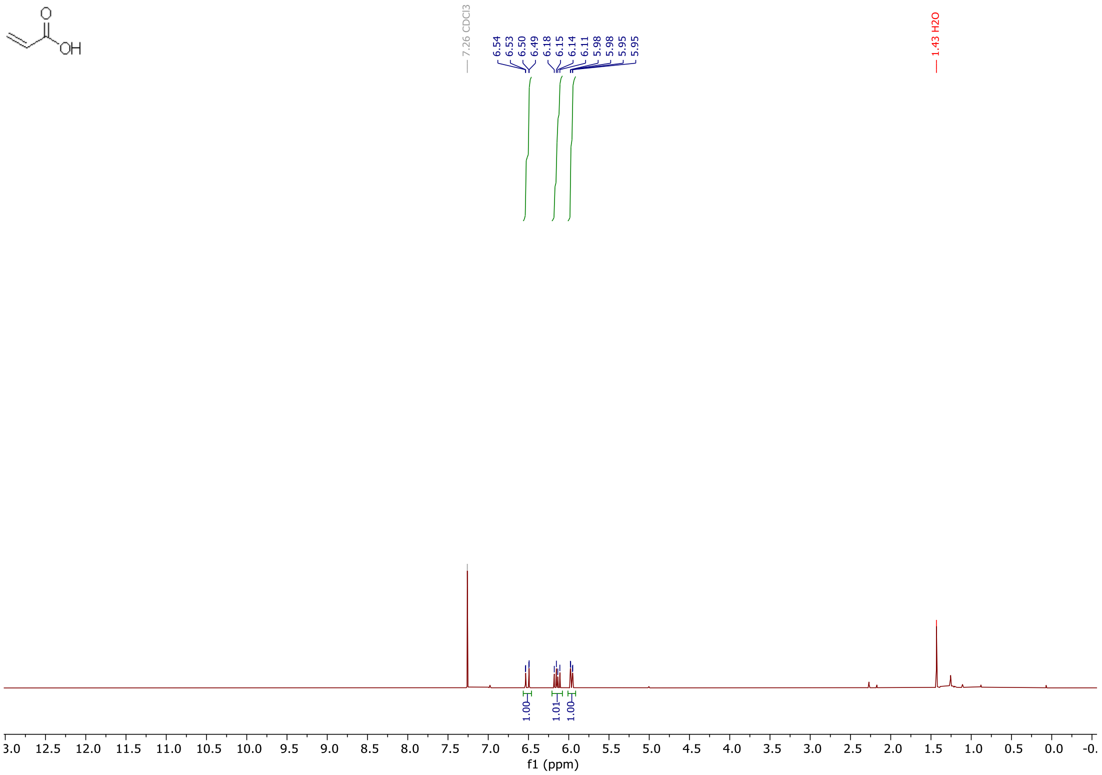

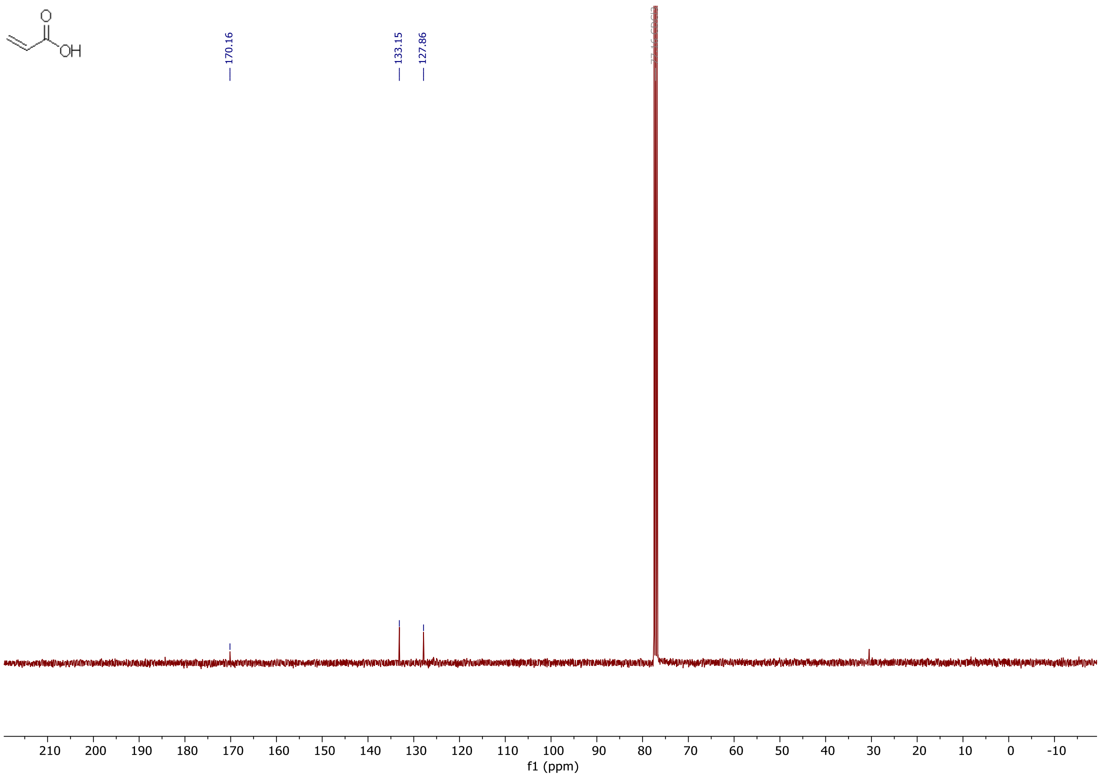

Figure S19: ^{1}H (400 MHz) and ^{13}C{^{1}H} (100 MHz) NMR spectra of (*E*)-but-2-enoic acid (**2b**) in $CDCl_3$.

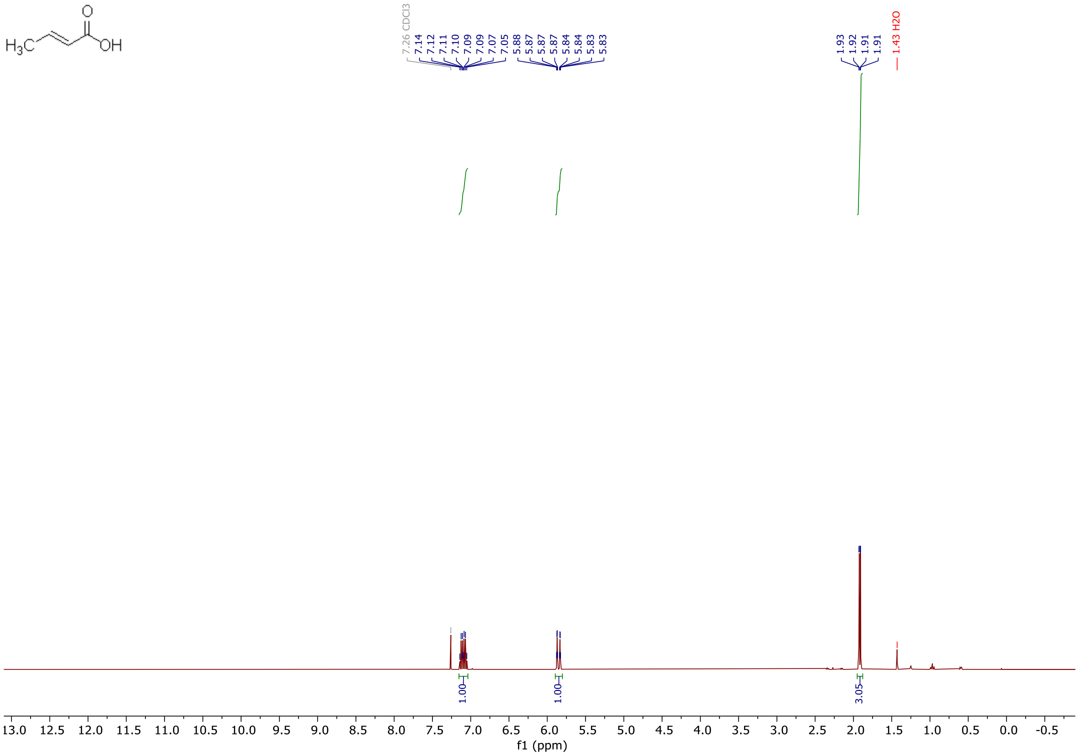

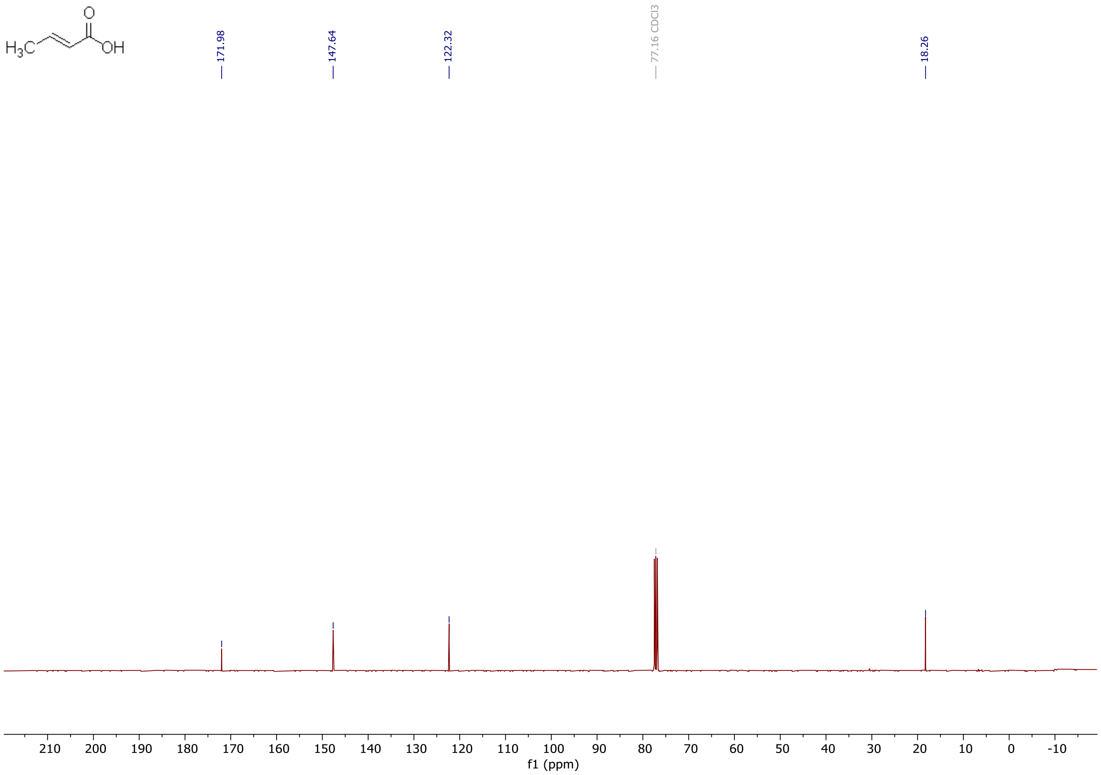

Figure S20: ^{1}H (400 MHz) and $^{13}C\{^{1}H\}$ (100 MHz) NMR spectra of (*E*)-5-methyl-2-hexenoic acid (**2c**) in $CDCl_3$.

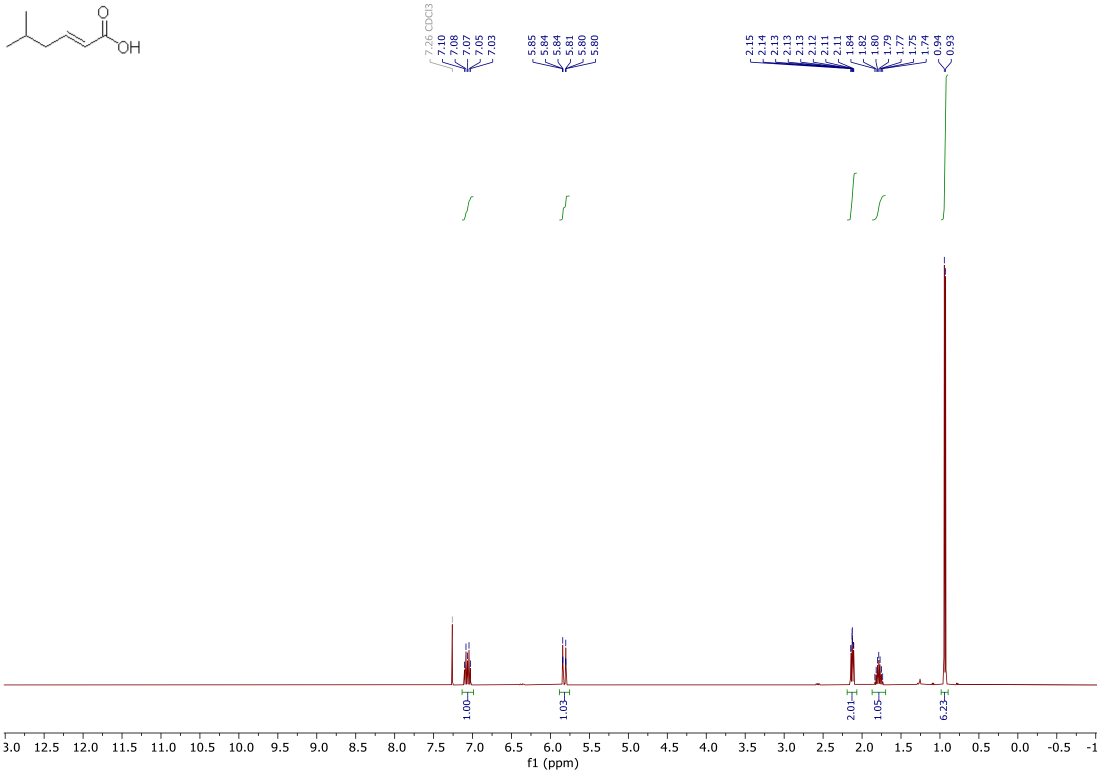

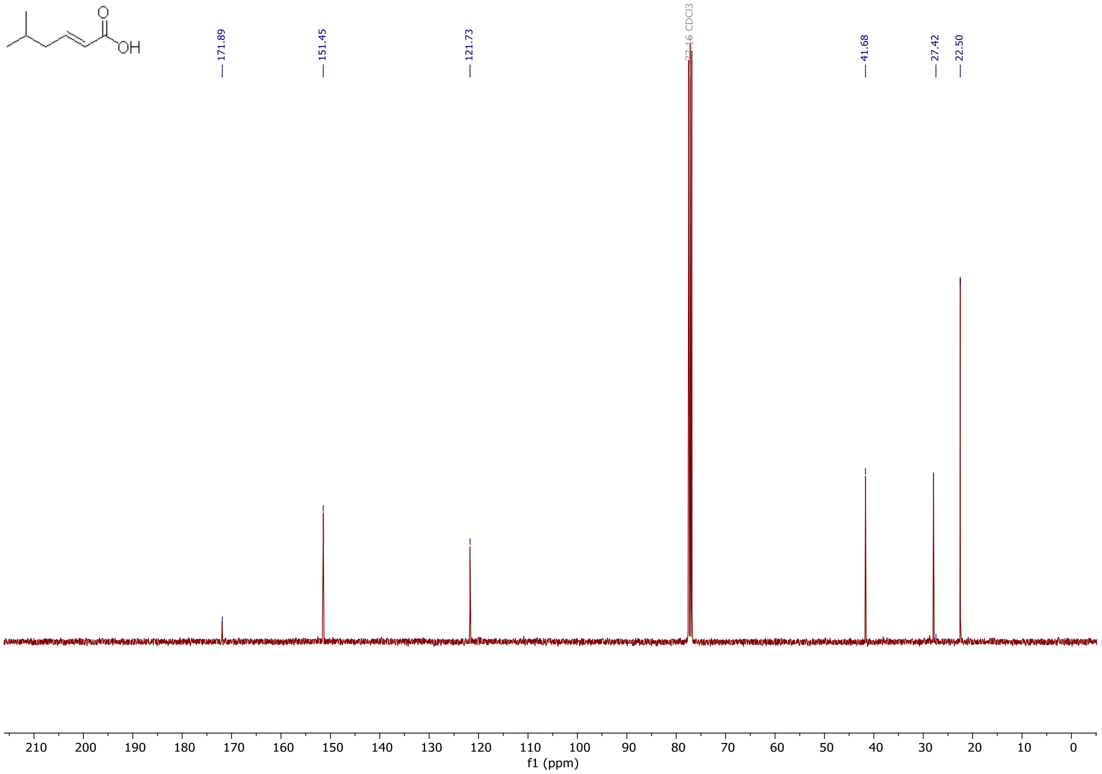

Figure S21: ^{1}H (400 MHz) and $^{13}C\{^{1}H\}$ (100 MHz) NMR spectra of 3-(fur-2-yl)crotonic acid (**2d**) in $CDCl_3$.

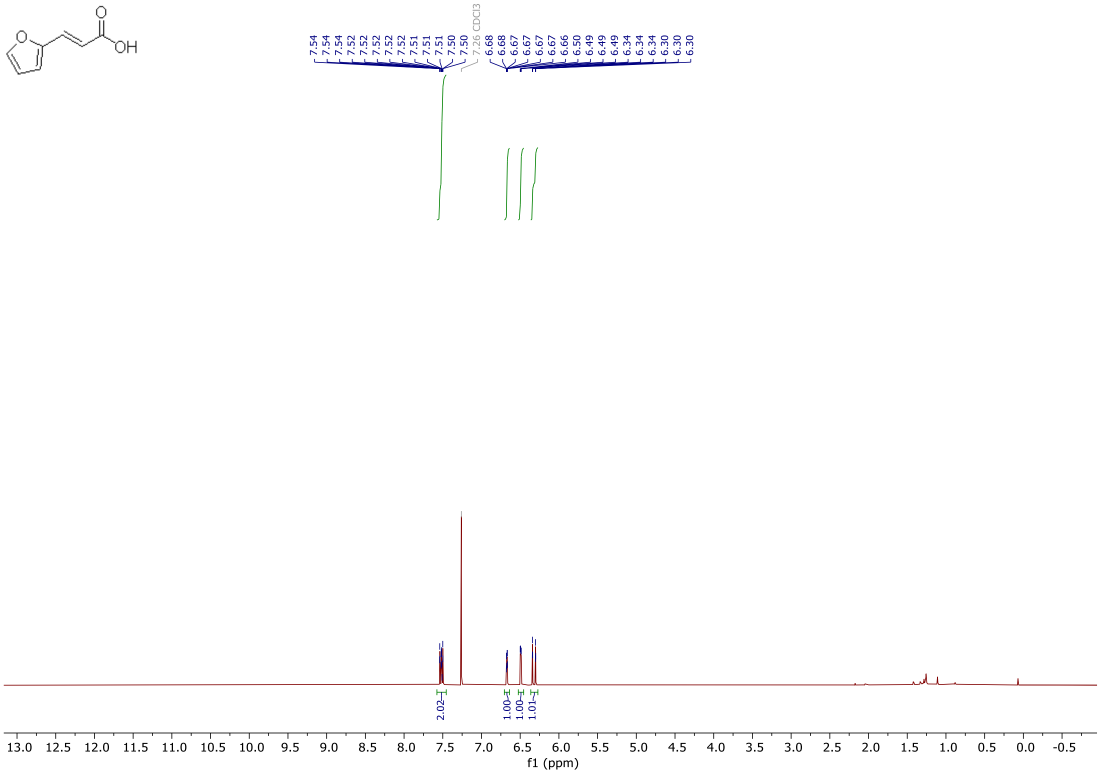

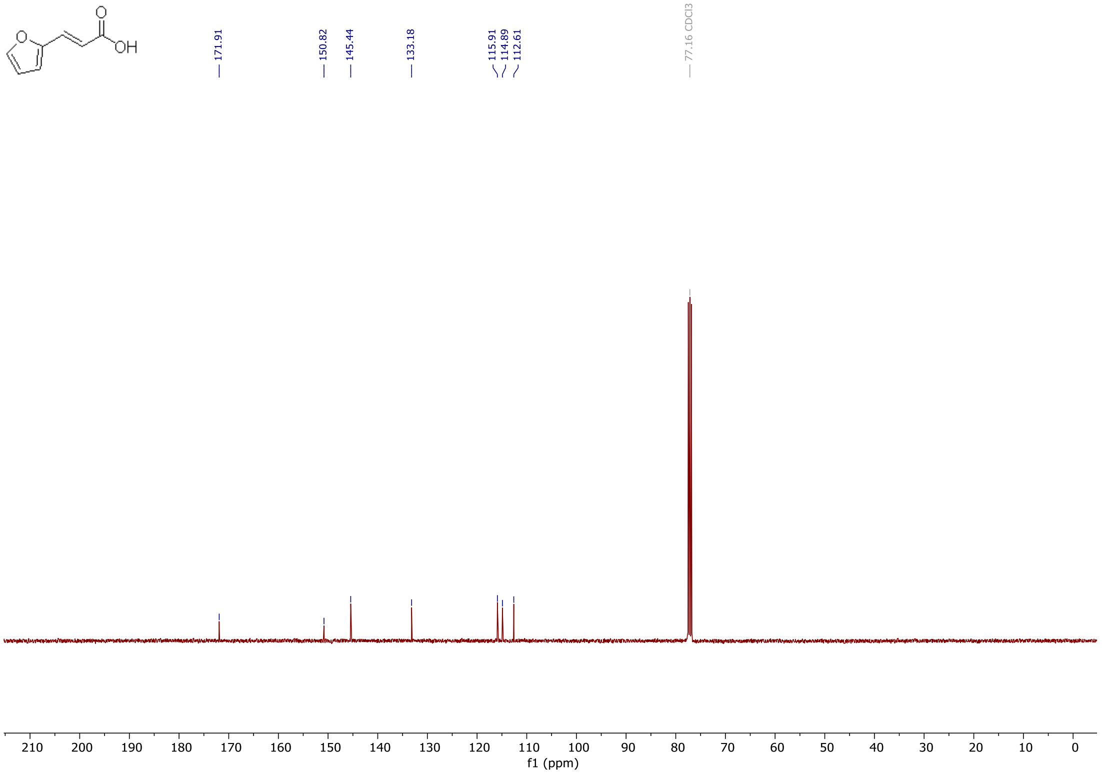

Figure S22: ^{1}H (400 MHz) and $^{13}C\{^{1}H\}$ (100 MHz) NMR spectra of (2*S*)-2-(6-methoxy(2-naphthyl))propanoic acid (**2e**) in $CDCl_3$.

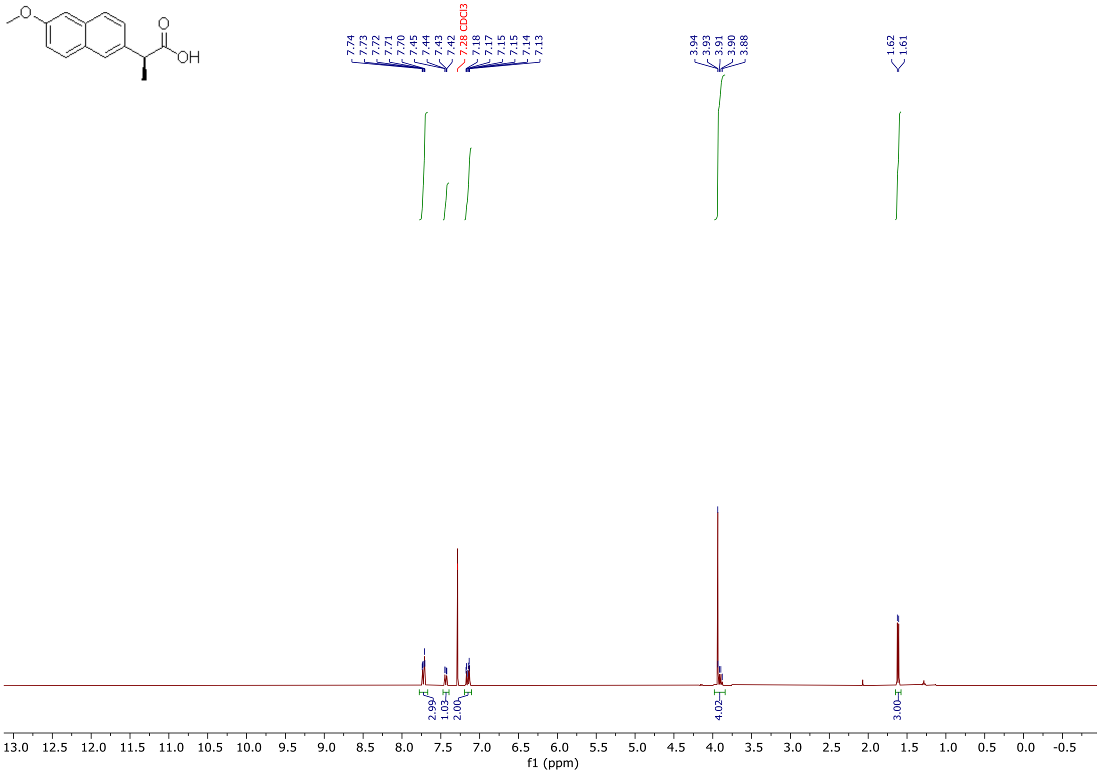

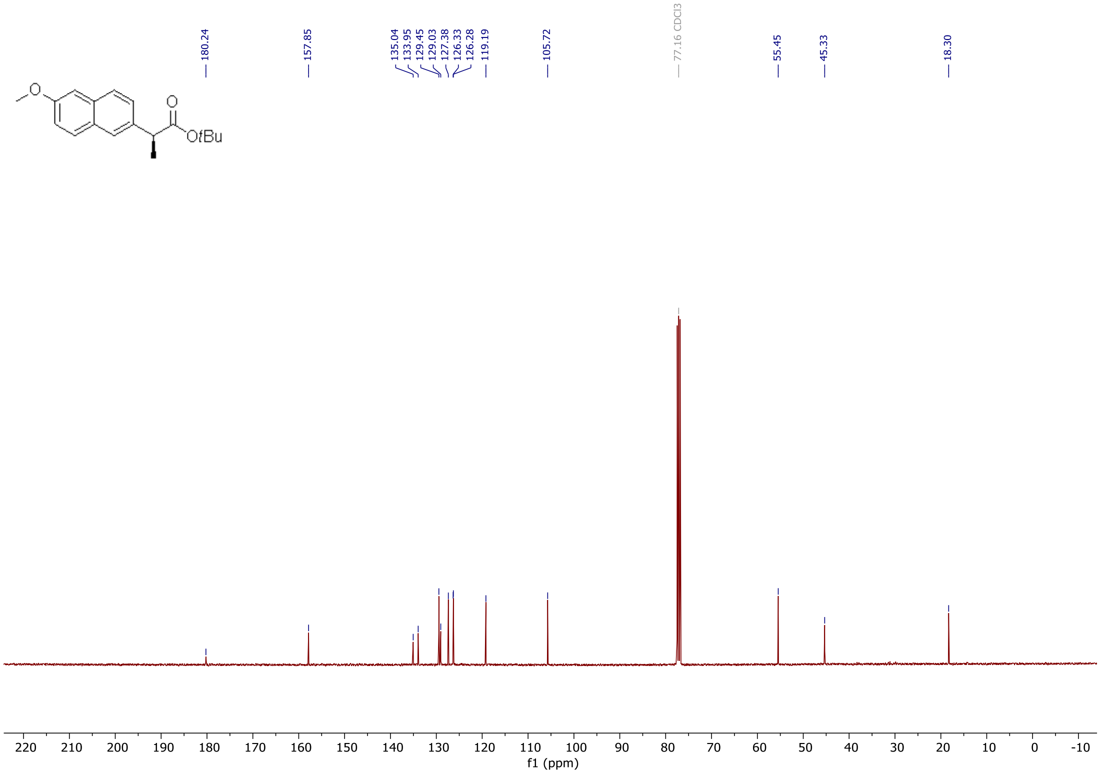

Figure S23: ^{1}H (400 MHz) and ^{13}C{^{1}H} (100 MHz) NMR spectra of 3-phenyl-1-propanol (**4a**) in $CDCl_3$.

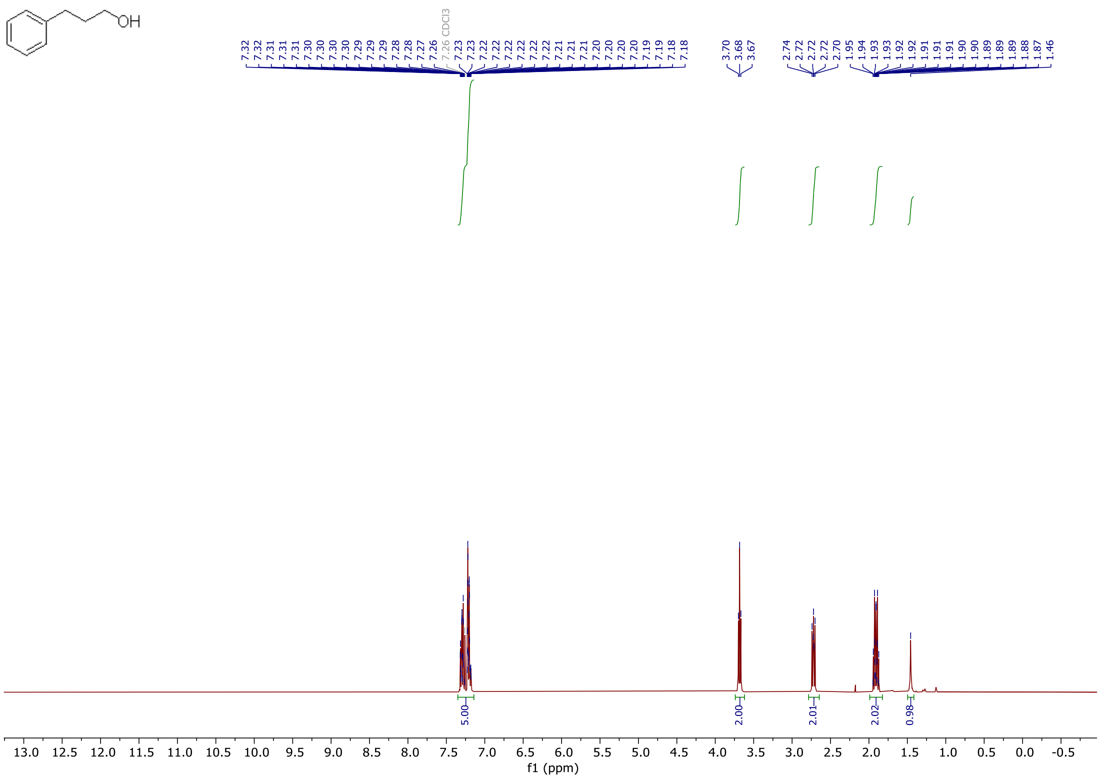

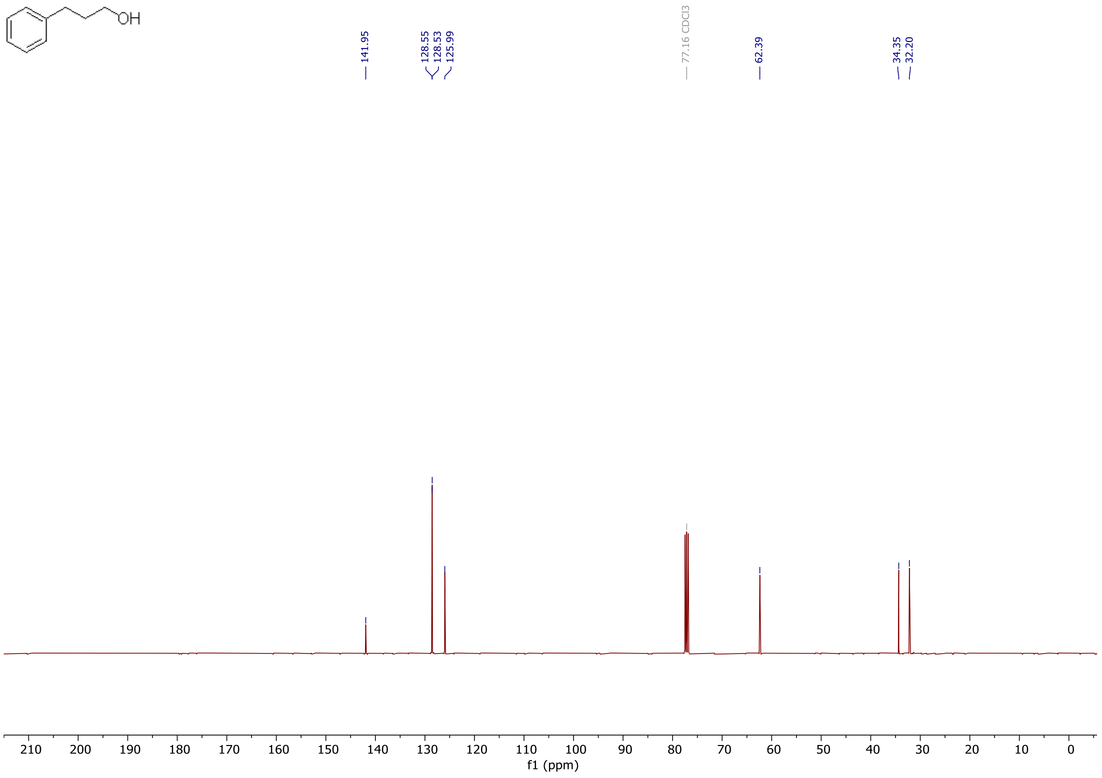

Figure S24: ^{1}H (400 MHz) and ^{13}C{^{1}H} (100 MHz) NMR spectra of benzyl alcohol (**4b**) in $CDCl_3$.

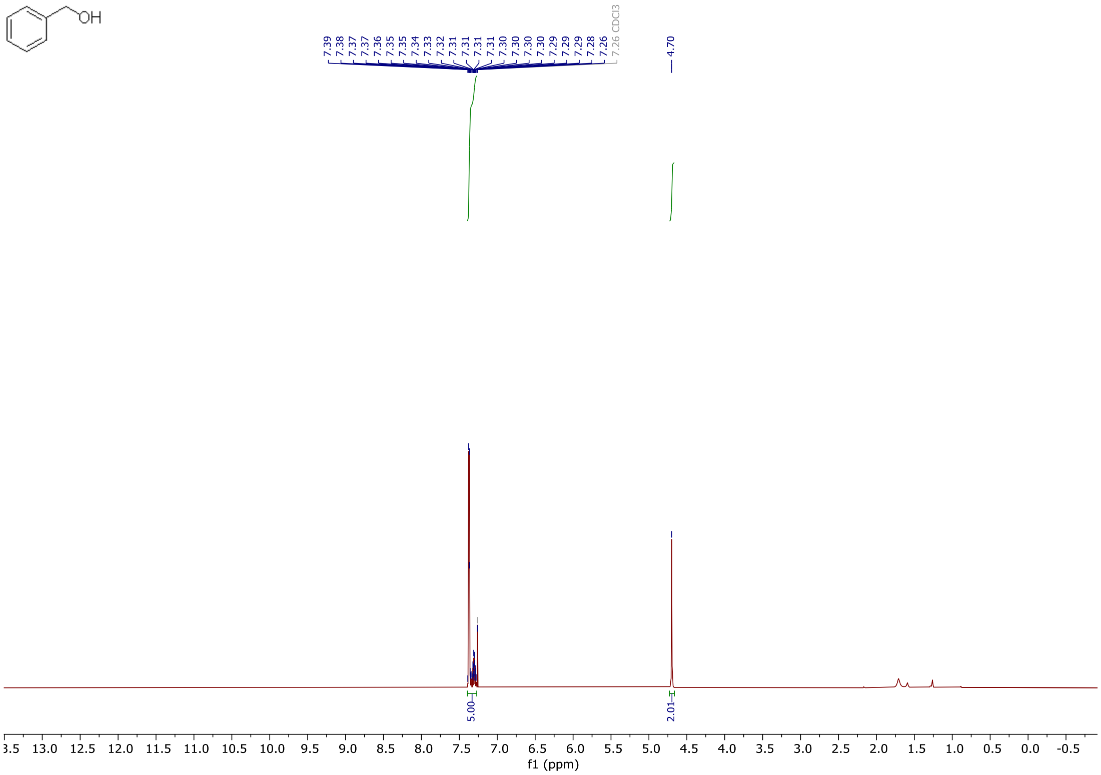

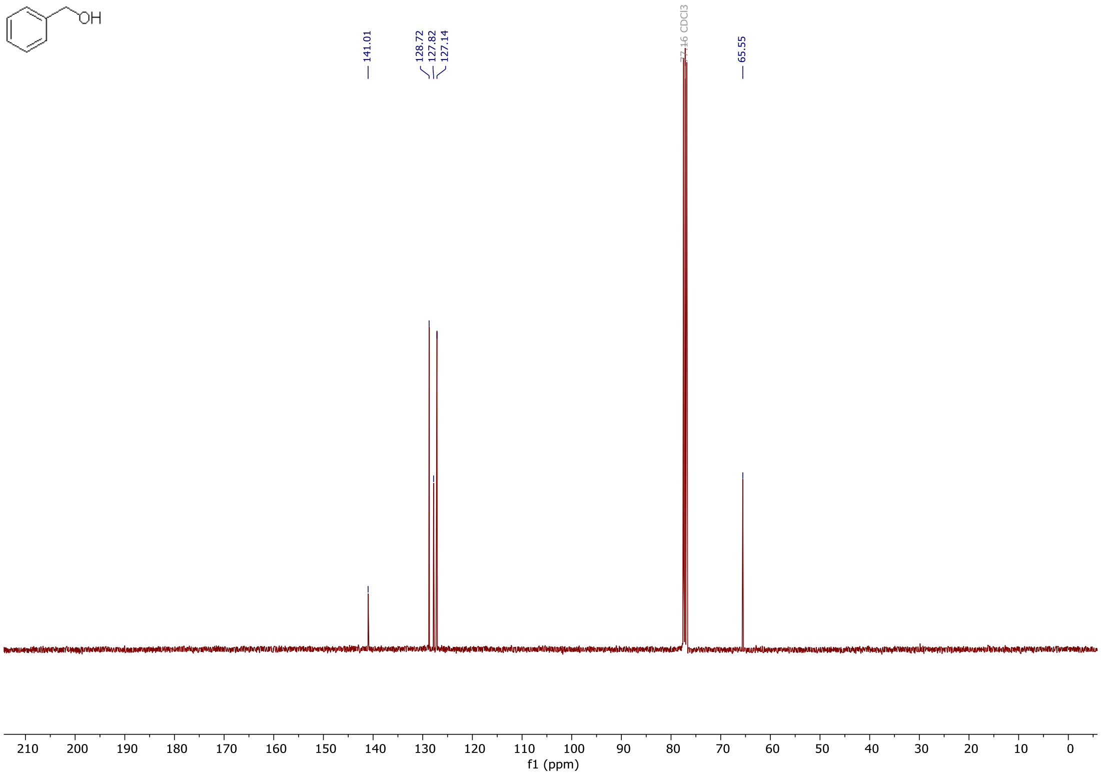

Figure S25: ^{1}H (400 MHz) and ^{13}C{^{1}H} (100 MHz) NMR spectra of *N*-methyl-*p*-toluidine (**7**) in $CDCl_3$.

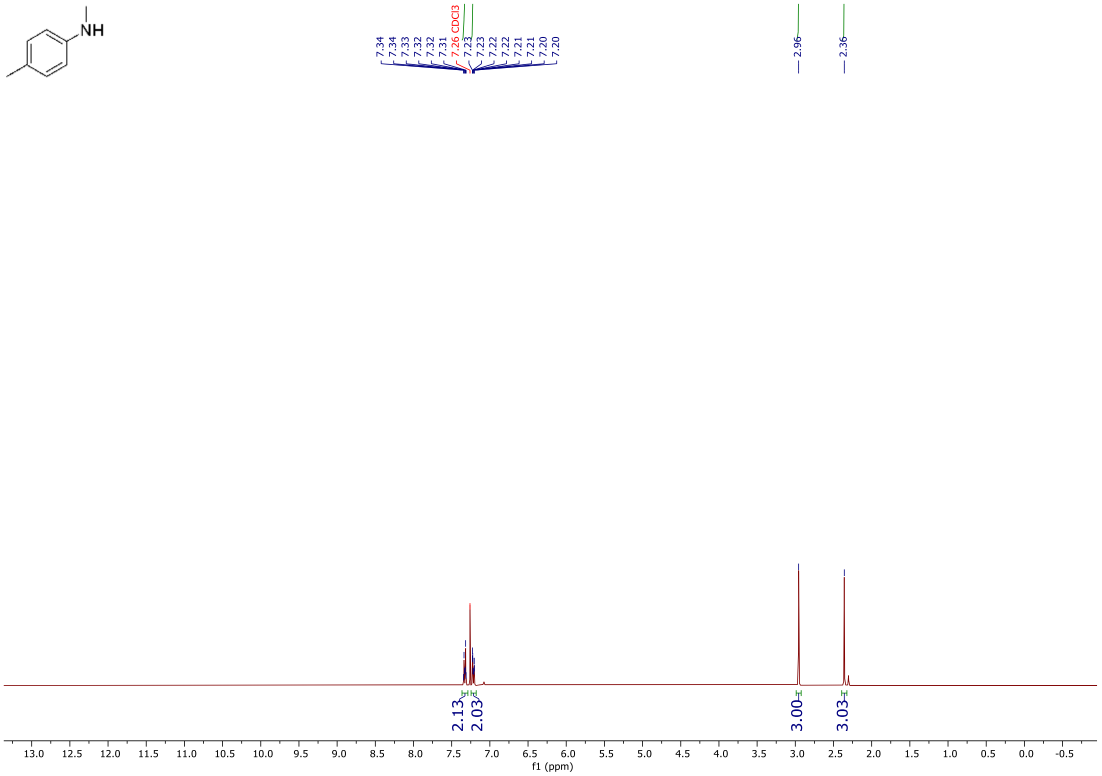

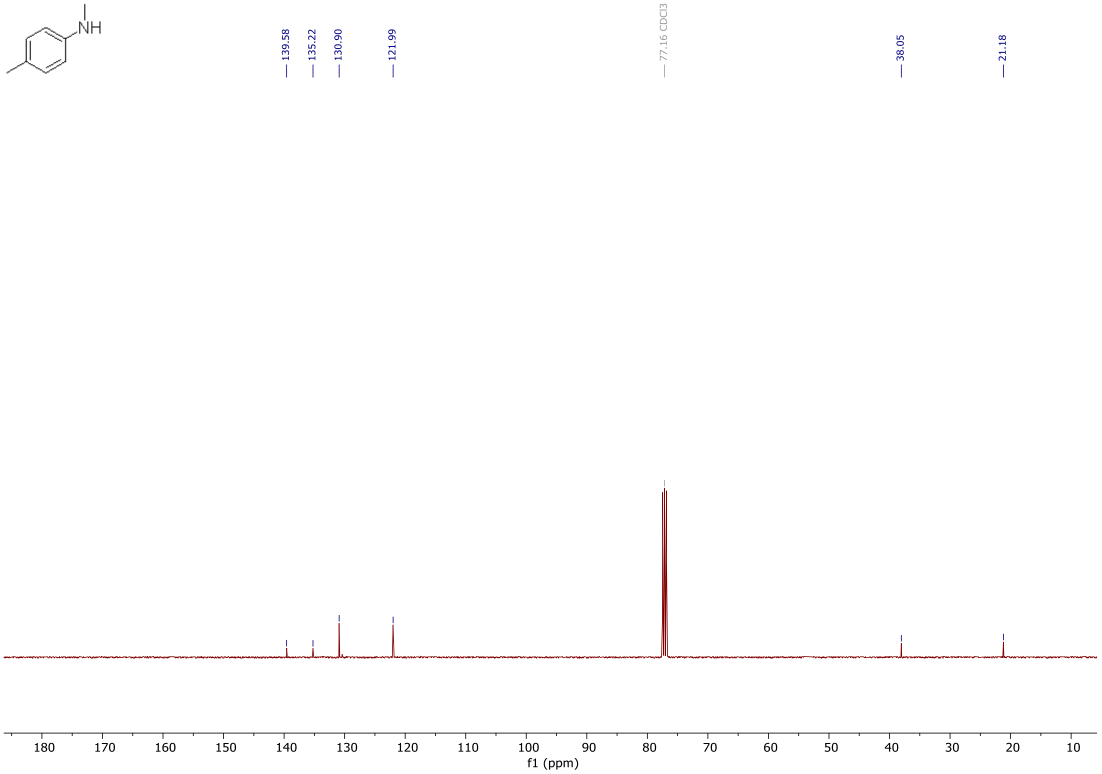

Figure S26: ^{1}H (400 MHz) and ^{13}C{^{1}H} (100 MHz) NMR spectra of *N*-methyl-1-phenylmethanamine (**9**) in $CDCl_3$.

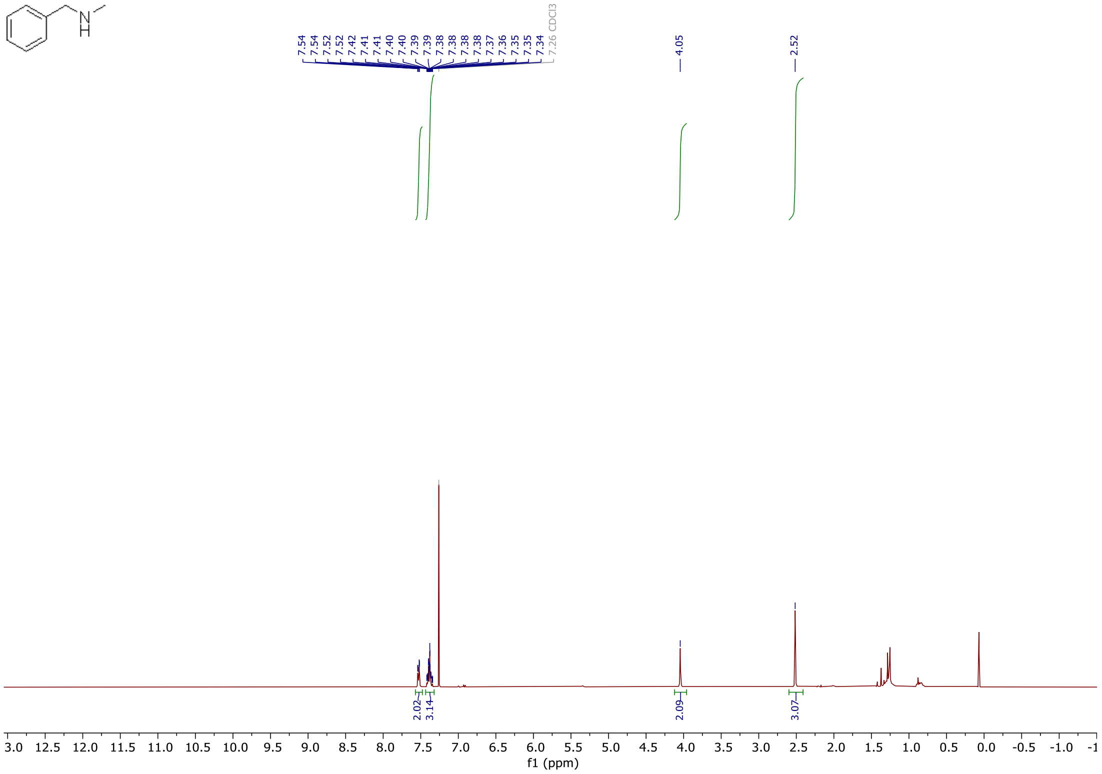

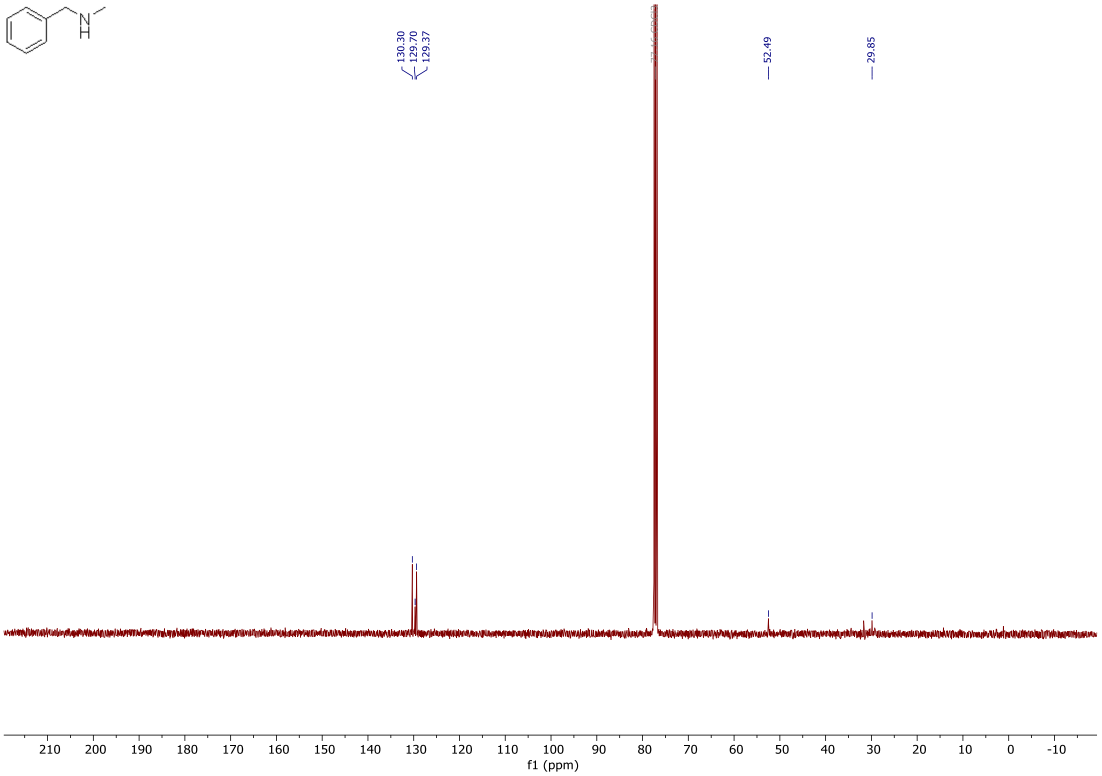

Figure S27: ^{1}H (400 MHz) and ^{13}C{^{1}H} (100 MHz) NMR spectra of butanedioic acid, monoethyl ester (**11**) in $CDCl_3$.

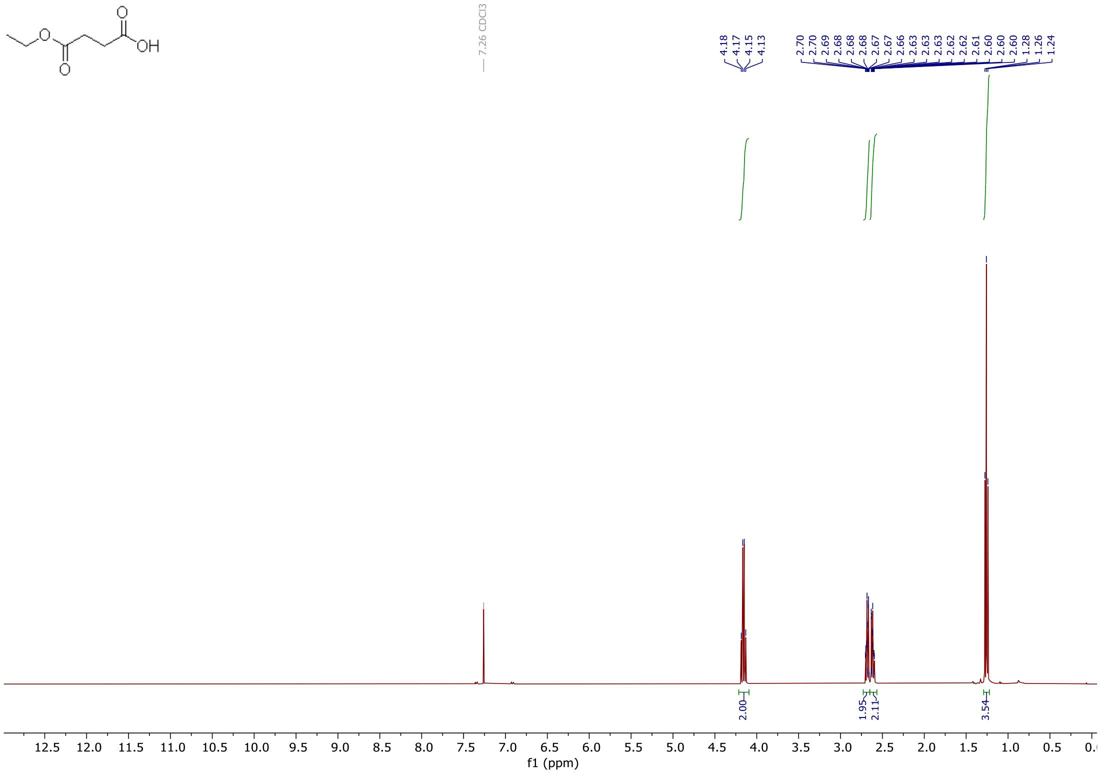

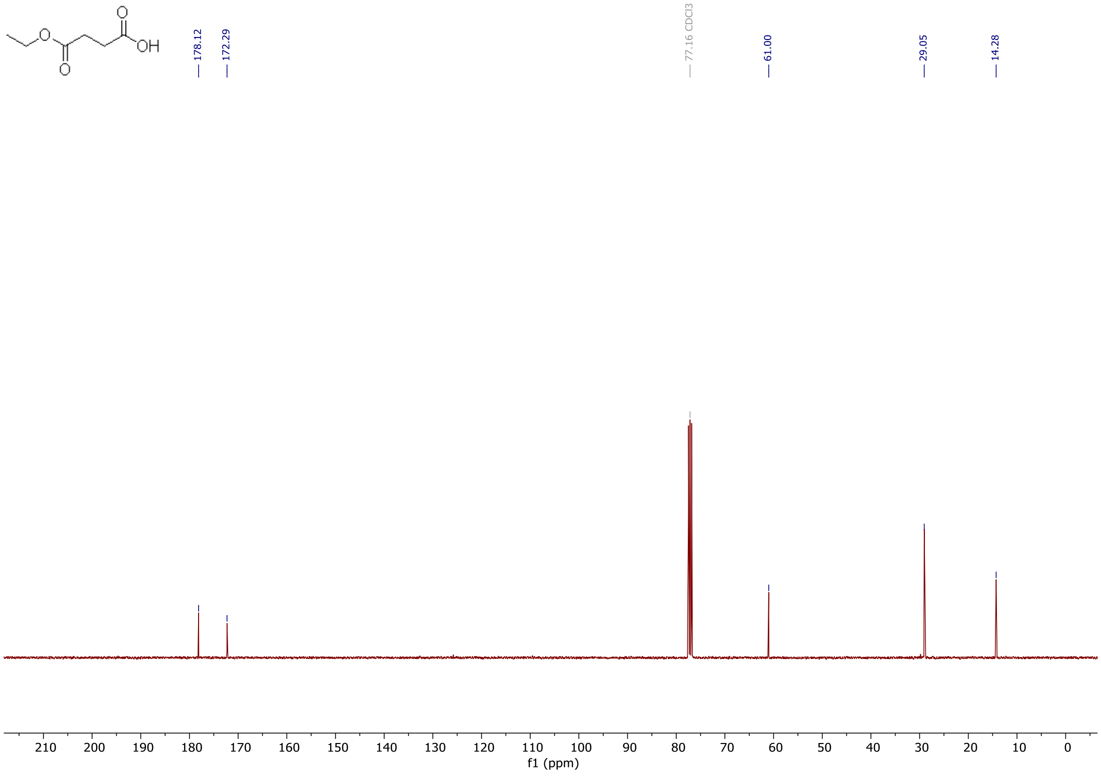

Figure S28: ^{1}H (400 MHz) and ^{13}C{^{1}H} (100 MHz) NMR spectra of *tert*-butyl L-alaninate (**13**) in CD_3CN.

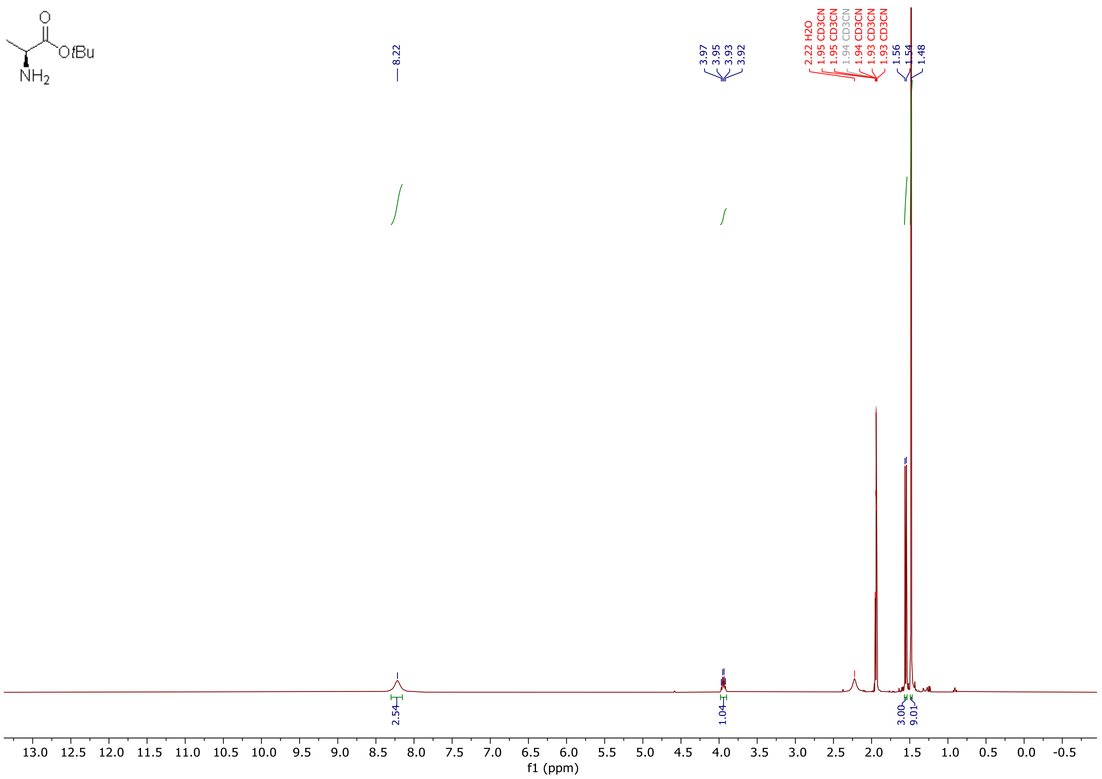

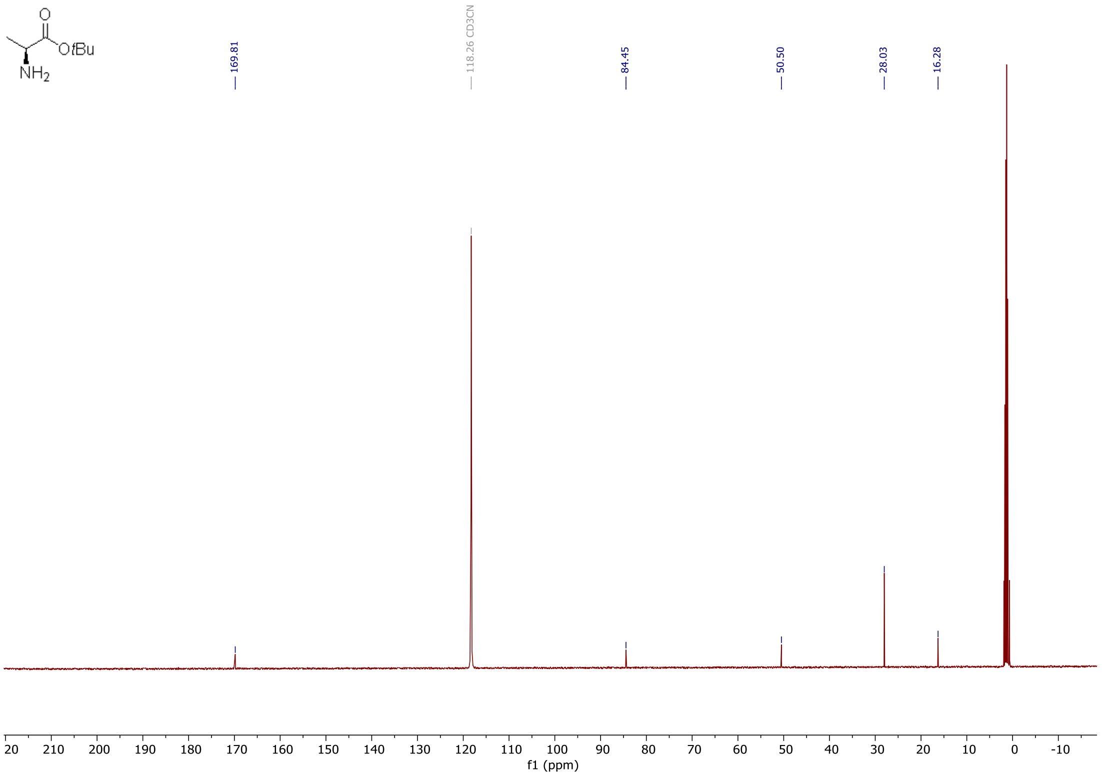

Figure S29: ^{1}H (400 MHz) and ^{13}C{^{1}H} (100 MHz) NMR spectra of methyl (*E*)-5-methylhex-2-enoate (**15**) in $CDCl_3$.

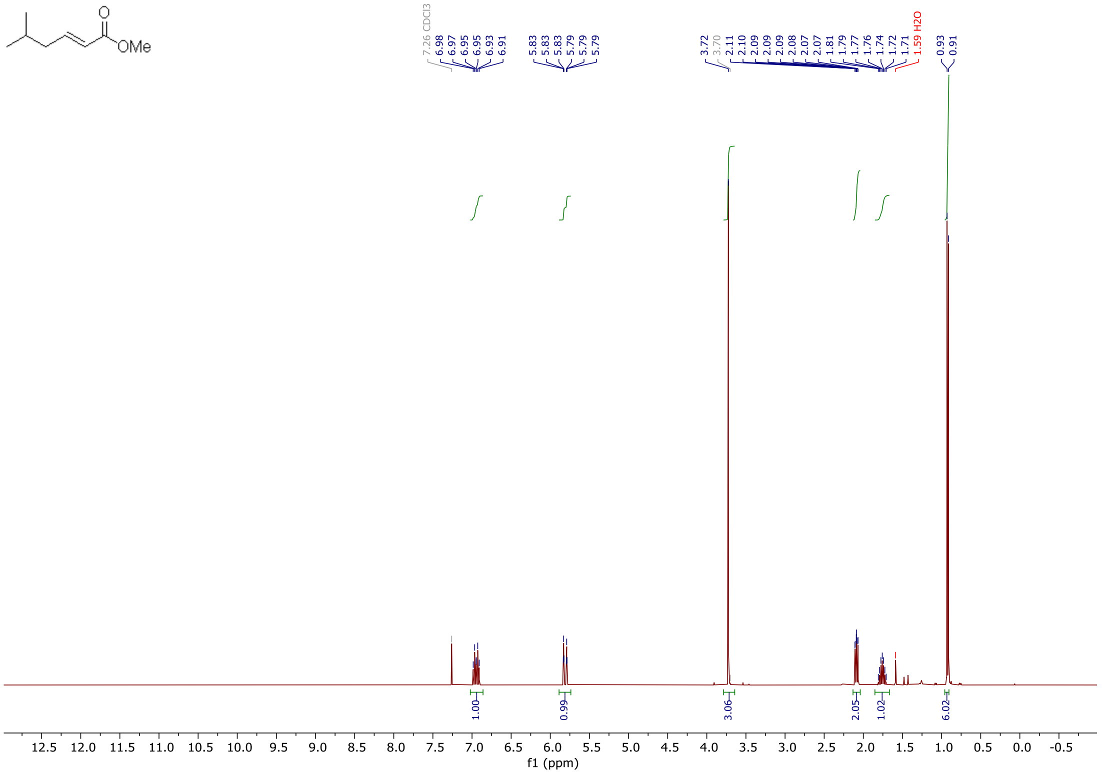

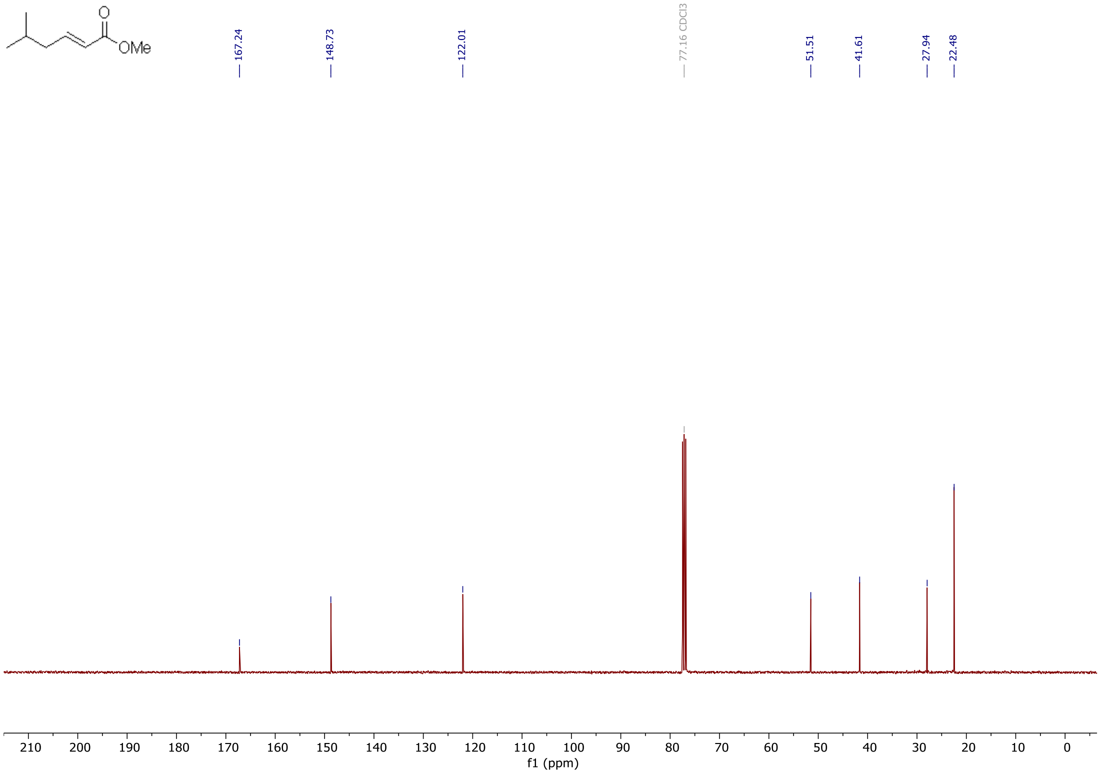

Figure S31: ^{1}H (400 MHz) and $^{13}C\{^{1}H\}$ (125.7 MHz) NMR spectra of (*S*)-5-(*N*-(2-(2-((((9*H*-fluoren-9-yl)methoxy) carbonyl)amino)-4-carboxybutanamido)ethyl)- sulfamoyl)-2-(6-(diethylamino)-3-(diethyliminio) -3*H*-xanthen-9-yl)benzenesulfonate (**17**) in CD_3OD.

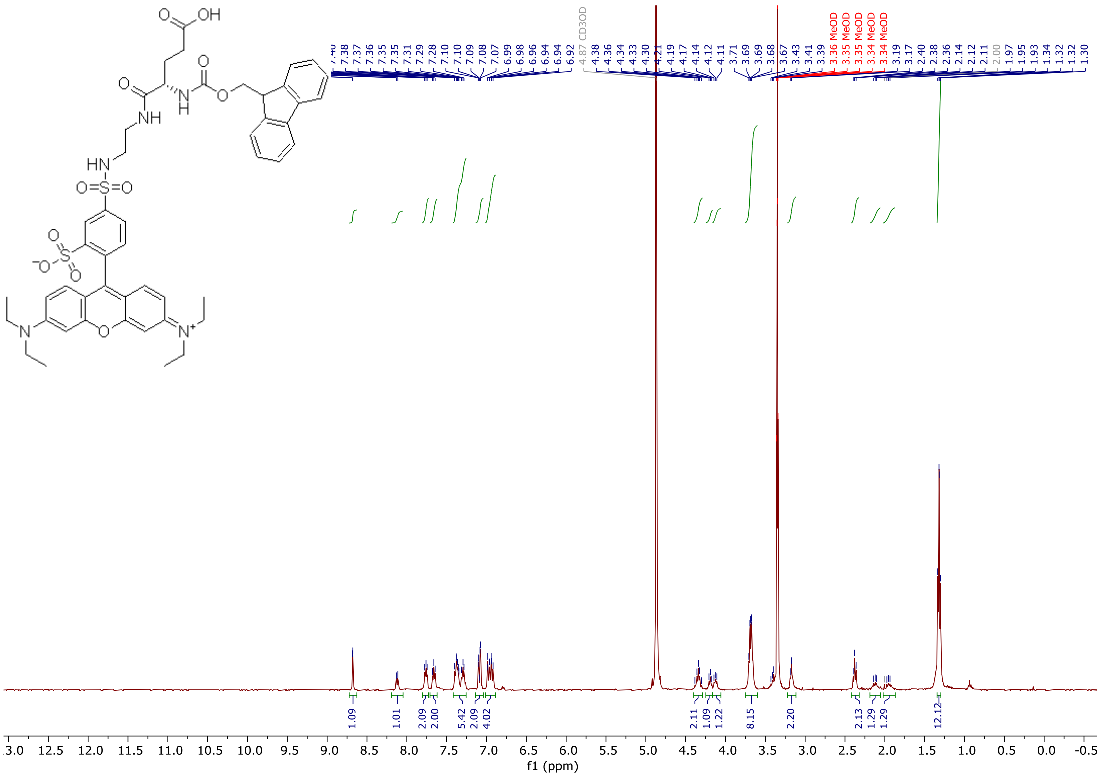

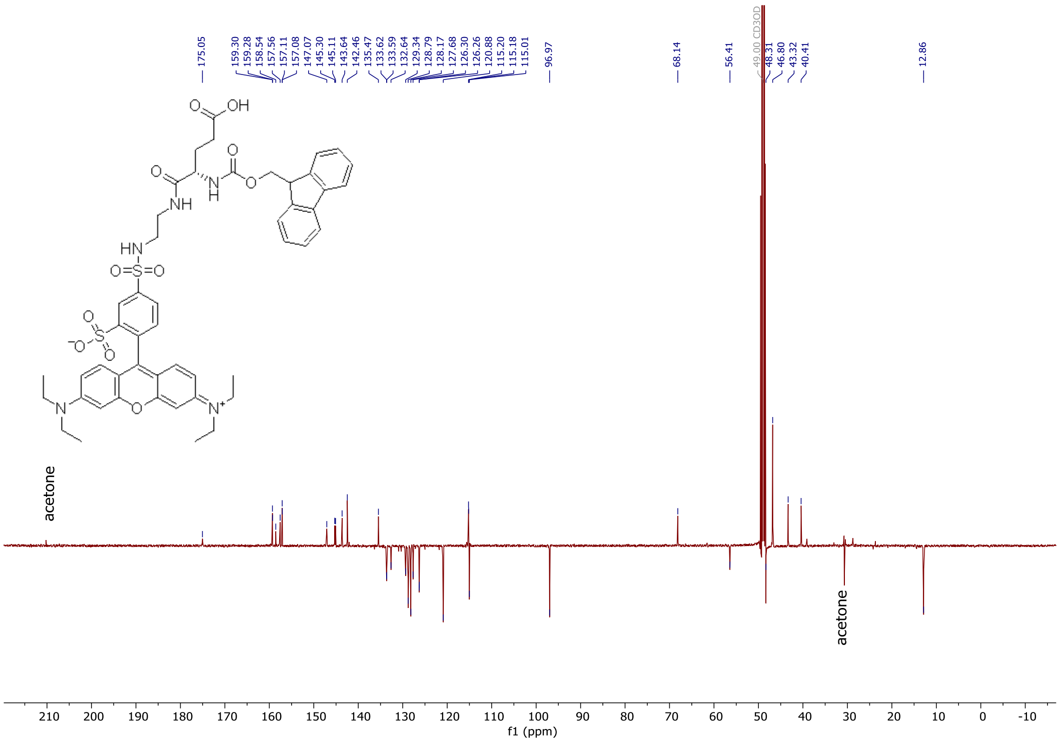

Figure S30: ^{1}H (400 MHz) and $^{13}C\{^{1}H\}$ (100 MHz) NMR spectra of 4,4',4''-((benzene-1,3,5-triyltris(methylene)) tris(oxy))tris(4-oxobutanoic acid) (**19**) in CD_3CN.

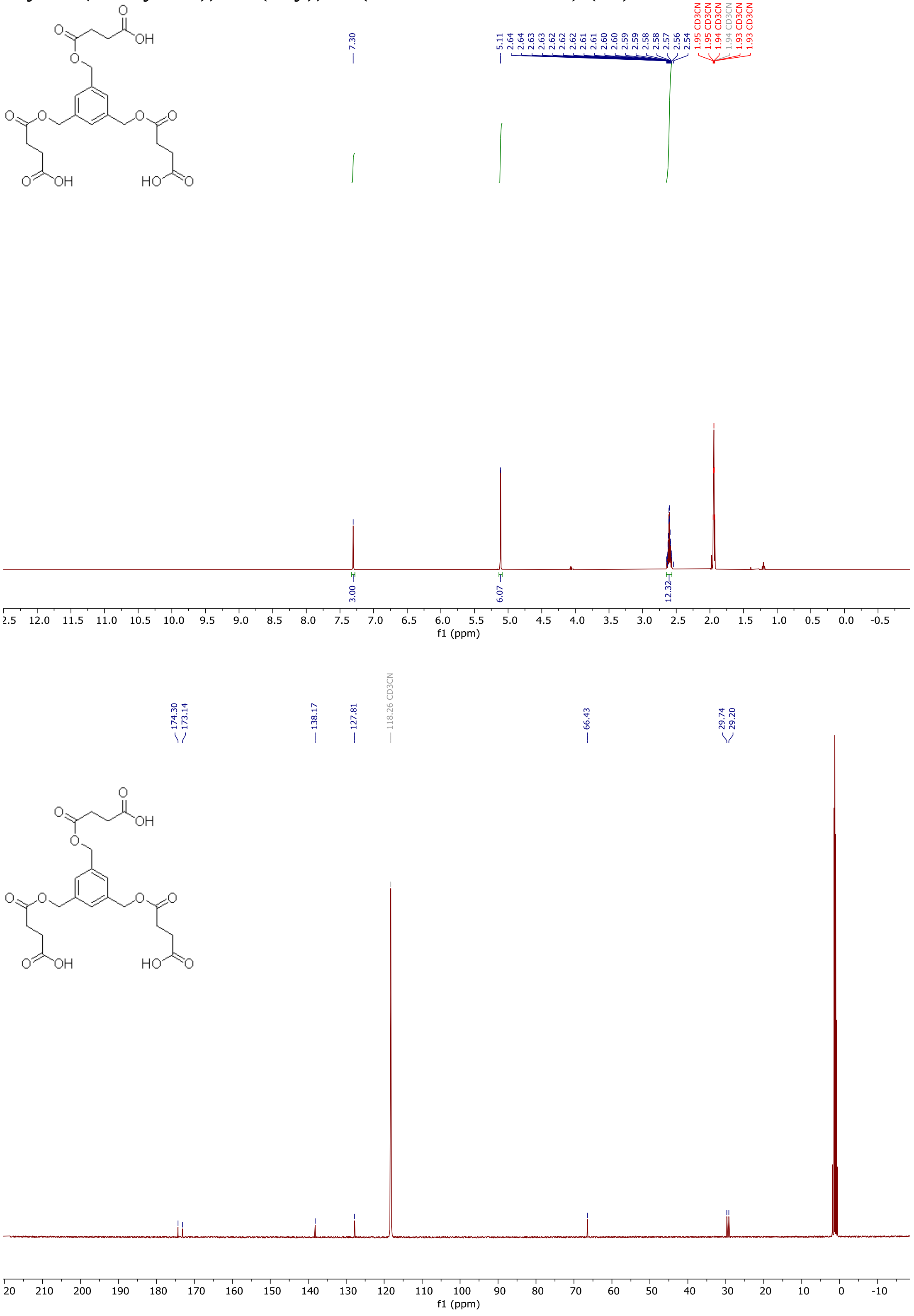

5. References

[1] Hidasová, D.; Janák, M.; Jahn, E.; Císařová, I.; Jones, P. G.; Jahn, U. Diastereoselective radical couplings enable the asymmetric synthesis of *anti*-β-amino-α-hydroxy carboxylic acid derivatives. *Eur. J. Org. Chem.* **2018**, *37*, 5222-5230.

[2] Wright, S. W.; Hageman, D. L.; Wright, A. S.; McClure, L. D. Convenient preparations of t-butyl esters and ethers from t-butanol. *Tetrahedron Lett.* **1997**, *38*, 7345-7348.

[3] Ikeda, T.; Zhang, Z.; Motoyama, Y. Hydrosilane-promoted facile deprotection of tert-butyl groups in esters, ethers, carbonates, and carbamates. *Adv. Synth. Catal.* **2019**, *361*, 673-677.

[4] Katsuyuki, I.; Kentaro, Y.; Takeshi, O. Iron(III) chloride-catalyzed reductive etherification of carbonyl compounds with alcohols. *Chem. Lett.* **2007**, *36*, 38-39.

[5] Pacherille, A.; Tuga, B.; Hallooman, D.; Dos Reis, I.; Vermette, M.; Issack, B. B.; Rhyman, L.; Ramasami, P.; Sunasee, R. $BiCl_3$-Facilitated removal of methoxymethyl-ether/ester derivatives and DFT study of -O-C-O- bond cleavage. *New J. Chem.* **2021**, *45*, 7109-7116.

[6] Davies, S. G.; Mulvaney, A. W.; Russell, A. J.; Smith, A. D. Parallel synthesis of homochiral β-amino acids. *Tetrahedron Asymmetry* **2007**, *18*, 1554-1566.

[7] Huang, Z.; Liu, Z.; Zhou, J. An enantioselective, intermolecular α-arylation of ester enolates to form tertiary stereocenters. *J. Am. Chem. Soc.* **2011**, *133*, 15882-15885.

[8] Hsiao, Y. -T.; Beadle, J.; Pascoe, C.; Annadate, R.; Vederas, J. C. Decarboxylative radical addition to methylideneoxazolidinones for stereocontrolled synthesis of selectively protected diamino diacids. *Org. Lett.* **2021**, *23*, 7270-7273.

[9] Raji, Ch.; Rajesh, R. G.; Balaji, S. V.; Chethan, N. Tris(pentafluorophenyl)borane: a mild and efficient catalyst for the chemoselective tritylation of alcohols. *Tetrahedron Lett.* **2008**, *49*, 970-973.

[10] Chandrasekhar, S.; Takhi, M.; Reddy, Y. R.; Mohapatra, S.; Rao, C. R.; Reddy, K. V. $TaCl_5$-silica gel and $TaCl_5$ as new Lewis acid systems for selective tetrahydropyranylation of alcohols and thioacetalization, trimerization and aldolization of aldehydes. *Tetrahedron* **1997**, *53*, 14997-15004.

[11] Yu, H.; Ren, J.; Xie, Y.; Su, X.; Wang, A.; Yan, L.; Jiang, F.; Wei, Y. Selective iron-catalyzed aerobic oxidation of alcohols in water to carboxylic acids mediated by additives. *Green Chem.*, **2022**, *24*, 6511-6516.

[12] Alemán, J.; del Solar, V.; Navarro-Ranninger, C. Anticancer platinum complexes as non-innocent compounds for catalysis in aqueous media. *Chem. Commun.*, **2010**, *46*, 454-456.

[13] Zhang, S.-J.; Hu, W.-X. Method for regio- and stereoselective synthesis of (*E*)-β,γ-unsaturated acids from aldehydes under solvent-free conditions. *Synth. Commun.* **2010**, *40*, 3093-3100.

[14] Kumar, G.; Godavari, A. G.; Tambat, R.; Kumar, S.; Nandanwar, H.; Sobhia, M. E.; Jachak, S. M. Synthesis, biological evaluation and computational studies of acrylohydrazide derivatives as potential Staphylococcus aureus NorA efflux pump inhibitors. *Bioorg. Chem.*, **2020**, *104*, 104225.

[15] Yao, Y.-H.; Yang, H.-Y.; Chen, M.; Wu, F.; Xu, X.-X.; Guan, Z.-H. Asymmetric Markovnikov hydroaminocarbonylation of alkenes enabled by palladium-monodentate phosphoramidite catalysis. *J. Am. Chem. Soc.*, **2021**, *143*, 85-91.

[16] Cao, D.; Xia, S.; Pan, P.; Zeng, H.; Li, C.-J.; Peng, Y. Light-driven MPV-type reduction of aryl ketones/aldehydes to alcohols with isopropanol under mild conditions. *Green Chem.*, **2021**, *23*, 7539-7543.

[17] Nakashima, E.; Yamamoto, H. Biomimetic peptide catalytic bond-forming utilizing a mild Brønsted acid. *Chem. Eur. J.*, **2022**, *28*.

[18] Sanz-Marco, A.; Blay, G.; Muñoz, M. C.; Pedro, J. R. Highly enantioselective copper(I)-catalyzed conjugate addition of 1,3-diynes to α,β-unsaturated trifluoromethyl ketones. *Chem. Comm.* **2015**, *51*, 8958-8961.
